# Bubble continuous positive airway pressure for children with high-risk conditions and severe pneumonia in Malawi: an open label, randomised, controlled trial

**DOI:** 10.1016/S2213-2600(19)30243-7

**Published:** 2019-11

**Authors:** Eric D McCollum, Tisungane Mvalo, Michelle Eckerle, Andrew G Smith, Davie Kondowe, Don Makonokaya, Dhananjay Vaidya, Veena Billioux, Alfred Chalira, Norman Lufesi, Innocent Mofolo, Mina Hosseinipour

**Affiliations:** a Eudowood Division of Pediatric Respiratory Sciences, Department of Pediatrics, School of Medicine, Johns Hopkins University, Baltimore, MD, USA; b Department of International Health, Bloomberg School of Public Health, Johns Hopkins University, Baltimore, MD, USA; c BEAD Core, Johns Hopkins University, Baltimore, MD, USA; d University of North Carolina Project Malawi, Lilongwe, Malawi; e Department of Pediatrics, School of Medicine, University of North Carolina at Chapel Hill, NC, USA; f Division of Infectious Disease, University of North Carolina at Chapel Hill, NC, USA; g Division of Emergency Medicine, Cincinnati Children's Hospital Medical Center, Cincinnati, OH, USA; h Department of Pediatrics, University of Cincinnati College of Medicine, Cincinnati, OH, USA; i University of Utah School of Medicine, Salt Lake City, UT, USA; j Malawi Ministry of Heath, Lilongwe, Malawi

## Abstract

**Background:**

Pneumonia is the leading cause of death among children globally. Most pneumonia deaths in low-income and middle-income countries (LMICs) occur among children with HIV infection or exposure, severe malnutrition, or hypoxaemia despite antibiotics and oxygen. Non-invasive bubble continuous positive airway pressure (bCPAP) is considered a safe ventilation modality that might improve child pneumonia survival. bCPAP outcomes for high-risk African children with severe pneumonia are unknown. Since most child pneumonia hospitalisations in Africa occur in non-tertiary district hospitals without daily physician oversight, we aimed to examine whether bCPAP improves severe pneumonia mortality in such settings.

**Methods:**

This open-label, randomised, controlled trial was done in the general paediatric ward of Salima District Hospital, Malawi. We enrolled children aged 1–59 months old with WHO-defined severe pneumonia and either HIV infection or exposure, severe malnutrition, or an oxygen saturation of less than 90%. Children were randomly assigned 1:1 to low-flow nasal cannula oxygen or nasal bCPAP. Non-physicians administered care; the primary outcome was hospital survival. Primary analyses were by intention-to-treat and interim and adverse events analyses per protocol. This trial is registered with ClinicalTrials.gov, number NCT02484183, and is closed.

**Findings:**

We screened 1712 children for eligibility between June 23, 2015, and March 21, 2018. The data safety and monitoring board stopped the trial for futility after 644 of the intended 900 participants were enrolled. 323 children were randomly assigned to oxygen and 321 to bCPAP. 35 (11%) of 323 children who received oxygen died in hospital, as did 53 (17%) of 321 who received bCPAP (relative risk 1·52; 95% CI 1·02–2·27; p=0·036). 13 oxygen and 17 bCPAP patients lacked hospital outcomes and were considered lost to follow-up. Suspected adverse events related to treatment occurred in 11 (3%) of 321 children receiving bCPAP and 1 (<1%) of 323 children receiving oxygen. Four bCPAP and one oxygen group deaths were classified as probable aspiration episodes, one bCPAP death as probable pneumothorax, and six non-death bCPAP events included skin breakdown around the nares.

**Interpretation:**

bCPAP treatment in a paediatric ward without daily physician supervision did not reduce hospital mortality among high-risk Malawian children with severe pneumonia, compared with oxygen. The use of bCPAP within certain patient populations and non-intensive care settings might carry risk that was not previously recognised. bCPAP in LMICs needs further evaluation before wider implementation for child pneumonia care.

**Funding:**

Bill & Melinda Gates Foundation, International AIDS Society, Health Empowering Humanity.

## Introduction

Pneumonia is the leading infectious cause of death among children aged 1–59 months worldwide.^1^ According to WHO criteria, pneumonia is a syndrome characterised by non-specific respiratory signs and symptoms, many of which occur in children without primary respiratory disease.^2^ Along with improved vaccine access, treatment according to WHO pneumonia guidelines by healthcare providers in low-income and middle-income countries (LMICs) over the past 20 years has led to reduced child pneumonia mortality globally.^3^ Now most pneumonia deaths among children aged 1–59 months in LMICs are concentrated in those with HIV infection or exposure, severe malnutrition, or hypoxaemia (peripheral oxygen saturation [SpO_2_] <90%).^4^, ^5^, ^6^

In the southern African country of Malawi, hospital mortality for children with pneumonia is 3%,^7^ while mortality of those with pneumonia and severe malnutrition exceeds 33%.^8^ Nearly 50% of child pneumonia deaths in Malawi are attributed to HIV.^6^ Children with hypoxaemia and pneumonia in Malawi are five times more likely to die than non-hypoxaemic pneumonia cases,^9^ and hypoxaemia accounts for 11% of paediatric pneumonia admissions.^7^ Alongside further improvement in WHO pneumonia guideline implementation, new treatments shown to be effective among children with high-risk conditions such as HIV, malnutrition, or hypoxaemia will be necessary to meaningfully reduce pneumonia mortality globally.^3^

**Research in context**

**Evidence before this study**

We searched Medline using the following search strategy: ((“Continuous Positive Airway Pressure”[MeSH]) AND “((“Pneumonia”[MeSH])“OR “Respiration Disorders”[MeSH])) AND ((“Developing Countries”[MeSH]) OR Ghana). The search yielded 19 studies and was completed on Jan 14, 2019. One randomised controlled trial (RCT) was published before initiating our study and evaluated the effect of nasal bubble continuous positive airway pressure (bCPAP) on children 1–59 months old with acute respiratory distress in four rural hospitals in Ghana. It found that 1 h of bCPAP administered by nurses, compared with no bCPAP, was safe and reduced mean respiratory rate. Two relevant RCTs were published while our study was being done. The first examined hospital treatment failure prevalences between three groups of children aged 1–59 months old with hypoxaemic pneumonia randomly assigned to nasal bCPAP, high-flow nasal cannula oxygen, or low-flow nasal cannula oxygen in an intensive care unit of a research hospital in urban Bangladesh. Nurses administered care under direct physician supervision. After enrolling 225 children, the trial was prematurely stopped owing to a lower relative risk (RR) of mortality among bCPAP recipients, compared with low-flow oxygen recipients, on interim analysis (0·25 RR 95% CI 0·07–0·89). The authors reported no severe adverse events attributable to bCPAP. The trial excluded children with chronic illnesses and those meeting predefined treatment failure criteria. A crossover design allowed rescue bCPAP or high-flow oxygen for children failing low-flow oxygen, and mechanical ventilation was available. The second trial was a cluster randomised crossover trial in rural Ghana that evaluated 2-week mortality after emergency ward care with and without modified nasal bCPAP among children aged 1–59 months with acute respiratory distress. Care was initiated and administered by nurses under daily physician supervision. Primary analysis found no difference in the RR of mortality between care that included bCPAP, compared with care without bCPAP (RR 0·67, 95% CI 0·42–1·08). A secondary analysis found a two times lower adjusted odds ratio of mortality among children younger than 12 months old receiving bCPAP, compared with those not receiving bCPAP (adjusted odds ratio 0·50, 95% CI 0·28–0·89). Children with more severe illness were excluded; 7% had an oxygen saturation of less than 92%. The authors reported no severe adverse events attributable to bCPAP.

**Added value of this study**

Most children with severe pneumonia in low-income and middle-income countries are cared for at non-tertiary district hospitals where intensive care is unavailable, physicians are scarce, and there is little laboratory and radiographic diagnostic support. Most pneumonia deaths in LMICs occur among children with HIV-infection or HIV-exposure, severe acute malnutrition, or hypoxaemia. We believe that this is the first trial to date to investigate the effect of nasal bCPAP on hospital survival among high-risk African children with severe clinical pneumonia at a non-tertiary district hospital where physicians are unavailable and there is no intensive care. This RCT was designed to balance the controls of a clinical trial with the representativeness of real-world care, and found that the administration of bCPAP by nurses and clinicians without daily physician oversight elevated the risk of mortality, as compared with low-flow oxygen. Few enrolled children had a nasogastric tube inserted despite nearly all being eligible for nasogastric tube placement. The influence of infrequent nasogastric tube use on these findings is unclear. Compared with oxygen, almost all subgroups had excess mortality risk from bCPAP.

**Implications of all the available evidence**

Our findings show that nasal bCPAP might pose an increased risk of mortality, compared with low-flow oxygen, for children with severe pneumonia and high-risk conditions who receive treatment in a non-tertiary LMIC district hospital. These results differ from the aforementioned trials in Bangladesh and Ghana; our trial applied wider inclusion criteria in a district hospital setting without physician oversight. These findings raise important questions about where and how bCPAP should be administered, who should administer bCPAP, and which children should receive bCPAP in LMICs. Taken together, nasal bCPAP should be administered with caution in all care settings and additional studies are needed to establish which populations are most likely to benefit from bCPAP when intensive care resources are unavailable.

Non-invasive ventilation with continuous positive airway pressure (CPAP) aims to provide constant positive airway pressure. This constant positive pressure improves lung volumes, ventilation–perfusion mismatch, and the work of breathing, thus improving gas exchange.^10^ By improving gas exchange, CPAP can reverse or avert respiratory failure.^10^ In many high-income settings, CPAP is a standard care option for children with respiratory failure, including children without hypoxaemia, although there is variability across settings and evidence is scarce.^10^ CPAP can also be used to decrease metabolic demand and increase oxygen delivery among children in shock with circulatory failure.^11^ A low cost version–bubble CPAP (bCPAP)–is used in some high-income settings and is increasingly available in LMICs for neonatal respiratory distress, including Malawi.^12^ Among neonates, bCPAP is considered a low-risk treatment with infrequent complications that can be safely administered by non-physicians.^13^ bCPAP has also been used for the care of older infants and children in Malawian tertiary hospitals since 2010. Although Malawian data to date have been observational only, and therefore inconclusive, they suggest that bCPAP complications among children with HIV or malnutrition are relatively infrequent and its use reduces the mean respiratory rate of recipients.^14^, ^15^, ^16^ A randomised trial from Bangladesh showed bCPAP to be both safe and efficacious for reducing mortality among children aged 1–59 months with hypoxaemic pneumonia in a physician-supervised intensive care unit.^17^ However, most paediatric pneumonia hospital admissions in LMICs occur at district hospitals where intensive care is unavailable, pulse oximeters for detecting hypoxaemia are absent, diagnosis by chest radiography is inaccessible, and physicians are scarce.^18^, ^19^ To optimise bCPAP's child survival effect in LMICs, bCPAP safety and efficacy must be evaluated in high-risk pneumonia cases in non-tertiary district hospitals without daily physician oversight.

We therefore did a randomised controlled trial to establish whether bCPAP, compared with low-flow oxygen, reduces hospital mortality among high-risk Malawian children with WHO-defined severe pneumonia in a general paediatric ward at a non-tertiary district hospital without daily physician supervision.^19^ We aimed to test our hypothesis that bCPAP would decrease hospital pneumonia mortality among Malawian children with high-risk conditions.

## Methods

### Study design and participants

We did an open-label, randomised, superiority trial with parallel assignment and a 1:1 allocation ratio as previously described.^20^ Eligibility was initially limited to pneumonia cases with HIV infection, HIV exposure, or severe malnutrition. We noted in a parallel observational study at the same site that hypoxaemic pneumonia without HIV or severe malnutrition occurred frequently and also conferred high mortality, but limited staffing and equipment resources restricted inclusion of these cases.^20^ This subgroup was incorporated into our study after acquiring additional funding. Details of the parallel observational study are described elsewhere.^20^

Institutional review boards in Malawi and at all investigator-affiliated institutions approved the protocol. Enrolled participants provided written consent.

Eligible children were 1–59 months old with WHO-defined severe pneumonia and one or more high-risk conditions (HIV infection or exposure, severe malnutrition, hypoxaemia; appendix pp 1–3). Severe pneumonia was defined in two ways, consistent with WHO definitions.^21^ Specifically, children could meet eligibility criteria either by having signs of severe pneumonia with or without a general danger sign or by having signs of pneumonia with a general danger sign. If a child had been enrolled in the study and discharged, and then presented again to the study site, they were ineligible. Otherwise, no other exclusion criteria were applied.

The trial was done in the general paediatric ward of Salima District Hospital in Salima, Malawi, a 250-bed government hospital. Intensive care services, mechanical ventilation, and portable radiography were unavailable. Study staff were available at all times and included non-physician clinicians called clinical officers, nurses, vital sign assistants, and HIV counsellors. Clinical officers undergo 3 years of medical education including an internship year. To optimise protocol adherence, we trained staff on procedures before the study over a 5-day period, including the physiology, application, and monitoring of bCPAP, did twice-annual refresher courses, and provided on-site paediatrician supervision once every 2 weeks. During training and supervisory visits, clinical staff were evaluated on their clinical evaluation of patients, use of decision algorithms, and decision making during directly observed care provision. Remediation took place as necessary. A study paediatrician based in Malawi was available for telephone consultation at all times. All study nurses had at least 1 year of previous bCPAP treatment experience at the referral hospital in Lilongwe, Malawi. The nurse study coordinator had 5 years of bCPAP treatment experience. The trial provided all respiratory equipment including oxygen concentrators, bCPAP devices, and pulse oximeters, and also installed an electrical generator, and supplemented medicines.

### Randomisation and masking

This open-label trial used a computer-generated sequence for simple randomisation; the principal investigator kept the schedule. Random assignment at enrolment was established by means of sealed, sequentially numbered opaque envelopes and verified by two study staff.

### Procedures

Because this study aimed to reflect real world care in LMIC non-tertiary district hospitals, investigators carefully deliberated all study procedures to optimise programmatic feasibility and safety. Study procedures have already been described in detail.^20^ Briefly, children received routine triage and clinical care during consent and were enrolled 24 h per day including weekends. After enrolment, children were immediately placed on the treatment prescribed by randomisation. All study participants met WHO clinical criteria for oxygen use in settings lacking pulse oximetry. SpO_2_ was measured in room air by a Rad5 pulse oximeter (Masimo, Irvine, CA, USA) for trial purposes only and participants received bCPAP or low flow oxygen regardless of SpO_2_.^21^ This approach was chosen to reflect typical conditions in non-tertiary district hospitals where pulse oximeters are not routinely available and oxygen administration is usually based solely on clinical criteria. The protocol did not permit crossover between groups; children deteriorating on oxygen did not receive rescue bCPAP as bCPAP efficacy had not been shown, thus contributing to the equipoise for this trial.

All participants were assessed for malaria by means of SD Bioline (HRP2/pLDH) rapid antigen tests (Abbott, Johannesburg, South Africa) and anaemia by point-of-care haemoglobin tests. From May, 2017, children with positive rapid antigen results for malaria also had a smear obtained and processed at the University of North Carolina Project laboratory, a US Centers for Disease Control and Prevention-certified laboratory, in Lilongwe, Malawi. Children received antibiotics, intravenous fluids, antimalarials, antipyretics, blood transfusions, and hypoglycaemia treatment per WHO and national guidelines.^21^ Specifically, all children without signs of meningitis were initiated on 50 000 U/kg parenteral benzylpenicillin every 6 h and 7·5 mg/kg parenteral gentamycin daily; those with an abnormal Blantyre Coma Score or stiff neck were initiated on 100 mg/kg parenteral ceftriaxone daily. Children testing positive by rapid antigen testing for malaria received 2·4 mg/kg parenteral artesunate daily and antibiotics, were switched to oral artemether–lumefantrine after at least 24 h. In addition to first-line antibiotics, HIV-infected or HIV-exposed children received oral co-trimoxazole (8 mg/kg trimethoprim) every 8 h and 2 mg/kg daily oral prednisone for presumptive *Pneumocystis jirovecii* infection. Consistent with WHO guidelines, children with persistent fever or worsening respiratory status by day three of hospitalisation were switched to 80 mg/kg parenteral ceftriaxone daily and those with persistent illness despite 5 days of first-line antibiotics were switched to 80 mg/kg parenteral ceftriaxone daily by day 6. Participants with a general danger sign, apnoea, grunting, or at least three respiratory danger signs were fed by nasogastric tube and not permitted oral intake. Clinicians and nurses did full clinical evaluations of each child at least twice daily. Staff did vital signs and a focused respiratory assessment of children at least every 6 h, including inspecting the nasal interface for patency. Staff did additional evaluations and interventions following standard of care for Malawi district hospitals.

For participants receiving low-flow oxygen, we used the Airsep Newlife Intensity oxygen concentrator (Chart Industries, New York, USA) to deliver oxygen through a flow splitter. The oxygen group initially received 0·5 L/min if aged 30–59 days and 2 L/min if aged 2–59 months through nasal prongs. Initial settings followed WHO and national guidelines.^21^

For participants receiving bCPAP, we used a validated bCPAP system (Fisher and Paykel Healthcare, Auckland, New Zealand), which provides warmed humidification and pressure control. As in all bCPAP systems, pressure was maintained via the depth of expiratory tubing in the system's water reservoir, and airflow was titrated to achieve bubbling in the reservoir. Airflow was delivered via an Airsep Newlife Intensity oxygen concentrator at 6–8 L/min. Participants were initiated on 7 cm H_2_O nasal bCPAP for children aged 30–59 days and 8 cm H_2_O nasal bCPAP for children aged 2–59 months. Although the bCPAP pressure ranges between 5 cm H_2_O and 8 cm H_2_O were determined before the trial, the use of 7 cm H_2_O and 8 cm H_2_O as the initial pressure setting was established between June and August, 2015, when it was observed that nine of the first ten enrolled bCPAP recipients required such pressures to stabilise or improve their respiratory danger signs and it was infeasible for staff to frequently titrate the pressure settings and follow up to evaluate the clinical response. Previous literature on children reports that CPAP initiation with pressures between 5 cm and 10 cm H_2_O is safe.^22^, ^23^ We used either unvented nasal masks or nasal prongs as the nasal interface, with a preference towards nasal masks given our previous experience of frequent nasal septal trauma and nose bleeds attributed to nasal prong use.^14^ In the event of a power outage, the nasal mask or prongs were immediately removed until the electrical generator was started.

Following initiation, respiratory support for both groups was weaned in 24 h increments based on step-wise procedures published previously.^20^ We established 24 h weaning procedures based on our initial observations that frequent bCPAP pressure titration was not feasible in this setting. Specifically, bCPAP and low-flow oxygen weaning was only permitted for children who after 24 h of hospitalisation had a decrease in respiratory danger signs. Hypoxaemia, grunting or apnoea were considered life threatening and weaning was not permitted if any of these were present. All children were re-evaluated by clinical staff 60 min after any change in bCPAP or low-flow oxygen delivery. Children were escalated back to the previous level of respiratory support if new danger signs or hypoxaemia developed after weaning. If not already at maximum low-flow or bCPAP support, children with new danger signs or hypoxaemia were escalated at all other times during the trial. For both groups, low-flow oxygen was the final step in weaning and was only stopped when all respiratory danger signs were resolved.

The nasal interface was examined for patency and fit and nasal suctioning by means of a catheter and normal saline was done on deteriorating patients or patients with any bCPAP or oxygen change. Chest radiography was done on children with persistent or worsening illness if transportation to the radiography department could be safely completed, as is typical in LMIC district hospitals. Between patient uses, respiratory equipment was decontaminated and reused per Malawi guidelines (see appendix p 81), consistent with programmatic conditions.

### Outcomes

The trial's predefined primary outcome was survival to hospital discharge. Predefined secondary outcomes included antibiotic and hospital treatment failure, 30-day post-discharge vital status, and hospital adverse events. Among those alive on day 14 of hospitalisation, hospital treatment failure is defined as the presence of any one of the following: temperature ≥38 °C, any respiratory danger sign, or continued oxygen or bCPAP requirement. Events were graded per DAIDS AE Grading Table version 1.0 by the investigators and were verified by the data and safety monitoring board (DSMB). Outcomes were stratified by prespecified patient characteristics including SpO_2_, age, sex, anaemia, wheeze, mental status, diarrhoea, and malaria.

### Statistical methods

With a sample size of 900 participants, the trial had 80% power to detect a 6·0 percentage point lower mortality prevalence with bCPAP, assuming 14·7% mortality with standard care at a two-sided α level of 0·05. Sample size calculations and assumptions were described in detail previously.^20^ Per-protocol interim DMSB analyses were done at 30% and 60% enrolment. O'Brien-Fleming stopping thresholds of 0·0006 were prespecified for the first interim analyses and 0·0151 for the second interim analyses.

The DSMB recommended futility analysis at the second interim point. In this context futility was calculated as a 0·0026% probability that bCPAP might achieve a significantly lower mortality risk, compared with oxygen, if enrolment continued to the target sample size.

We assessed unadjusted treatment effects on primary and secondary outcomes in the main and subgroup analyses by means of Stata's epitab package and log binomial regression to estimate relative risks (RRs). We tested subgroup by treatment interaction (heterogeneity of treatment effect) by means of Poisson regression because some models did not converge. Final analyses were intention-to-treat. We summarised continuous data with means and SDs and compared differences by Student's *t* tests or non-parametric rank sum tests based on data distribution. Categorical data was reported by means of the number and proportion within each category and comparisons by Pearson χ^2^ tests. We also did ad-hoc exploratory analyses that included time to hospital death by study group, fidelity implementation outcomes per group allocation, baseline characteristics and fidelity implementation outcomes by hospital outcome, and association between study year and hospital outcome by treatment group. We used non-parametric log-rank tests for the survival analysis, and Cox regression analyses to estimate the treatment hazard ratio (HR) and its 95% CI. This study is registered with ClinicalTrials.gov, number NCT02484183.

### Role of the funding source

The sponsors had no role in the design, implementation, analyses, interpretation, write up, or decision to publish. The corresponding author had access to all data and takes responsibility for the manuscript.

## Results

**Figure 1:**
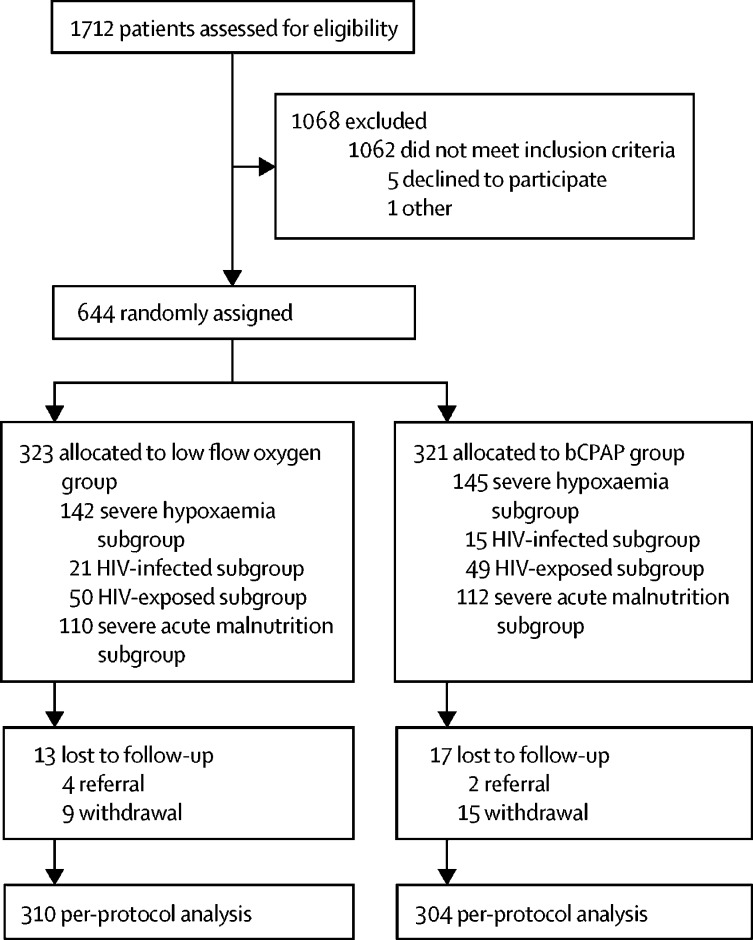
Trial profile bCPAP=bubble continuous positive airway pressure.

We screened children for eligibility between June 23, 2015, and March 21, 2018; children with severe hypoxaemia who were without HIV or severe malnutrition were included after May, 2016. 644 children had a co-existing high-risk condition and were randomly assigned with 323 allocated to low-flow oxygen and 321 to bCPAP (figure 1; table 1). 17 bCPAP and 13 oxygen patients lacked hospital outcomes and were considered lost to follow-up. Specifically, four low-flow oxygen children and two bCPAP children were referred to a tertiary hospital and nine low-flow oxygen children and 15 bCPAP children withdrew from the trial.

**Table 1:** Baseline characteristics

		**Low-flow oxygen (n=323)**	**bCPAP (n=321)**
**Demographic and anthropomorphic characteristics**			
Age, months		7·7 (3·4–15·1)	7·6 (3·0–15·6)
Sex			
	Female	149 (46%)	150 (47%)
	Male	174 (54%)	171 (53%)
Weight, kg		7·3 (2·9)	7·0 (2·7)
MUAC		12·7 (1·9)	12·7 (1·8)
**High risk condition**			
HIV infected		21 (7%)	15 (5%)
HIV exposed		50 (15%)	49 (15%)
Severe acute malnutrition without HIV infection or exposure		110 (34%)	112 (35%)
SpO_2_ <90% without HIV infection, HIV exposure, or severe acute malnutrition		142 (44%)	145 (45%)
**Findings at presentation**			
Axillary temperature, °C*		37·5 (1·0)	37·4 (1·1)
Axillary temperature ≥38·0°C*		91/322 (28%)	81/320 (25%)
Pulse rate, beats per min		165 (24)	165 (24)
Systolic blood pressure, mm Hg†		106 (15)	101 (17)
Respiratory rate, breaths per min		62 (15)	62 (15)
SpO_2_		88% (84–95)	87% (81·5–94)
SpO_2_ 93–100%		105 (33%)	86 (27%)
SpO_2_ 90–92%		22 (7%)	16 (5%)
SpO_2_ <90%		196 (61%)	219 (68%)
Any respiratory danger sign		319 (99%)	319 (99%)
Severe lower chest wall indrawing		284 (88%)	291 (91%)
Head nodding or tracheal tugging		84 (26%)	92 (29%)
Grunting		78 (24%)	76 (24%)
Very fast breathing for age‡		107 (33%)	104 (32%)
Stridor while calm		5 (2%)	8 (2%)
Apnoea		16 (5%)	21 (7%)
Nasal flaring		242 (75%)	229 (71%)
Any general danger sign		55 (17%)	65 (20%)
Convulsions		26 (8%)	35 (11%)
Inability to feed		36 (11%)	42 (13%)
Vomiting everything		8 (2%)	11 (3%)
Blantyre Coma Score ≤4		20 (6%)	35 (11%)
Crackle without wheeze		151 (47%)	160 (50%)
Wheeze with or without crackle		68 (21%)	60 (19%)
Severe anaemia*§		29 (9%)	36 (11%)
Diarrhoea		14 (4%)	10 (3%)
Dehydration		17 (5%)	16 (5%)
**Laboratory assessments**			
Haemoglobin, g/dL*		9·6 (2·5)	9·4 (2·7)
Malaria rapid antigen positive		89 (28%)	108 (34%)

Data are median (IQR), n (%), and mean (SD). bCPAP=bubble continuous positive airway pressure. MUAC=mid-upper arm circumference. SpO_2_ =peripheral arterial oxyhaemoglobin saturation.

* 322 and 320 participants in the low-flow oxygen group and bCPAP group had an axillary temperature measured and 320 in the bCPAP group had a haemoglobin sample collected.

† 98 and 96 participants in the low-flow oxygen group and bCPAP group had a systolic blood pressure collected.

‡ Very fast breathing for age defined as ≥80 breaths per min for children aged 30–59 days, ≥70 breaths per min for children aged 2–11 months, and ≥60 breaths per min for children aged 12–59 months.

§ Severe anaemia was a haemoglobin <6 g/dL in children without severe acute malnutrition and <4 g/dL in children with severe acute malnutrition.

88 (14%) children died while hospitalised; 35 (11%) deaths occurred among the oxygen group and 53 (17%) among the bCPAP group (table 2). Children randomly assigned to bCPAP, compared with oxygen, had a higher relative risk of hospital death (RR 1·52, 95% CI 1·02–2·27; p=0·036).

**Table 2:** Hospital outcomes

	**Low-flow oxygen (n=323)**	**bCPAP (n=321)**	**Relative risk**	**95% CI**	**p value**
Alive	275 (85%)	251 (78%)	0·92	0·85–0·99	0·023
Dead	35 (11%)	53 (17%)	1·52	1·02–2·27	0·036

Data are n (%), unless otherwise stated. bCPAP=bubble continuous positive airway pressure.

In analysis of prespecified secondary outcomes, suspected adverse events related to bCPAP or oxygen occurred in 11 (3%) of 321 children receiving bCPAP and 1 (<1%) of 323 children receiving oxygen (appendix pp 4–9). Four bCPAP and one oxygen group death were classified as probable aspiration episodes, one bCPAP death as probable pneumothorax, and six non-death bCPAP events included skin breakdown around the nares. The RR of 2-week hospital treatment failure in the bCPAP group, compared with the oxygen group, was 1·32 (95% CI 0·96–1·83); p=0·081; appendix p 13). Among children discharged from the hospital, the prespecified RR 30-day post discharge mortality in the bCPAP group, compared with the oxygen group was 1·42 (95% CI 0·24–8·39; p=0·70; appendix p 16).

We also examined hospital mortality prevalences among children with and without hypoxaemia and malaria (appendix pp 10–11). Among all 415 hypoxaemic children, the bCPAP group had a higher RR of death compared with the oxygen group (1·65 RR, 95% CI 1·00–2·75; p=0·048). The results from the 229 non-hypoxaemic children (SpO_2_ 90–100%) were not significant and did not suggest a benefit from bCPAP, compared with oxygen (RR 1·32, 95% CI 0·69–2·55; p=0·39). The RR of hospital death among 144 hypoxaemic bCPAP recipients with no other high-risk condition was 2·67 (95% CI 1·16–6·16; p=0·021), compared with the 142 hypoxaemic oxygen recipients with no other high-risk condition. Analyses of other hypoxaemic subgroups did not reach significance (appendix pp 10–11).

Although prespecified analysis by malaria status was inconclusive it did not suggest children with malaria benefited from bCPAP, compared with oxygen (appendix pp 10–11). The RR of death from bCPAP, compared with oxygen, among all 197 children that tested rapid malaria positive was 1·26 (95% CI 0·70–2·27; p=0·44). Among 447 children with a negative rapid malaria test, the RR of death from bCPAP versus oxygen was 1·64 (95% CI 0·96–2·81; p=0·067). To understand whether a positive rapid antigen test represented a true case of malaria, over the final 12 months of the trial, malaria smears were collected on all patients testing rapid malaria positive and analysed at an internationally certified laboratory. 21 (24%) of 89 children in the oxygen group who tested rapid malaria positive had a malaria smear, and only three (14%) of 21 were smear positive. Among 108 bCPAP children with positive rapid malaria tests, 24 (22%) of 108 had a malaria smear collected and only four (17%) of 24 were smear positive. One oxygen patient who was smear positive and no bCPAP patients who were smear positive died in the hospital.

Although almost all other prespecified high-risk subgroup analyses showed excess mortality for bCPAP, compared with oxygen, none were statistically significant. We also compared the RR for children who did and did not have a condition of interest to examine whether any subgroup had a differential treatment effect from bCPAP (heterogeneity of treatment effect), and we found no significant difference for any subgroup (presence *vs* absence of condition, appendix pp 10–11). All other secondary endpoints are in the appendix pp 12–15.

**Figure 2:**
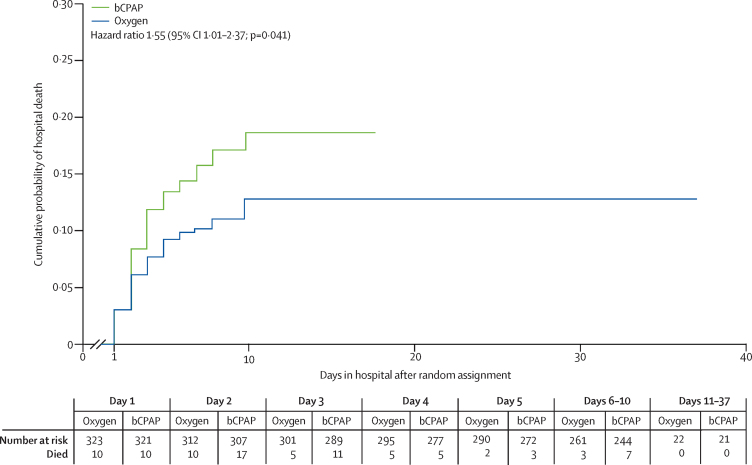
Kaplan-Meier mortality curves Time to hospital death was an exploratory analysis. Day 37 was the latest day that a child had a hospital outcome in the oxygen group.

An exploratory survival analysis did not suggest delayed hospital mortality among either group (figure 2). Children randomly assigned to bCPAP had a 1·55 HR for hospital death (95% CI 1·01–2·37; p=0·041) compared with patients in the oxygen group. Most excess bCPAP hospital mortality (13 (72%) of 18) occurred on days two and three post-random assignment.

We report additional implementation outcomes that measured fidelity of the treatment to care protocols in table 3 and also exploratory analyses in the appendix pp 16–18. Of note, although 619 (96%) of 644 children enrolled onto the trial met nasogastric tube insertion criteria, records indicated that only 101 (16%) of 619 participants received one. A 7% lower proportion of bCPAP children (40 (13%) of 308), compared with oxygen children (61 (20%) of 311), were documented to have had a tube inserted (p=0·024). To assess whether respiratory equipment might have deteriorated over the course of the study we evaluated whether there was any interaction between study year and the odds of hospital mortality by trial group, and we observed no significant interaction (p=0·20).

**Table 3:** Implementation outcomes

	**Low-flow oxygen (n=323)**	**bCPAP (n=321)**	**p value**
**Nasogastric tube feeding**			
Eligible for nasogastric tube	311 (96%)	308 (96%)	0·84
Nasogastric tube inserted	61 (20%)	40 (13%)	0·024
**Antibiotic switch**			
Alive and eligible for antibiotic switch at hospital day 6*	225/283 (80%)	184/266 (69%)	0·0056
Antibiotic switched	225/225 (100%)	184/184 (100%)	1·0
**Respiratory support**			
Length of respiratory support in days†	3·9 (2·1)	4·5 (1·9)	0·0007
**Fluid support for diarrhoea-associated dehydration**			
Dehydrated	17 (5%)	16 (5%)	0·87
Fluid resuscitated‡	17 (100%)	16 (100%)	1·0
**Time of death**			
Died at night§	25/35 (71%)	24/53 (45%)	0·018

Data are n (%) or mean (SD). bCPAP=bubble continuous positive airway pressure.

* 40 oxygen and 55 bCPAP children died or were lost-to-follow up by day six of hospitalisation.

† Among 275 oxygen and 251 bCPAP children that survived to hospital discharge.

‡ Six oxygen children and six bCPAP children were classified as severe dehydration and all six oxygen children and six bCPAP children were documented as receiving at least one 10 cm^3^/kg intravenous fluid bolus.

§ Defined as between 1700 h and 0759 h.

## Discussion

Using a randomised trial design implemented in a non-tertiary district hospital without physicians, we assessed the effect of bCPAP compared with low-flow nasal cannula oxygen for African children with severe pneumonia and at least one high-risk condition. Compared with oxygen, we found that bCPAP not only provided no benefit but, more alarmingly, its use was associated with an increased risk of mortality among this patient population. These results should be interpreted with caution given the possible overestimation of treatment effect on mortality that might occur when trials are stopped prematurely.^24^

Since the study was stopped prematurely by the DSMB, the predefined subgroups were individually underpowered to detect a mortality risk. Although severely malnourished children overall had high hospital mortality (20%), the RR of mortality among bCPAP recipients, compared with oxygen recipients, was 1·07 (95% CI, 0·64–1·79; p=0·79; appendix p 7). This RR was modest compared with other prespecified subgroups. On the basis of these findings there does not seem to be much additional risk in the use of bCPAP, compared with oxygen, among severely malnourished children in this trial. However, almost all subgroups had point estimates suggesting excess bCPAP mortality. Taken together, these results do not support bCPAP treatment of these high-risk children in similar LMIC settings without further evidence. Our findings instead suggest that bCPAP might not be safe for high-risk children at LMIC non-tertiary district hospitals. It is also unclear if bCPAP has a detrimental effect on mortality risk among similar high-risk cohorts in settings where physician supervision is available. Further controlled studies are needed.

Previous bCPAP trials either restricted high-risk children's inclusion to an intensive care unit under daily physician supervision or enrolled less-sick children.^17^, ^25^ Interpretation of the results of previous trials and our results must be taken in this context. A randomised trial of 146 hypoxaemic Bangladeshi children aged 1–59 months in an intensive care unit of one research hospital found a 0·25 RR of hospital mortality from nasal bCPAP, compared with low-flow oxygen.^17^ However, this trial relied on physician-supervised intensive care, escalated children failing treatment to mechanical ventilation, and excluded children with treatment failure at enrolment, an important subgroup that, in our experience, would usually be prioritised for bCPAP in real-world settings where mechanical ventilation is unavailable. A cluster randomised trial in Ghana compared modified bCPAP with routine care among 2181 children aged 1–59 months with respiratory distress receiving care from nurses under daily physician supervision at two district hospitals.^25^ No difference in mortality was seen in the primary unadjusted analysis, although secondary adjusted analyses reported a 0·40 RR of mortality among children younger than 12 months receiving bCPAP, compared with routine care. In contrast, in our trial children younger than 12 months old had an elevated RR of mortality compared with children aged 12 months and older (RR 1·70, 95% CI 1·00–2·90; appendix p 10). However, the Ghana trial excluded severely ill children for whom bCPAP would probably be used in real-world settings and although nurses led care provision, physicians were available for daily supervision of care. Further, the trial's modified bCPAP was different to standard bCPAP in that a conventional CPAP machine was used as the flow-driver.

In our study, we attempted to balance internal and external validity by maintaining the controls of a clinical trial while observing implementation in a real-world setting. This unique design optimises the public health effect of trials and has been classified by others as a type 1 hybrid implementation effectiveness design in which the health outcome is the primary endpoint but implementation is also monitored.^26^ We carefully considered external validity in our preparations, and several purposeful design decisions might have affected the trial results.

First, we focused on severely ill children representative of most hospital severe pneumonia deaths in LMICs and limited the exclusion criteria to only patients previously enrolled in the study. Under real-world conditions and in the absence of mechanical ventilation (typical of conditions in LMICs), many providers would use bCPAP for all children with respiratory danger signs. We also acknowledge that providers using CPAP in other settings might not treat similar patients with CPAP based on their own care practices.

Second, we did our trial in an environment that reflected real-world personnel conditions where non-physician providers delivered all patient care without daily physician supervision. Our approach was ethically sound on the basis of CPAP's strong safety record for neonates under the care of non-physicians, including in Malawi, and the fact that physicians rarely provide daily care in non-tertiary LMIC hospitals.^13^, ^14^

Third, our study used 6–8 L/min of unblended oxygen to deliver bCPAP pressure. The resources required to titrate air–oxygen delivery to achieve the flow required for bCPAP are expensive and beyond what most LMIC district hospitals can sustainably provide. Commercial oxygen concentrators, on the other hand, are commonly available in LMIC district hospitals, are recommended by WHO,^27^ and generate enough flow to drive bCPAP. However, commercial oxygen concentrators generate flow with an FiO_2_ greater than 90%, do not blend oxygen with medical air unless altered post manufacturing, and are incompatible with air–oxygen blenders owing to inadequate pressure.^27^ In order to achieve adequate pressures, bCPAP children were exposed to higher flows of inspired FiO_2_ than low-flow-oxygen children. Although difficult to accurately estimate, we acknowledge that bCPAP children were possibly exposed to FiO_2_ greater than 60%, which has the theoretical toxicity of reactive oxygen species, such as pulmonary inflammation and adsorption atelectasis, as well as to a longer duration of respiratory support than oxygen group children.^28^ Interestingly, the Bangladesh trial, which showed improved survival from bCPAP, also used a commercial oxygen concentrator with FiO_2_ greater than 90% to drive flow, and their patients might have been exposed to similar amounts of oxygen.^17^ Since the Bangladesh trial used standard oxygen nasal prongs to deliver bCPAP, rather than commercial CPAP prongs or masks, more entrainment of room air might have occurred to reduce FiO_2_ below possible percentages delivered in our trial. However, if the nasal interface was not tightly sealed, then the positive end expiratory pressure is also likely to have been lower. Although improbable, we cannot exclude oxygen toxicity as a possible factor in bCPAP deaths.

Fourth, we used a validated bCPAP device whose components are deemed single use by the manufacturer. Because of budgetary constraints and supply chain issues in Malawi and other LMICs, respiratory supplies are in our experience almost always disinfected and reused during routine care, and we took this approach.^29^ Single use supplies are usually unsustainable during programmatic conditions. Since most bCPAP mortality occurred within 3 days and the odds of bCPAP mortality, relative to oxygen mortality, did not change by study year, equipment contamination was unlikely to be a mortality driver in this trial.

There are other possible explanations for our findings. Infants and children with respiratory danger signs might be at an increased risk of swallowing impairment and aspiration.^30^ Evidence regarding whether nasal CPAP increases aspiration risk is scarce.^31^, ^32^ Nevertheless, to reduce aspiration risk, high-resource setting CPAP recipients are often fed continuously through nasogastric or post-pyloric tubing, a practice rare in LMICs. Before this trial, Malawian children with respiratory danger signs were fed orally; only comatose children were bolus fed through nasogastric tubing. Additionally, in our clinical experience in Malawi, errors related to the volume or type of intravenous fluid administered are common and can have serious health consequences. Given these safety concerns and that enteral feeds is standard of care in most settings for children on CPAP, we opted to administer maintenance hydration and enteral feeds to children using nasogastric tubes when respiratory distress was substantial. Although study staff were trained to use nasogastric tubes, the proportion of eligible children with a tube placed was low and bCPAP recipients, compared with oxygen recipients, were even less likely to have a tube inserted. An analysis of outcomes for patients with or without a nasogastic tube is given in the appendix (p 18). Unfortunately, our routine supervisory visits did not appreciate the degree to which nasogastric tubes were not used by providers or refused by parents. Notably, these data are unable to establish whether tube insertion was low because of inaccurate documentation, parental refusal, or health-care provider reluctance (possibly owing to difficulty inserting the tube when the CPAP nasal interface was in place), nor is it clear whether the tube was used once inserted. Although speculative, these trial data might suggest that more unrecognised aspiration-related events occurred and could have differentially contributed to bCPAP mortality. Although we did not observe frequent issues with gastric distension and vomiting, it is also possible that the combination of higher initiating CPAP pressures, enteral feeding, and low proportion of nasogastric tube use among bCPAP recipients predisposed patients to unrecognised aspiration events. Our findings might not be applicable to settings that have more widely adopted the use of nasogastric tubes.

Pneumothoraces might also have contributed to bCPAP deaths. Although paediatric data are scarce, neonatal bCPAP studies report pneumothoraces as infrequent, ranging between 0% and 7%.^33^ Our initiation bCPAP pressures (7–8 cm H_2_O) were unlikely to have posed exceptional risk.^22^, ^23^ We also monitored delivered pressures by gauge manometry at least twice daily to ensure pressures did not persistently exceed these amounts. In the event of low pressures, staff evaluated the fit of the nasal interface and observed for mouth leak. No interventions such as chin straps or other modalities were used to mitigate mouth leak in this trial. Owing to the unavailability of portable radiography, we relied on clinical examinations to detect pneumothoraces in patients considered unsafe for transportation to the radiology department, which was most patients. This approach was deliberated extensively before the trial and it was ascertained to be ethical—given bCPAP's strong safety record among neonates—and commensurate with real-world bCPAP care since most district hospitals do not have portable radiography or 24-h access to radiologists for urgent interpretations. We identified only one probable pneumothorax among bCPAP recipients and none among oxygen recipients, suggesting that either pneumothoraces were missed or that they were not a key explanation for bCPAP mortality.

Unlike other trials that used CPAP nasal prongs exclusively,^17^, ^25^ this trial used unvented nasal masks or CPAP nasal prongs for the nasal interface of bCPAP children. Although study staff inspected the nasal interface for any obstruction as a part of their routine evaluations and in any deteriorating patients, it is possible that unrecognised mispositioning of the mask or obstruction of the nasal prongs might have led to the development of hypoxaemic or hypercapneic respiratory failure in some children. We observed that a higher proportion of oxygen children died at night, compared with bCPAP children, and we speculate that this might reflect respiratory vulnerability during sleep that is insufficiently addressed by low-flow oxygen.

Several additional limitations require discussion. Although the trial design intentionally sought to replicate real-world settings as much as possible, we did augment clinical staffing, supplement local equipment, and provide fortnightly in-person paediatrician supervision. This was done to ensure the degree of internal validity expected of rigorous efficacy trials. The augmented clinical support might imply that similar LMIC district hospitals could not implement bCPAP without substantial investment, or that only select, better-resourced hospitals would have the capacity for bCPAP in existing programmatic conditions.

Next, WHO severe pneumonia is a clinical diagnosis that does not require chest radiographs or clinical auscultation to confirm the presence of lower respiratory disease. The signs and symptoms of WHO severe pneumonia are non-specific and can be found in children without primary respiratory disease, including those with circulatory failure.^2^ To pragmatically assess the effect of bCPAP as it would be administered in real-world conditions, this trial purposefully included children diagnosed with WHO severe pneumonia. Given that WHO severe pneumonia is a non-specific diagnosis, some of the trial's patients probably had signs and symptoms driven primarily by non-respiratory disease such as shock from malaria or sepsis. Respiratory support, including CPAP, is recommended for such children with significant circulatory failure as preload is being optimised or inotropic support initiated.^11^ Cardiopulmonary interactions must be considered whenever applying bCPAP to patients. By increasing intrathoracic pressure, CPAP can decrease cardiac preload which can adversely affect some patients in shock. In high-resource settings, CPAP is widely used for patients in shock in intensive care settings with direct physician oversight and careful evaluation of preload and cardiac output. As previously discussed, WHO-defined severe pneumonia is a non-specific diagnosis that can include patients in shock. In this trial, we followed WHO guidelines for the treatment and evaluation of patients with signs of shock. However, given the resources of a district hospital, monitoring of such patients was less than would be done in a typical intensive care or high dependency unit. Therefore, we cannot exclude cardiopulmonary interactions from bCPAP as a contributor to our results. Our study was intentionally not designed, and owing to premature study termination inadequately powered, to differentiate bCPAP efficacy between patients with respiratory or circulatory failure, or both, and our results should be interpreted within this context.

Finally, we did not expect to find higher bCPAP mortality. As a result, the trial was not designed and did not have the resources to explicitly evaluate whether aspiration, oxygen toxicity, equipment contamination, or pneumothoraces were definitive reasons for increased bCPAP mortality. Future studies should address all of these issues. Of note, since commercially available oxygen concentrators do not titrate FiO_2_, additional oxygen delivery innovations that permit titration and high flow rates while remaining feasible and affordable for LMICs will be needed. Further, although radiographic imaging is not widely available, future research should include routine imaging to better assess the incidence of pneumothoraces among similar patients and to evaluate associations between pneumothoraces and positive end expiratory pressures. In addition, the inclusion of autopsies should be considered to more reliably attribute cause of death. Lastly, future studies should seek to more definitely address what patient populations might benefit from bCPAP including context-appropriate contraindications for its use in settings without care escalation options such as invasive mechanical ventilation.

In conclusion, while important questions remain, this trial challenges the notion that bCPAP is without risk and is ready for widespread implementation for LMIC child pneumonia care where most patients at high-risk receive care, namely, the general paediatric wards of non-tertiary district hospitals where physicians are absent. This trial also challenges the conventional wisdom that effective care in one setting always translates to another and highlights the tangible risks of implementing more complex care in resource-constrained settings. Taken together with previous bCPAP trial results and limitations, these findings generate uncertainty around bCPAP use and demand further research of bCPAP before wider implementation in similar LMIC settings.

## Data sharing

De-identified individual participant data and a data dictionary will be made available with publication with a signed data access agreement with the authors.
