# Supplementary material for: Bubble continuous positive airway pressure for children with high-risk conditions and severe pneumonia in Malawi: an open label, randomised, controlled trial
Source: Lancet Respir Med. 2019 Nov;7(11):964–74. doi: 10.1016/S2213-2600(19)30243-7 (PMC6838668; doi:10.1016/S2213-2600(19)30243-7)
Supplement: Supplementary appendix [file mmc1.pdf]

THE LANCET
Respiratory Medicine

Supplementary appendix

This appendix formed part of the original submission and has been peer reviewed. We post it as supplied by the authors.

Supplement to: McCollum ED, Mvalo T, Eckerle M, et al. Bubble continuous positive airway pressure for children with high-risk conditions and severe pneumonia in Malawi: an open label, randomised, controlled trial. *Lancet Respir Med* 2019; published online Sept 24. http://dx.doi.org/10.1016/S2213-2600(19)30243-7.

Appendix

Panel. Participant eligibility definitions

Eligibility criteria	
Inclusion criteria	• 1-59 months of age *and*<br>• WHO-defined severe pneumonia *and*<br>• One or more high-risk condition
Exclusion criteria	• Previous enrollment
Severe pneumonia	
Signs of severe pneumonia with or without a general danger sign	• Parental report or observation of cough or difficult breathing *and*<br>• ≥1 respiratory danger sign with or without a general danger sign
Signs of pneumonia with a general danger sign	• Parental report or observation of cough or difficult breathing *and*<br>• ≥1 sign of pneumonia *and*<br>• ≥1 general danger sign
High risk conditions	
Hypoxemia	• Oxyhemoglobin saturation <90% measured in room air
HIV infection	• <12 months olds with a positive HIV DNA PCR *or*<br>• ≥12 month olds with HIV antibodies
HIV exposure	• <24 month olds not meeting HIV-infection criteria with a HIV-infected mother

Severe malnutrition	• Weight-for-height three standard deviations below WHO median growth standards *or*<br>• bilateral pedal edema *or*<br>• mid-upper arm circumference <11.5 cm
Other	
Respiratory danger sign	• severe chest wall indrawing *or*<br>• head nodding *or*<br>• tracheal tugging *or*<br>• very fast breathing *or*<br>• stridor *or*<br>• apnea *or*<br>• nasal flaring *or*<br>• oxyhemoglobin saturation <90%
General danger sign	• Observed or history of convulsions within prior 24 hours *or*<br>• inability to feed *or*<br>• vomiting everything *or*<br>• Blantyre Coma Score ≤4
Pneumonia	• Fast breathing *or*<br>• Non-severe chest wall indrawing *or*<br>• oxyhemoglobin saturation 90-94% *or*<br>• crackles or wheezing on chest auscultation

Fast breathing	• 60-79 breaths/minute for 30-59 day olds<br>• 50-69 breaths/minute for 2-11 month olds<br>• 40-59 breaths/minute for 12-59 month olds
Very fast breathing	• ≥80 breaths/minute for 30-59 day olds<br>• ≥70 breaths/minute for 2-11 month olds<br>• ≥60 breaths/minute for 12-59 month olds

Supplemental Table 1. Probable or definitely related adverse death events			
	Low flow oxygen deaths N=35	bCPAP deaths N=53	P value
All, n (%)	1 (2.8%)	5 (9.4%)	0.231
Aspiration, n (%)	1 (2.8%)	4 (7.5%)	0.352
Pneumothorax, n (%)	0 (0)	1 (1.8%)	0.006

bCPAP indicates bubble continuous positive airway pressure.

Supplemental Table 2. Adverse Event Summary								
	Study #	Study arm	Adverse event grade (Grade 1 (mild), Grade 2 (mod), Grade 3 (severe), Grade 4 (life-threatening), Grade 5 (death))	Adverse event	Most recent status of event (resolved without sequelae, resolved with sequelae, death)	Status date (dd/mm/yyyy)	Seriousness criteria (NA, prolonged hospitalization, life threatening, death, persistent disability)	Relationship of adverse event to CPAP study device (related or not related)
1	U0002	Oxygen	5	Death	Death	26/06/2015	Death	Not related
2	C0007	CPAP	5	Death	Death	07/07/2015	Death	Not related
3	U0020	Oxygen	1	Eye opacities bilaterally	Resolved without sequelae	07/09/2015	Prolonged hospitalization	Not related
4	C0029	CPAP	5	Death	Death	12/09/2015	Death	Not related
5	U0032	Oxygen	5	Death	Death	26/09/2015	Death	Not related
6	C0033	CPAP	5	Death	Death	27/09/2015	Death	Related (probably)
7	C0034	CPAP	1	Nasal bleeding	Resolved without sequelae	02/10/2015	NA	Not related
8	U0037	Oxygen	3	Abdominal ileus	Resolved without sequelae	07/10/2015	Prolonged hospitalization	Not related
9	C0042	CPAP	5	Death	Death	23/10/2015	Death	Not related
10	U0049	Oxygen	5	Death	Death	23/11/2015	Death	Not related
11	C0050	CPAP	5	Death	Death	25/11/2015	Death	Not related
12	C0051	CPAP	5	Death	Death	26/11/2015	Death	Not related
13	U0052	Oxygen	5	Death	Death	28/11/2015	Death	Not related
14	U0057	Oxygen	5	Death	Death	14/12/2015	Death	Not related
15	C0061	CPAP	5	Death	Death	07/01/2016	Death	Related (possibly)
16	C0065	CPAP	5	Death	Death	11/01/2016	Death	Not related
17	C0071	CPAP	5	Death	Death	19/01/2016	Death	Not related
18	C0072	CPAP	4	Power outage	Resolved without sequelae	22/01/2016	Prolonged hospitalization	Not related
19	U0073	Oxygen	4	Power outage	Resolved without sequelae	22/01/2016	Prolonged hospitalization	Not related
20	U0074	Oxygen	5	Death	Death	21/01/2016	Death	Not related
21	C0076	CPAP	5	Death	Death	29/01/2016	Death	Not related
22	C0077	CPAP	5	Death	Death	27/01/2016	Death	Not related
23	U0085	Oxygen	5	Death	Death	02/02/2016	Death	Not related
24	C0107	CPAP	5	Death	Death	04/03/2016	Death	Not related
25	C0127	CPAP	5	Death	Death	21/03/2016	Death	Not related
26	C0137	CPAP	5	Death	Death	02/04/2016	Death	Not related
27	C0138	CPAP	5	Death	Death	02/04/2016	Death	Not related
28	U0142	Oxygen	5	Death	Death	14/04/2016	Death	Not related
29	U0151	Oxygen	5	Death	Death	28/04/2016	Death	Not related
30	C0153	CPAP	5	Death	Death	03/05/2016	Death	Not related
31	C0160	CPAP	5	Death	Death	12/05/2016	Death	Not related
32	C0172	CPAP	5	Death	Death	12/06/2016	Death	Not related

33	U0177	Oxygen	5	Death	Death	21/06/2016	Death	Not related
34	C0178	CPAP	5	Death	Death	16/06/2016	Death	Not related
35	U0180	Oxygen	5	Death	Death	21/06/2016	Death	Not related
36	U0182	Oxygen	5	Death	Death	22/06/2016	Death	Not related
37	U0187	Oxygen	5	Death	Death	25/06/2016	Death	Not related
38	U0189	Oxygen	5	Death	Death	29/06/2016	Death	Not related
39	C0197	CPAP	5	Death	Death	02/07/2016	Death	Not related
40	C0199	CPAP	5	Death	Death	05/07/2016	Death	Not related
41	U0210	Oxygen	5	Death	Death	18/07/2016	Death	Not related
42	U0211	Oxygen	5	Death	Death	18/07/2016	Death	Not related
43	C0213	CPAP	5	Death	Death	20/07/2016	Death	Not related
44	U0224	Oxygen	5	Death	Death	29/07/2016	Death	Not related
45	U0231	Oxygen	5	Death	Death	09/08/2016	Death	Not related
46	C0233	CPAP	5	Death	Death	10/08/2016	Death	Not related
47	U0238	Oxygen	5	Death	Death	12/08/2016	Death	Not related
48	C0252	CPAP	5	Death	Death	08/09/2016	Death	Not related
49	U0261	Oxygen	5	Death	Death	25/09/2016	Death	Not related
50	C0262	CPAP	1	Nasal skin breakdown	Resolved without sequelae	03/10/2016	NA	Related (definitely)
51	C0278	CPAP	1	Nasal skin breakdown	Resolved without sequelae	14/10/2016	NA	Related (definitely)
52	U0293	Oxygen	5	Death	Death	3/11/2016	Death	Not related
53	C0296	CPAP	5	Death	Death	13/11/2016	Death	Not related
54	C0299	CPAP	5	Death	Death	19/11/2016	Death	Not related
55	C0300	CPAP	2	Nasal skin breakdown	Resolved without sequelae	26/11/2016	NA	Related (definitely)
56	U0309	Oxygen	5	Death	Death	9/12/2016	Death	Not related
57	U0314	Oxygen	5	Death	Death	9/12/2016	Death	Not related
58	C0315	CPAP	1	Nasal skin breakdown	Resolved without sequelae	14/12/2016	NA	Related (definitely)
59	U0317	Oxygen	5	Death	Death	14/12/2016	Death	Not related
60	U0318	Oxygen	4	Power outage	Resolved without sequelae	6/01/2017	NA	Not related
61	C0319	CPAP	5	Death	Death	7/1/2017	Death	Not related
62	C0320	CPAP	5	Death	Death	8/1/2017	Death	Not related
63	C0325	CPAP	5	Death	Death	19/1/2017	Death	Not related
64	C0327	CPAP	5	Death	Death	25/1/2017	Death	Not related
65	C0329	CPAP	4	Power outage	Resolved without sequelae	27/01/2017	NA	Not related
66	U0330	Oxygen	4	Power outage	Resolved without sequelae	24/01/2017	NA	Not related
67	U0331	Oxygen	4	Power outage	Resolved without sequelae	24/01/2017	NA	Not related
68	U0332	Oxygen	4	Power outage	Resolved without sequelae	24/01/2017	NA	Not related
69	U0333	Oxygen	4	Power outage	Resolved without sequelae	24/01/2017	NA	Not related
70	C0334	CPAP	4	Power outage	Resolved without sequelae	30/01/2017	NA	Not related

71	C0335	CPAP	4	Power outage	Resolved without sequelae	2/02/2017	NA	Not related
72	U0336	Oxygen	5	Death	Death	3/02/2017	Death	Not related
73	C0337	CPAP	5	Death	Death	26/1/2017	Death	Not related
74	C0342	CPAP	4	Power outage	Resolved without sequelae	10/02/2017	NA	Not related
75	C0343	CPAP	4	Power outage	Resolved without sequelae	7/02/2017	NA	Not related
76	C0344	CPAP	4	Power outage	Resolved without sequelae	7/02/2017	NA	Not related
77	C0345	CPAP	4	Power outage	Resolved without sequelae	7/02/2017	NA	Not related
78	U0346	Oxygen	4	Power outage	Resolved without sequelae	7/02/2017	NA	Not related
79	C0347	CPAP	4	Power outage	Resolved without sequelae	7/02/2017	NA	Not related
80	U0348	Oxygen	4	Power outage	Resolved without sequelae	7/02/2017	NA	Not related
81	U0349	Oxygen	4	Power outage	Resolved without sequelae	7/02/2017	NA	Not related
82	U0350	Oxygen	4	Power outage	Resolved without sequelae	7/02/2017	NA	Not related
83	C0351	CPAP	4	Power outage	Resolved without sequelae	10/02/2017	NA	Not related
84	C0352	CPAP	4	Power outage	Resolved without sequelae	10/02/2017	NA	Not related
85	U0354	Oxygen	5	Death	Death	9/02/2017	Death	Not related
86	C0355	CPAP	4	Power outage	Resolved without sequelae	10/02/2017	NA	Not related
87	U0356	Oxygen	4	Power outage	Resolved without sequelae	10/02/2017	NA	Not related
88	C0357	CPAP	4	Power outage	Resolved without sequelae	10/02/2017	NA	Not related
89	C0365	CPAP	4	Power outage	Resolved without sequelae	20/02/2017	NA	Not related
90	C0366	CPAP	4	Power outage	Resolved without sequelae	20/02/2017	NA	Not related
91	U0368	Oxygen	4	Power outage	Resolved without sequelae	20/02/2017	NA	Not related
92	C0369	CPAP	4	Power outage	Resolved without sequelae	20/02/2017	NA	Not related
93	C0370	CPAP	4	Power	Resolved	20/02/2017	NA	Not related

				outage	without sequelae			
94	C0371	CPAP	4	Power outage	Resolved without sequelae	20/02/2017	NA	Not related
95	C0372	CPAP	4	Power outage	Resolved without sequelae	20/02/2017	NA	Not related
96	U0395	Oxygen	4	Power outage	Resolved without sequelae	13/03/2017	NA	Not related
97	C0396	CPAP	4	Power outage	Resolved without sequelae	14/3/2017	NA	Not related
98	C0397	CPAP	5	Death	Death	13/3/ 2017	Death	Not related
99	U0398	Oxygen	4	Power outage	Resolved without sequelae	17/03 /2017	NA	Not related
100	U0399	Oxygen	4	Power outage	Resolved without sequelae	17/03 /2017	NA	Not related
101	C0400	CPAP	4	Power outage	Resolved without sequelae	17/3/2017	NA	Not related
102	U0402	Oxygen	4	Power outage	Resolved without sequelae	17/03/2017	NA	Not related
103	C0403	CPAP	5	Death	Death	18/3/2017	Death	Not related
104	U0404	Oxygen	4	Power outage	Resolved without sequelae	17/03/2017	NA	Not related
105	C0409	CPAP	5	Death	Death	24/3/2017	Death	Not related
106	C0419	CPAP	2	Nasal skin breakdow n	Resolved without sequelae	30/3/2017	NA	Related (definitely)
107	C0425	CPAP	5	Death	Death	3/4/2017	Death	Not related
108	U0427	Oxygen	5	Death	Death	27/4/2017	Death	Related (to oxygen; probably)
109	C0455	CPAP	5	Death	Death	7/5/2017	Death	Not related
110	U0480	Oxygen	5	Death	Death	11/6/2017	Death	Not related
111	U0495	Oxygen	5	Death	Death	12/7/2017	Death	Not related
112	C0506	CPAP	5	Death	Death	20/7/2017	Death	Not related
113	C0511	CPAP	5	Death	Death	30/7/2017	Death	Not related
114	U0517	Oxygen	5	Death	Death	1/8/2017	Death	Not related
115	C0518	CPAP	5	Death	Death	1/8/2017	Death	Not related
116	C0527	CPAP	5	Death	Death	18/8/2017	Death	Not related
117	C0531	CPAP	5	Death	Death	27/8/2017	Death	Related (probably)
118	C0538	CPAP	5	Death	Death	30/8/2017	Death	Not related
119	U0545	Oxygen	5	Death	Death	20/9/2017	Death	Not related
120	C0554	CPAP	5	Death	Death	4/10/2017	Death	Not related
121	C0556	CPAP	5	Death	Death	21/10/2017	Death	Not related
122	C0562	CPAP	5	Death	Death	12/11/2017	Death	Not related
123	U0563	Oxygen	5	Death	Death	10/11/2017	Death	Not related
124	C0567	CPAP	5	Death	Death	25/11/2017	Death	Not related
125	C0568	CPAP	5	Death	Death	29/11/2017	Death	Not related
126	C0570	CPAP	5	Death	Death	5/12/2017	Death	Not related
127	U0571	Oxygen	5	Death	Death	16/12/2017	Death	Not related
128	C0573	CPAP	2	Nasal skin	Resolved	22/12/2017	NA	Related

				breakdown	without sequelae			(definitely)
129	U0575	Oxygen	5	Death	Death	22/12/2017	Death	Not related
130	C0583	CPAP	5	Death	Death	9/1/2018	Death	Not related
131	C0597	CPAP	5	Death	Death	22/01/2018	Death	Related (probably)
132	C0602	CPAP	5	Death	Death	28/01/2018	Death	Not related
133	C0607	CPAP	5	Death	Death	31/01/2018	Death	Related (probably)
134	U0618	Oxygen	5	Death	Death	16/02/2018	Death	Not related

Supplemental Table 3. Hospital Mortality by Subgroup (Intention to Treat)

Variable		Low flow oxygen	bCPAP	Relative risk	95% CI	p value	Interaction beta coefficient	Interaction standard error	Interaction p value[1]
SpO_2 <90%	Yes	20/196 (10.2%)	37/219 (16.8%)	1.65	1.00, 2.75	0.048	0.220	0.423	0.602
	No	15/127 (11.8%)	16/102 (15.7%)	1.32	0.69, 2.55	0.394			
SpO_2 <90% without HIV infection, HIV exposure, or severe acute malnutrition, n/N (%)	Yes	7/142 (4.9%)	19/144 (13.1%)	2.67	1.16, 6.16	0.021	0.755	0.485	0.120
	No	28/181 (15.4%)	34/176 (19.3%)	1.24	0.79, 1.96	0.339			
HIV-infected, n/N (%)	Yes	2/21 (9.5%)	4/15 (26.6%)	2.80	0.58, 13.36	0.197	0.647	0.824	0.432
	SpO_2 ≥90%	1/15 (6.6%)	1/9 (11.1%)	1.66	0.11, 24.3	1.000			
	SpO_2 <90%	1/6 (16.7%)	3/6 (50.0%)	3.00	0.42, 21.29	0.221			
	No	33/302 (10.9%)	49/306 (16.0%)	1.46	0.97, 2.21	0.069			
HIV-exposed n/N (%)	Yes	4/50 (8.0%)	6/49 (12.2%)	1.53	0.46, 5.09	0.488	0.006	0.650	0.993
	SpO_2 ≥90%	2/44 (4.5%)	4/35 (11.4%)	2.51	0.51, 12.2	0.397			
	SpO_2 <90%	2/6 (33.3%)	2/14 (14.2%)	0.42	0.07, 2.37	0.329			
	No	31/273 (11.3%)	47/272 (17.2%)	1.52	0.99, 2.31	0.051			
Severe acute malnutrition without HIV infection or exposure, n/N (%)	Yes	22/110 (20.0%)	24/112 (21.4%)	1.07	0.64, 1.79	0.793	-0.752	0.414	0.069
	SpO_2 ≥90%	12/68 (17.6%)	11/58 (18.9%)	1.07	0.51, 2.25	0.514			
	SpO_2 <90%	10/42 (23.8%)	13/54 (24.0%)	1.01	0.49, 2.07	0.976			
	No	13/213 (6.1%)	29/209 (13.8%)	2.27	1.21, 4.25	0.010			
Malaria rapid antigen positive, n (%)	Yes	15/89 (16.8%)	23/108 (21.2%)	1.26	0.70, 2.27	0.435	0.266	0.404	0.512
	No	20/234 (8.5%)	30/213 (14.0%)	1.64	0.96, 2.81	0.067			
Age <12 months old, n/N (%)	Yes	19/211 (9.0%)	33/215 (15.3%)	1.70	1.00, 2.90	0.049	0.255	0.409	0.533
	No	16/112 (14.2%)	20/106 (18.8%)	1.32	0.72, 2.41	0.365			

Wheeze with or without crackle, n/N (%)	Yes	3/68 (4.4%)	5/60 (8.3%)	1.88	0.47, 7.57	0.369	0.254	0.739	0.731
	No	32/255 (12.5%)	48/261 (18.3%)	1.46	0.97, 2.21	0.069			
Blantyre Coma Score <5, n/N (%)	Yes	11/20 (55.0%)	16/35 (45.7%)	0.83	0.48, 1.42	0.499	0.675	0.370	0.068
	No	24/303 (7.9%)	37/286 (12.9%)	1.63	1.00, 2.66	0.049			
Severe anemia, n/N (%)[2]	Yes	4/29 (13.7%)	7/37 (18.9%)	1.37	0.44, 4.23	0.583	0.113	0.615	0.854
	No	31/294 (10.5%)	46/284 (16.1%)	1.53	1.00, 2.35	0.048			
Diarrhea, n/N (%)	Yes	2/14 (14.2%)	2/10 (20.0%)	1.40	0.23, 8.33	0.712	0.092	0.934	0.921
	No	33/309 (10.6%)	51/311 (16.3%)	1.53	1.02, 2.31	0.040			
Vomit, n/N (%)	Yes	2/8	7/11	2.55	0.71,9.16	0.153	0.586	0.687	0.394
	No	33/315	46/310	1.42	0.93, 2.15	0.103			

bCPAP indicates bubble continuous positive airway pressure; CI, confidence interval; HIV, human immunodeficiency virus; SpO_2, peripheral arterial oxyhemoglobin saturation.

[1]Tests the null hypothesis that the odds ratio for hospital mortality from bCPAP, compared to oxygen, are not different (except as much as is expected by sampling error) between the “yes” and “no” category within a stratum.

[2]Severe anemia was a hemoglobin <6 g/dL in children without severe acute malnutrition and <4 g/dL in children with severe acute malnutrition.

Supplemental table 4. Hospital Outcomes (Per Protocol)					
Variable	Low flow oxygen N=310	bCPAP N=304	OR	95% CI	P value
Alive, n (%)	275 (88.7%)	251 (82.5%)	0.93	0.87, 0.99	0.030
Dead, n (%)	35 (11.2%)	53 (17.4%)	1.54	1.03, 2.29	0.030

bCPAP indicates bubble continuous positive airway pressure; OR, odds ratio; CI, confidence interval.

Supplemental Table 5. Hospital Treatment Failure[1] by Subgroup (Intention to Treat)					
Variable	Low flow oxygen	bCPAP	Relative risk	95% CI	P value
All, n/N (%)	53/323 (16.4%)	70/321 (21.8%)	1.32	0.96, 1.83	0.081
HIV-infected, n/N (%)	3/21 (14.2%)	5/15 (33.3%)	2.33	0.65, 8.29	0.191
HIV-exposed n/N (%)	9/50 (18.0%)	8/49 (16.3%)	0.90	0.38, 2.15	0.825
Severe acute malnutrition without HIV infection or exposure, n/N (%)	29/110 (26.3%)	31/112 (27.6%)	1.05	0.68, 1.61	0.825
SpO_2 <90% without HIV infection, HIV exposure, or severe acute malnutrition, n/N (%)	12/142 (8.4%)	26/144 (18.0%)	2.13	1.12, 4.06	0.021
Age <12 months old, n/N (%)	32/211 (15.1%)	47/215 (21.8%)	1.44	0.95, 2.16	0.078
Wheeze with or without crackle, n/N (%)	8/68 (11.7%)	9/60 (15.0%)	1.27	0.52, 3.09	0.591
Blantyre Coma Score = 5, n/N (%)	42/303 (13.8%)	54/286 (11.8%)	1.36	0.94, 1.97	0.101
Malaria rapid antigen negative, n (%)	34/234 (14.5%)	40/213 (18.7%)	1.29	0.85, 1.96	0.229
No severe anemia, n/N (%)[2]	47/294 (15.9%)	60/284 (21.1%)	1.32	0.93, 1.86	0.113
No diarrhea, n/N (%)	49/309 (15.8%)	68/311 (21.8%)	1.37	0.98, 1.92	0.058

bCPAP indicates bubble continuous positive airway pressure; CI, confidence interval; HIV, human immunodeficiency virus; SpO_2, peripheral arterial oxyhemoglobin saturation.

[1]Treatment failure at day 14 of hospitalization was defined as an axillary fever ≥38.0 Celsius or the presence of any respiratory danger sign (SpO_2 <90%, grunting, head nodding, severe chest indrawing, very fast breathing (≥70 breaths/minute if 1-11 months of age; ≥60 breaths/minute if 12-59 months of age), stridor in a calm child, nasal flaring, apnea) or continued need for low flow oxygen or bCPAP treatment.

[2]Severe anemia was a hemoglobin <6 g/dL in children without severe acute malnutrition and <4 g/dL in children with severe acute malnutrition.

Supplemental Table 6. Antibiotic Treatment Failure[1] by Subgroup (Intention to Treat)					
Variable	Low flow oxygen	bCPAP	Relative risk	95% CI	P value
All, n/N (%)	255/323 (78.9%)	229/321 (71.3%)	0.90	0.82, 0.98	0.025
HIV-infected, n/N (%)	18/21 (85.7%)	10/15 (66.6%)	0.77	0.52, 1.15	0.235
HIV-exposed n/N (%)	40/50 (80.0%)	35/49 (71.4%)	0.89	0.71, 1.11	0.323
Severe acute malnutrition without HIV infection or exposure, n/N (%)	83/110 (75.4%)	76/112 (67.8%)	0.89	0.76, 1.06	0.211
SpO_2 <90% without HIV infection, HIV exposure, or severe acute malnutrition, n/N (%)	114/142 (80.2%)	108/145 (74.4%)	0.92	0.81, 1.05	0.241
Age <12 months old, n/N (%)	168/211 (79.6%)	152/215 (70.6%)	0.88	0.79, 0.99	0.034
Wheeze with or without crackle, n/N (%)	53/68 (77.9%)	44/60 (73.3%)	0.94	0.77, 1.14	0.547
Blantyre Coma Score = 5, n/N (%)	242/303 (79.8%)	213/286 (74.4%)	0.93	0.85, 1.01	0.12
Malaria rapid antigen negative, n (%)	190/234 (81.1%)	159/213 (74.6%)	0.91	0.83, 1.01	0.098
No severe anemia, n/N (%)[2]	238/294 (80.9%)	210/284 (73.9%)	0.91	0.83, 0.99	0.045
No diarrhea, n/N (%)	244/309 (78.9%)	220/311 (70.7%)	0.89	0.81, 0.98	0.019

bCPAP indicates bubble continuous positive airway pressure; CI, confidence interval; HIV, human immunodeficiency virus; SpO_2, peripheral arterial oxyhemoglobin saturation.

[1]Antibiotic treatment failure was defined for oxygen and bCPAP group patients as having any of the following by day 6 of hospitalization:

- Fever $\geq$38° Celcius *and* low flow oxygen supplementation or bCPAP treatment *or*
- Fever $\geq$38° Celcius and persistent respiratory danger signs (oxygen saturation <90%, grunting, head nodding, severe chest indrawing, or very fast breathing ($\geq$70 breaths/minute if 1-11 months; $\geq$60 breaths/minute if 12-59 months of age) or stridor in a calm child or apnea)
- Fever $\geq$38° Celcius and persistent general danger signs (inability to drink, lethargy or unconscious, convulsions)
- New respiratory danger sign (new SpO_2 <90%, new grunting, head nodding, severe chest indrawing, or very fast breathing ($\geq$70 breaths/minute if 1-11 months; $\geq$60 breaths/minute if 12-59 months of age), stridor in a calm child, nasal flaring, or apnea) *or*
- New general danger sign (inability to drink, lethargy or unconscious, convulsions) *or*
- Death

[2]Severe anemia was a hemoglobin <6 g/dL in children without severe acute malnutrition and <4 g/dL in children with severe acute malnutrition.

Supplemental Table 7. 30-day post discharge mortality among children discharged from hospital alive					
Variable	Low flow oxygen[1] N=147	bCPAP[2] N=155	Relative risk	95% CI	P value
All, n (%)	2 (1.3%)	3 (1.9%)	1.42	0.24, 8.39	0.696

bCPAP indicates bubble continuous positive airway pressure; CI, confidence interval.
[1]48 oxygen children died or were lost-to-follow-up while hospitalized and 128 oxygen children were lost-to-follow-up after hospital discharge.
[2]70 bCPAP children died or were lost-to-follow-up while hospitalized and 96 bCPAP children were lost-to-follow-up after hospital discharge.

Supplemental Table 8. Comparison of baseline characteristics by hospital survival (Post hoc analysis intention to treat)			
Variable	Died<br><br>N=88	Survived<br><br>N=556	P value
Demographic and anthropomorphic characteristics			
Age in months, median (IQR)	10.0 (4.9, 17.4)	7.4 (3.0, 14.7)	0.03
Female, n (%)	45 (51.1%)	300 (54.0%)	0.62
Weight in kg, mean (SD)	6.8 (3.1)	7.2 (2.8)	0.19
MUAC, mean (SD)	11.9 (2.0)	12.9 (1.8)	<0.01
High risk condition			
HIV-infected, n (%)	6 (6.8%)	30 (5.4%)	0.59
HIV-exposed, n (%)	10 (11.4%)	89 (16.0%)	0.26
Severe acute malnutrition without HIV infection or exposure, n (%)	46 (52.3%)	176 (31.7%)	<0.01
SpO_2 <90% without HIV infection, HIV exposure, or severe acute malnutrition	26 (29.5%)	261 (46.9%)	<0.01
Findings at presentation			
Axillary temperature in °C, mean (SD)[1]	37.6 (1.4)	37.4 (0.9)	0.06
Axillary temperature ≥38.0 °C, n (%)[1]	37 (42.0%)	135 (24.4%)	<0.01
Pulse rate in beats/minute, mean (SD)	155.3 (32.8)	165.5 (22.0)	<0.01
Respiratory rate in breaths/minute, mean (SD)	58.4 (16.2)	62.5 (14.9)	0.02
SpO_2, median (IQR)	86.0 (76.5, 94.0)	88.0 (83.5, 94.0)	0.05
SpO_2 93-100%, n (%)	28 (31.8%)	163 (29.3%)	0.63
SpO_2 90-92%, n (%)	3 (3.4%)	35 (6.3%)	0.29
SpO_2 <90% n, (%)	57 (64.8%)	358 (64.4%)	0.94
Any respiratory danger sign, n (%)	87 (98.9%)	551 (99.1%)	0.83
Severe lower chest wall indrawing, n (%)	75 (85.2%)	500 (89.9%)	0.19
Head nodding or tracheal tugging, n (%)	21 (23.9%)	156 (28.1%)	0.41
Grunting, n (%)	38 (43.2%)	116 (20.9%)	<0.01
Very fast breathing for age, n (%)[2]	24 (27.3%)	187 (33.6%)	0.24
Stridor while calm, n (%)	1 (1.1%)	12 (2.2%)	0.53
Apnea, n (%)	12 (13.6%)	25 (4.5%)	<0.01
Nasal flaring, n (%)	66 (75.0%)	405 (72.8%)	0.67
Any general danger sign, n (%)	45 (51.1%)	75 (13.5%)	<0.01
Convulsions, n (%)	18 (20.5%)	43 (7.7%)	<0.01
Inability to feed, n (%)	34 (38.6%)	44 (7.9%)	<0.01
Vomiting everything, n (%)	9 (10.2%)	10 (1.8%)	<0.01

Blantyre Coma Score ≤4, n (%)	27 (30.7%)	28 (5.0%)	<0.01
Crackle without wheeze, n (%)	36 (40.9%)	275 (49.5%)	0.14
Wheeze with or without crackle, n (%)	8 (9.1%)	120 (21.6%)	<0.01
Severe anemia, n (%)[1,3]	11 (12.5%)	54 (9.7%)	0.42
Diarrhea, n (%)	4 (4.5%)	20 (3.6%)	0.66
Dehydration, n (%)	19 (21.6%)	14 (2.5%)	<0.01
Laboratory assessments			
Hemoglobin in g/dL, mean (SD)[1]	8.9 (2.7)	9.6 (2.6)	0.03
Malaria rapid antigen positive, n (%)	38 (43.2%)	159 (28.6%)	<0.01

bCPAP indicates bubble continuous positive airway pressure; IQR, interquartile range; SD, standard deviation; MUAC, mid-upper arm circumference; HIV, human immunodeficiency virus; SpO_2, peripheral arterial oxyhemoglobin saturation.

[1]322 and 320 participants in the low-flow oxygen group and bCPAP group had an axillary temperature measured and 320 in the bCPAP group had a hemoglobin collected.

[2]Very fast breathing for age defined as ≥80 breaths/minute for 30 to 59 day olds, ≥70 breaths/minute for 2-11 month olds, and ≥60 breaths/minute for 12-59 month olds

[3]Severe anemia was a hemoglobin <6 g/dL in children without severe acute malnutrition and <4 g/dL in children with severe acute malnutrition.

Supplemental Table 9. Comparison of implementation fidelity indicators by hospital survival (Post hoc analysis intention to treat)			
Variable	Died<br><br>N=88	Survived<br><br>N=556	P value
Nasogastric tube feeding			
Eligible for nasogastric tube, n (%)	84 (95.5%)	535 (96.2%)	0.765
Nasogastric tube inserted, n (%)	31 (36.9%)	47 (8.8%)	<0.001
Respiratory support			
Length of respiratory support in days, mean (SD)	2.8 (1.7)	4.3 (2.3)	<0.001

SD indicates standard deviation.

Serious Adverse Event narratives:

1)

Patient C0033 was a 11 month old female enrolled into the CPAP IMPACT study with severe clinical pneumonia, hypoxemia, and severe malnutrition on 25/9/2015. At enrollment the child was breathing at 68 breaths/minute which did not meet criteria for very fast breathing for age, had severe chest indrawing, crackles on chest auscultation, persistent nasal flaring, grunting, and an oxygen saturation of 68% breathing room air. The patient a heart rate of 145 beats per minute and had no general danger signs. The patient's weight was 4.6 kg and mid-upper arm circumference was 9.8 cm. HIV status at enrollment was HIV-uninfected. Patient had no diarrhea and was assessed to have no signs of dehydration. Axillary temperature was 38.2 degrees celcius. The patient's hemoglobin was 10.3 g/dl and the rapid malaria test was positive.

The patient was initiated on 8cm H20 CPAP and the parenteral antibiotics benzylpenicillin and gentamicin and artesunate. The child's oxygenation improved to 100% and the grunting improved but the other respiratory signs persisted. NG tube was not inserted as the caregiver refused.

The child was slowly improving and CPAP was weaned to 5cm H20 pressure, however, on hospital day 3 (27/9/2015) the child suddenly died. On 27/9/2015 the clinical officer was called to the patient by the caregiver for acute cardiorespiratory arrest. The caregiver was feeding the child formula (F75) and the child began coughing and then subsequently became apneic. Urgent suctioning was performed as well as cardiopulmonary resuscitation, which was unsuccessful.

This adverse event was graded as 5 and considered possible related to the study intervention of CPAP. There was no evidence of acute pneumothorax by chest auscultation prior to deterioration, however, there was a temporally related episode of choking with feeds while on CPAP. It is possible that the CPAP pressure, in combination with swallow dysfunction due to the severe respiratory distress, promoted aspiration of the milk feed. Notably the caregiver refused NG tube placement despite extensive counseling. It is also possible that this event was more primarily due to the swallow dysfunction from the respiratory distress and not the CPAP pressure delivery given the child successfully fed on higher CPAP pressures during this hospitalization.

2)

Patient C0061 was a 6 month old female enrolled into the CPAP IMPACT study with severe clinical pneumonia, hypoxemia and severe malnutrition on 5/1/2016. At enrollment the child was breathing at 54 breaths/minute, had chest indrawing, persistent nasal flaring, and an oxygen saturation of 88% breathing room air. The patient a heart rate of 156 beats per minute and had no general danger signs. The patient's weight was 5.6 kg and mid-upper arm circumference was 11.4 cm. HIV status at enrollment was HIV-uninfected. Patient had no diarrhea but was assessed to have some signs of dehydration. Axillary temperature was 37.8 degrees celcius. The patient's hemoglobin was 8.3 g/dl and the rapid malaria test was positive.

The patient was initiated on 8 cm H20 CPAP and the parenteral antibiotics benzylpenicillin and gentamicin and artesunate. The child's oxygenation improved to 100% but the other respiratory signs persisted. NG tube was not inserted.

On hospital day 3 (7/1/16) the child was switched to ceftriaxone due to persistent fevers and respiratory distress and worsening of BCS to 1/5. A bolus of normal saline 10cc/kg was given for severe dehydration. Mental status did not improve. The child vomited around this time and was successfully suctioned. Low flow oxygen was initiated temporarily until child stabilized and then was restarted on CPAP. 2 hours later at 16:20 the study nurse urgently evaluated the child due to gasping respirations which progressed into cardiopulmonary arrest. Resuscitation was attempted unsuccessfully.

This adverse event was graded as 5 and considered possibly related to the study intervention of CPAP given the child was critically ill on admission but was noted to have a vomiting episode about 2 hours prior to death. However, the child was suctioned after emesis and stabilized, and was overall deteriorating despite maximum therapies prior to the emesis event. It does remain possible that there was some aspiration that occurred prior to further deterioration. In addition, there was no evidence of acute pneumothorax by chest auscultation prior to deterioration.

3)

Patient C0262 was a 1.3 month old male enrolled into the CPAP IMPACT study with severe clinical pneumonia and severe malnutrition on 23/9/2016. At enrollment the child was breathing at 75 breaths/minute which did not meet criteria for very fast breathing for age (but did for fast breathing), had severe chest indrawing, persistent nasal flaring, no head nodding, no grunting, no wheezing, no apnea, no stridor while calm, and an oxygen saturation of 89% breathing room air. The patient a heart rate of 185 beats per minute and had no general danger signs. The patient's weight was 3.8 kg and mid-upper arm circumference was 11.3 cm. HIV status at enrollment was HIV-uninfected. Patient did not have diarrhea and was assessed to have no signs of dehydration. Axillary temperature was 36.5 degrees celcius. The patient's hemoglobin was 14.3 g/dl and the rapid malaria test was negative.

The patient was initiated on CPAP by nasal mask at 7 cm H20 and the parenteral antibiotics benzylpenicillin and gentamicin. The child's oxygenation improved to 100% but the other respiratory signs persisted. NG tube was not inserted.

On hospital day 6 the child was switched to ceftriaxone due to persistent CPAP requirement. The child was weaned slowly from CPAP and was weaned to oxygen on 28/9/2016, and subsequently to room air on 30/9/2016.

On hospital day 5 the child was observed to have skin discoloration around the nose, which resolved after the child was weaned off of CPAP. The adverse event resolved on hospital day 10 (3/10/2016).

This adverse event was graded as 1 and considered related to the study intervention of CPAP.

4)

Patient C0278 was a 1.6 month old female enrolled into the CPAP IMPACT study with severe clinical pneumonia and severe acute malnutrition on 9/10/2016. At enrollment the child was breathing at 52 breaths/minute which did not meet criteria for fast breathing for age, had severe chest indrawing, persistent nasal flaring, no head nodding, no grunting, no wheezing, no apnea, no stridor while calm, and an oxygen saturation of 86% breathing room air. The patient a heart rate of 188 beats per minute and had no observed general danger signs. The patient's weight was 4.0 kg and mid-upper arm circumference was 11.4cm. HIV status at enrollment was HIV-uninfected. Patient did not have diarrhea and was assessed to not have signs of dehydration. Axillary temperature was 38.2 degrees celcius. The patient's hemoglobin was 13.1 g/dl and the rapid malaria test was negative.

The patient was initiated on CPAP at 7 cm H20 by nasal mask and the parenteral antibiotics benzylpenicillin and gentamicin. The child's oxygenation improved to 97% but the other respiratory signs persisted. NG tube was not inserted. At 60 minute reassessment the child showed minimal improvement.

On hospital day 5 the child was observed to have skin breakdown on the right side of the ridge of the nose where the CPAP mask was interfacing with the skin. The mask size was changed from medium to small and the skin healed nicely prior to hospital discharge. The adverse event resolved on hospital day 7 (16/10/2016).

This adverse event was graded as 1 and considered related to the study intervention of CPAP.

5)

Patient C-0300 was 15 months old male child enrolled into the CPAP IMPACT study with severe clinical Pneumonia and severe hypoxemia on 17/11/2016. At enrollment the child was breathing at 68 b/min which met the criteria for very fast breathing for age, had severe chest indrawing, and an oxygen saturation of 37% breathing room air. He had a heart rate of 164 b/min and no general danger sign. Patients weight was 7.3 kg, Mid Upper Arm Circumference was 11.6 cm. HIV status at enrollment was negative. Patient had no diarrhoea and no signs of dehydration. Axillary temperature was 38.1 degrees celcius. Haemoglobin was 11.2g/dl and the rapid malaria test was negative

The patient was initiated on High CPAP by nasal mask at 8cm and parental antibiotics - Benzyl penicillin and Gentamycin. At 60 minute reassessment the child's oxygenation improved to 100% and remained with one respiratory sign - severe chest indrawing. No NGT was inserted and feeds were continues orally.

On hospital day 5 (22/11/2016) child was initiated on Ceftriaxone due to meeting treatment failure criteria based upon the presence of two respiratory danger signs and the inability to wean from CPAP support. A routine chest radiograph was obtained and did not show pneumothorax nor cardiomegaly.

On hospital day 8 (25/11/2016) the child was observed to be having nasal skin erosion around the nose due to the CPAP mask. As intervention to correct the problem to avoid further damage to the nose the mask was changed and CPAP prongs were inserted.

This adverse event was graded as 2 and considered related to the study intervention of CPAP.

On hospital day 9 (26/11/2016) the child’s caregivers withdrew the patient from the study and left the hospital against medical advice.

6)

Patient C-0315 was a three month old male enrolled into CPAP IMPACT study with severe clinical pneumonia and severe hypoxemia, severe malnutrition and suspected Down's Syndrome (abnormal facies) on 8th December 2016. At enrollment the child was breathing at 44 breaths per minute, had severe chest in-drawing, persistent nasal flaring, and an oxygen saturation of 70% breathing room air. The patient had a heart rate of 148 beats/ minute and had no general danger signs. Patient’s weight was 5.0kg and mid upper arm circumference was 11.1cm. HIV status was negative. Patient had no diarrhea and was assessed to have no signs of dehydration. Axillary temperature was 36.6. Patients hemoglobin was 11.1g/dl and rapid malaria test was negative

Patient was initiated on high CPAP by nasal mask at 8cm H20 and parenteral antibiotics Benzyl Penicillin and Gentamycin. The child's oxygenation improved to 100% but other respiratory danger signs persisted. Feeds were held initially but NG was not inserted.

On hospital day 2 (9/Dec/2016) the child was assessed in the afternoon round and had no any respiratory danger signs and was therefore weaned to 5cm H20 on CPAP per protocol.

On day 3 (10/Dec/2016) during the morning evaluation the child had worsened and developed severe chest indrawing and persistent nasal flaring as well as an elevated temperature of 38.9 degrees Celsius. Therefore CPAP increased back to 8cm H20 pressure and was switched to ceftriaxone.

On hospital day 4 (11/Dec/2016) the child was observed to have macerated skin on the lateral aspects of the nose where the CPAP mask was placed. Skin care was initiated and the nasal Mask was changed to a smaller nasal mask to avoid this skin area and permit healing so as to prevent further nasal damage.

This adverse event was graded as 2 and considered related to the study intervention of CPAP.

The macerated skin improved over the next 24-48 hours. On hospital day 6 (14/Dec/2016) the caregiver withdrew the child from the study and left the hospital against medical advice, citing the need to care for other children in the home.

7)

Patient ID C-0419 was a 1 month old male child enrolled into the CPAP IMPACT study with severe clinical pneumonia and severe hypoxemia on March 25, 2017. At enrollment the child was breathing at 80 breaths/minute which met criteria for very fast breathing for age, had severe chest indrawings, and persistent nasal flaring, and an oxygen saturation of 86% breathing room air. The patient had a heart rate of 182 beats per minute and no general danger signs. The patient's weight was 4.6 kg and mid-upper arm circumference was 12.3 cm. HIV status at enrollment was negative. Patient had no diarrhea and was assessed to have no signs of dehydration. Axillary temperature was 38.3 degrees celcius. The patient's hemoglobin was 11.1 g/dl and the rapid malaria test was negative.

The patient was initiated on HIGH CPAP by nasal mask at 7 cmH20 and the first dose of benzyl penicillin and gentamycin were given. The child's oxygenation improved to 100% but the other respiratory signs persisted. A NG tube was not inserted although feeds were held until the child stabilized. At the 60 minute reassessment the child improved.

On hospital day 1 at 06:00hrs, 08:00hrs and 12:00hrs the child developed new respiratory danger sign of head nodding respectively but was already on high cpap, so no further escalation was made.

On hospital day 2 at 8:00hrs assessment child had only one respiratory danger sign, chest indrawing, and was weaned to low cpap according to study protocol. The 60 minutes recheck was done and the child had not developed any additional danger sign and vital signs were stable. On this same day 2 at 14:00hrs assessment the child progressed to develop head nodding again, along with chest indrawing, therefore, the child was escalated back to high cpap.

On hospital day 4 the child was eligible to be weaned to low cpap according to protocol at 14:00hrs because the staff found the child to have only chest indrawing. On hospital day 5 the child completed benzyl penicillin and gentamycin but remained with 1 respiratory danger sign of severe chest indrawing, therefore the child met criteria to switch to ceftriaxone.

On day 6 at 08:00 the child was observed to have an adverse event of bruising and mild discoloration around the nose when the child was weaned to 0.5 litres of oxygen from low cpap. Silver cream was applied on the lacerations around the nose and on the day of discharge the lacerations were fully healed.

This adverse event was graded as 2 and considered related to the study intervention of CPAP.

8)

Patient U-0427 was a 51 months old female child enrolled into the CPAP IMPACT study with severe clinical pneumonia and hypoxemia on April 4, 2017. At enrollment the child had severe chest indrawing, persistent nasal flaring, head nodding, and an oxygen saturation of 77% breathing room air. The patient had a heart rate of 144 beats per minute and with the general danger signs of a low Blantyre Coma Score of 3/5, reported convulsions, and was unable to feed. The patient's weight was 14.1 kg and mid-upper arm circumference was 15.3 cm. HIV status at enrollment was negative. Patient had some diarrhea and was assessed to have some signs of dehydration. Axillary temperature was 38.9 degrees celcius. The patient's hemoglobin was 6.2 g/dl and the rapid malaria test was positive.

The patient was initiated on low flow oxygen at 2LPM and the parenteral antibiotic ceftriaxone and artesunate. At the 60 minute reassessment, the child's oxygenation slightly improved to 82% but the other respiratory signs persisted. A NG tube was inserted and feeds were held.

On the day of admission at 14:00 hours the child was re-reviewed. The child had an oxygen saturation of 85% despite 2LPM Oxygen, heart rate 183 beats/minute, respirations at 66 breaths/minute and a temperature of 37.4 degrees celcius. The respiratory signs of nasal flaring and very fast breathing for age were present. The child was still unable to feed with a Blantyre Coma Score of 3/5 on NGT.

At 15:50 the child was urgently reviewed when observed to be vomiting from mouth and nose. Suctioning was done and gasping respirations were observed. Resuscitation with Bag Mask valve was done but child went into cardiac and respiratory arrest and death was confirmed after 5 minutes.

This adverse event was graded as 5 and considered probably related to oxygen administration but unrelated to the study intervention of CPAP given the child did not receive CPAP.

9)

Patient study id C-0531 was a 2 month old male enrolled into the CPAP IMPACT study with severe clinical pneumonia 25th of August 2017 at 13:00hrs. At enrollment the child was breathing at respiratory rate of 82 breaths/minute which met criteria for very fast breathing for age, had severe chest indrawing, persistent nasal flaring, and an oxygen saturation of 76% breathing room air. The patient a heart rate of 144 beats per minute and had no the general danger signs. The patient's weight was 4.0 kg and mid-upper arm circumference was 11.8 cm. HIV status at enrollment was negative. Patient had no diarrhea and was assessed to have no signs of dehydration. Axillary temperature was 36.8 degrees celcius. The patient's hemoglobin was 10.5 g/dl and the rapid malaria test was negative.

The patient was initiated on high CPAP by nasal mask at 8cm H2O pressure and the parenteral antibiotics benzylpenicillin and gentamicin. The child's oxygenation improved to 97% but the other respiratory signs persisted. NG tube was not inserted, although feeding was held until the child had stabilized. At the 60 minute reassessment the child was noted to have generally improved.

On the same day of enrollment at 18:00hrs the child developed an additional respiratory danger sign of head nodding. The mother was counseled about the recommendation for a NGT but she refused.

On day 2 (August 26, 2017) at 08:00hrs the child was assessed and observed with severe chest indrawing and nasal flaring. At 18:00hrs the child was reassessed and found to have redeveloped very fast breathing despite CPAP . At midnight, now August 27, 2017, the child was reassessed and found to have progressed to develop head nodding despite CPAP, in addition to severe chest indrawing and persistent nasal flaring.

On day 3 (06:00hrs, August 27, 2017) the child was assessed and had very fast breathing of 82 breaths per minute, severe chest indrawing, head nodding, grunting and persistent nasal flaring and had a reduced coma score of 4/5 while on high CPAP (8cm H20 pressure). Mum was recounseled about the need for a NGT and this time she accepted. NG tube was inserted, positioning was confirmed by auscultation, and feedings were switched to NG tube only. Nursing was called at 06:20am and observed the child vomiting greenish emesis from both the mouth and nostrils after the mother had just fed the child. The child became apneic. Suctioning followed by bag and mask ventilation and chest compressions were done. The child was pronounced dead at 06:30am.

This adverse event was graded as 5 and considered likely related to the study intervention of CPAP since the child had a temporally related episode of vomiting suggesting an aspiration event, possibly secondary to gastric distension while on CPAP. There was no evidence of acute pneumothorax by chest auscultation prior to deterioration.

10)

Patient C-0573 was a 47 month old female enrolled into the CPAP IMPACT study with severe clinical pneumonia and malnutrition on the 18th December 2017. At enrollment the child was breathing at 52 breaths/minute, had severe chest indrawings, persistent nasal flaring, and an oxygen saturation of 97% breathing room air. The patient had a heart rate of 153 beats/minute and a Blantyre coma score of 3/5, had reported convulsions, and was not able to feed. The patient's weight was 8.9 kg and mid-upper arm circumference was 11.5cm. HIV status at enrollment was negative. The child had no diarrhea and was assessed to have no signs of dehydration. Axillary temperature was 39.2 degrees Celsius. The patient's hemoglobin was 3.7 g/dl and the rapid malaria test was positive.

The patient was initiated on High CPAP by nasal mask at 8 cm H20 and 8 LPM flow and the parenteral antibiotic of ceftriaxone. At the 60 minutes recheck the child's oxygenation had improved to 100% but severe chest indrawings and very fast breathing persisted. A NG tube was inserted and feeds were held initially.

On hospital day 4, during rounds, it was observed that the child has mild skin breakdown around the nose. The mother was counseled about the problem and the nasal interface was changed to prongs, and petroleum jelly was applied to the skin with routine frequency during the remainder of the hospitalization. Appropriate nursing care was given.

This adverse event was graded as 2 and considered related to the study intervention of CPAP.

11)

Patient study ID C-0597 was a 9 month old female enrolled into the CPAP IMPACT study with severe clinical pneumonia and severe hypoxemia on 20th January 2018. At enrollment the child was breathing at respiratory rate of 81 breaths/minute which met criteria for very fast breathing for age, had severe chest indrawing, persistent nasal flaring, and an oxygen saturation of 88% breathing room air. The patient a heart rate of 169 beats per minute and had no general danger signs. The patient's weight was 6.7 kg and mid-upper arm circumference was 12.7cm. HIV status at enrollment was negative. The patient had no diarrhea and was assessed to have no signs of dehydration. Axillary temperature was 37.9 degrees celcius. The patient's hemoglobin was 9.6g/dl and the rapid malaria test was negative.

The patient was initiated on high CPAP by nasal mask at 8cm H2O pressure and the parenteral antibiotics benzyl penicillin and gentamicin. Nebulizer was done for wheeze. The child's oxygenation improved to 100% but the other respiratory signs persisted. A NG tube was not inserted but feeds were held initially.

On hospital day 1 at 21:45 hours, the child’s respiratory distress appeared worse and an urgent evaluation was done. The respiratory rate was 82 breaths per minute, oxygen saturation was 100% and heart rate was 179 beats per minute. A NGT was inserted. The child continued on high CPAP. On auscultation the child had reduced air entry into the left lung base. Trachea was midline. The hospital radiology services were closed for the night and therefore the plan was to stabilize the child until imaging could be obtained.

Approximately 6 hours later on hospital day 2 (22 January 2018) at 04:15am the child had respiratory arrest and resuscitation was attempted but to no avail. The child was pronounced dead at 04:45am.

This adverse event was graded as 5 and considered probably related to the study intervention of CPAP since there was possible evidence of acute pneumothorax by chest auscultation around the time of clinical deterioration.

12)

Patient C-0607 was 3 months old female enrolled into the CPAP IMPACT study with severe clinical pneumonia and severe malnutrition on 30/01/2018. At enrollment the child's respirations were 61 breaths/minute and the child had severe chest indrawing and an oxygen saturation of 98% breathing room air. The child had no general danger signs. The patient weighed 2.5 kg with a Mid-Upper Arm Circumference was 9.0 cm. HIV status was Negative. The patient had no diarrhea, was assessed to have no signs of dehydration. The child was hypothermic with a body temperature of 33.9 C. Hemoglobin was 10.9g/dl and the malaria rapid test was positive.

The child was initiated on high CPAP by nasal mask at 8 cm H20 / 8 LPM and the parenteral drugs benzyl penicillin, gentamycin and antimalarial artesunate were given.

At 60 minutes the was observed to have two respiratory danger signs of severe chest indrawing and nasal flaring. Good hydration status recorded with no diarrhea. No NG tube was initiated and feeding was held initially.

On hospital Day 0, 30/01/18 at 2:00 pm the child appeared stable and was permitted to feed orally at this point under close observation.

Hospital Day 1 (31/01/18) at 10:33 am, an urgent review was done about 2 hours after morning rounds. The child's Blantyre Coma Score had decreased to 2/5, respirations were at 9 breaths /min, oxygen saturation and the heart rate were both unrecordable. Vomit was present in the child's mouth and on the bed. The patient was apneic. Airway suctioning done urgently, CPR commenced while bagging with oxygenated mask for 15 minutes. No spontaneous respirations or heart rate regained despite resuscitation. Pupils fixed, dilated and unresponsive to light. Death confirmed to guardians.

This adverse event was graded as 5 and considered probably related to the study intervention of CPAP since as there was a temporally related episode of vomiting to suggest an aspiration event, possibly secondary to gastric distension while on CPAP.

Consort Checklist

CONSORT 2010 checklist of information to include when reporting a randomised trial*

Section/Topic	Item No	Checklist item	Reported on page No
**Title and abstract**			
	1a	Identification as a randomised trial in the title	1
	1b	Structured summary of trial design, methods, results, and conclusions (for specific guidance see CONSORT for abstracts)	4-5
**Introduction**			
Background and objectives	2a	Scientific background and explanation of rationale	10-12
	2b	Specific objectives or hypotheses	12
**Methods**			
Trial design	3a	Description of trial design (such as parallel, factorial) including allocation ratio	12
	3b	Important changes to methods after trial commencement (such as eligibility criteria), with reasons	12
Participants	4a	Eligibility criteria for participants	13
	4b	Settings and locations where the data were collected	13
Interventions	5	The interventions for each group with sufficient details to allow replication, including how and when they were actually administered	14-19
Outcomes	6a	Completely defined pre-specified primary and secondary outcome measures, including how and when they were assessed	15
	6b	Any changes to trial outcomes after the trial commenced, with reasons	N/A
Sample size	7a	How sample size was determined	19
	7b	When applicable, explanation of any interim analyses and stopping guidelines	19-20
Randomisation:			
Sequence generation	8a	Method used to generate the random allocation sequence	15
	8b	Type of randomisation; details of any restriction (such as blocking and block size)	15
Allocation concealment mechanism	9	Mechanism used to implement the random allocation sequence (such as sequentially numbered containers), describing any steps taken to conceal the sequence until interventions were assigned	15
Implementation	10	Who generated the random allocation sequence, who enrolled participants, and who assigned participants to interventions	15
Blinding	11a	If done, who was blinded after assignment to interventions (for example, participants, care providers, those assessing outcomes) and how	N/A
	11b	If relevant, description of the similarity of interventions	17-19
Statistical methods	12a	Statistical methods used to compare groups for primary and secondary outcomes	19-20
	12b	Methods for additional analyses, such as subgroup analyses and adjusted analyses	19
**Results**			
Participant flow (a diagram is strongly recommended)	13a	For each group, the numbers of participants who were randomly assigned, received intended treatment, and were analysed for the primary outcome	21, Figure 1
	13b	For each group, losses and exclusions after randomisation, together with reasons	Figure 1
Recruitment	14a	Dates defining the periods of recruitment and follow-up	21
	14b	Why the trial ended or was stopped	13
Baseline data	15	A table showing baseline demographic and clinical characteristics for each group	41-44
Numbers analysed	16	For each group, number of participants (denominator) included in each analysis and whether the analysis was by original assigned groups	21-24
Outcomes and estimation	17a	For each primary and secondary outcome, results for each group, and the estimated effect size and its precision (such as 95% confidence interval)	21-24
	17b	For binary outcomes, presentation of both absolute and relative effect sizes is recommended	21
Ancillary analyses	18	Results of any other analyses performed, including subgroup analyses and adjusted analyses, distinguishing pre-specified from exploratory	21-24
Harms	19	All important harms or unintended effects in each group (for specific guidance see CONSORT for harms)	21-22
**Discussion**			
Limitations	20	Trial limitations, addressing sources of potential bias, imprecision, and, if relevant, multiplicity of analyses	25-34
Generalisability	21	Generalisability (external validity, applicability) of the trial findings	25-29
Interpretation	22	Interpretation consistent with results, balancing benefits and harms, and considering other relevant evidence	25-26
**Other information**			
Registration	23	Registration number and name of trial registry	13
Protocol	24	Where the full trial protocol can be accessed, if available	13
Funding	25	Sources of funding and other support (such as supply of drugs), role of funders	20-21

*We strongly recommend reading this statement in conjunction with the CONSORT 2010 Explanation and Elaboration for important clarifications on all the items. If relevant, we also recommend reading CONSORT extensions for cluster randomised trials, non-inferiority and equivalence trials, non-pharmacological treatments, herbal interventions, and pragmatic trials. Additional extensions are forthcoming: for those and for up to date references relevant to this checklist, see www.consort-statement.org.

<u>Effectiveness of bubble CPAP in reducing childhood pneumonia mortality in Malawi</u>

Eudowood Division of Pediatric Respiratory Sciences
Department of Pediatrics
Johns Hopkins School of Medicine

Version 1.5
December 8, 2017

INVESTIGATORS

Principal investigator:
Eric D. McCollum, MD
Assistant Professor of Pediatrics
Eudowood Pediatric Respiratory Sciences
Johns Hopkins School of Medicine
200 North Wolfe Street

Co-investigators:
Mina Hosseinipour, MD, MPH
Professor of Medicine
Department of Internal Medicine
Division of Infectious Diseases
University of North Carolina- Chapel Hill

Tisungane Mvalo, MD
Assistant Professor of Medicine
Department of Pediatrics
University of North Carolina- Chapel Hill

Michelle Eckerle, MD, MPH
Assistant Professor
Department of Pediatrics
Division of Emergency Medicine
Cincinnati Children's Hospital Medical Center
Cincinnati, OH 45229 USA

Dr. Andrew Smith, MD
Assistant Professor
Department of Pediatrics
Division of Pediatric Intensive Care
Primary Children's Hospital
Salt Lake City, UT 84113

Brian Weir, PhD
Assistant Scientist
Department of Health, Behavior and Society
Johns Hopkins Bloomberg School of Public Health
624 N Broadway Room 261
Baltimore, MD 21287 USA

Dhananjay Madhukar Vaidya, M.B.B.S., M.P.H., Ph.D.
Associate Professor of Medicine
General Internal Medicine Department
Johns Hopkins School of Medicine
624 N Broadway
Baltimore, MD 21287 USA

PARTNERS

**Primary:**	Johns Hopkins School of Medicine<br>Department of Pediatrics<br>Eudowood Division of Pediatric Respiratory Sciences<br>The David Rubenstein Child Health Building<br>200 N. Wolfe Street, 3rd floor<br>Baltimore, MD 21287
**Clinical Trial Sites:**	Salima District Hospital<br>Salima<br>Malawi
**Study Operations:**	UNC Project, Lilongwe, Malawi<br>Kamuzu Central Hospital<br>Private Bag A104<br>Lilongwe, Malawi
**Local Collaborators:**	Malawi Ministry of Health (MOH)<br>UNC Project<br>Salima District Hospital<br>Cincinnati Children's Hospital Medical Center<br>Primary Children's Hospital, Salt Lake City, Utah
**Funding Agency:**	International AIDS Society<br>Bill and Melinda Gates Foundation

Executive Summary

Pneumonia mortality rates in African countries like Malawi are high and increased further in children – exposed or infected with human immunodeficiency virus (HIV) as well as those that are severely malnourished or severely hypoxemic. Treatment innovations are needed. Bubble continuous positive airway pressure (bCPAP) improves oxygenation and ventilation and is a simple, relatively inexpensive adaptation of conventional continuous positive airway pressure potentially suitable for low-resource settings. bCPAP has been demonstrated to improve outcomes in neonates less than 1 month of age. Recently, a limited number of hospitals are using bCPAP to escalate pneumonia care for older African children failing standard treatment with antibiotics and oxygen. Supportive evidence for this approach is observational only. Quality randomized studies comparing bCPAP versus a standard-of-care control group that includes low-flow oxygen therapy and using a primary endpoint of mortality are not available in low-resource settings including high prevalence HIV countries like Malawi. Demonstrating a mortality benefit with bCPAP is needed to support further investment and scale up of bCPAP in the care of older high risk Malawian children 1-59 months of age with World Health Organization (WHO) severe pneumonia complicated by HIV and/or malnutrition or severe hypoxemia.

With the **full support of the Malawi Ministry of Health** and in collaboration with external experts from Lilongwe Medical Relief Trust (UNC Project-Malawi) and Cincinnati Children's Hospital Medical Center we plan to address this critical evidence gap by conducting a randomized controlled study determining bCPAP outcomes, compared to the currently recommended standard of care endorsed by the WHO and Malawi national pneumonia guidelines, in hospitalized high risk Malawian children with WHO-defined severe pneumonia complicated by a co-morbidity (HIV-infection, HIV-exposure without infection, severely malnourished) or WHO pneumonia with severe hypoxemia and without a co-morbidity. **We hypothesize that bCPAP will reduce the mortality of high risk Malawian children with WHO-defined severe pneumonia.**

Summary

Title: **Effectiveness of bubble CPAP in reducing childhood pneumonia mortality in Malawi**

Rationale: **Primary study (randomized controlled trial)**: Build evidence regarding whether treatment with bubble CPAP improves outcomes of African children with high risk WHO severe pneumonia complicated by a severe co-morbidity (severe malnourishment, HIV-infection, HIV-exposure), or WHO pneumonia with severe hypoxemia and without a co-morbidity.
Sub-study (observational study): Determine precise hospital mortality rates post-13 valent pneumococcal conjugate vaccine (PCV13) introduction for children with WHO severe pneumonia stratified by HIV and malnutrition status.

Population: **Primary study (randomized controlled trial)**: A total of **9,000 children** will be screened. We will accrue a total of 900 **children** ages 1-59 months of age (non-neonates) with WHO severe pneumonia and a co-morbidity (HIV-infection, HIV-exposure, severe malnutrition), or severe hypoxemia without a co-morbidity:

(1) About 600 children with WHO severe pneumonia and a co-morbidity (HIV-infection, HIV-exposed uninfected, severely malnourished) (300 per arm)
(2) About 300 severely hypoxemic children (150 per arm)
900 total children (450 per arm)

Sub-study (observational study): This sub-study will accrue 3,500 additional children 1-59 months of age with WHO severe pneumonia, but without HIV-infection, HIV-exposure, or severe malnutrition status (**therefore includes those screened ineligible for the primary study**). These children will be prospectively followed from hospital day 1 through hospital outcome in order to determine precise post-PCV13 mortality rates.

Focus Group Discussions: We will aim to conduct 5 total focus group discussions. Three focus groups with caregivers, and two with healthcare providers. Each focus group will consist of 6-8 caregivers or healthcare providers and will be conducted at Salima District Hospital. Overall our sample size for the focus group discussions will be **40 participants**.

Plan: **Primary study (randomized controlled trial):** Eligible volunteers will be randomized in an unblinded manner in a 1:1 ratio as follows:

Study groups	N	Day 1 until Hospital Outcome	30 day post-discharge phone call follow-up for vital status (if applicable)
Bubble CPAP (experimental group)	450	X	X
Control group	450	X	X

Sub-study (observational study): 3,500 additional eligible volunteers will receive standard care only and will be followed prospectively from hospital day 1 through hospital outcome.

Aims:

Primary study (randomized controlled trial):

1. **Aim 1 (primary)**: hospital pneumonia mortality in all children with WHO Severe pneumonia
2. **Aim 2 (secondary)**: hospital mortality, Day 6 and Day 14 treatment failure in children with WHO Severe hypoxemic pneumonia without co-morbidity (i.e., no HIV-infection and no HIV-exposure, and/or no severe malnutrition)
3. **Aim 3 (secondary)**: hospital mortality, Day 6 and Day 14 treatment failure in children with WHO Severe pneumonia and co-morbidity (i.e., HIV-infection or HIV-exposure, and/or severe malnutrition)
4. **Aim 4 (secondary)**: hospital mortality, Day 6 and Day 14 treatment failure treatment failure in children with WHO pneumonia and HIV-infection
5. **Aim 5 (secondary)**: hospital mortality, Day 6 and Day 14 treatment failure in children with WHO pneumonia and HIV-exposure
6. **Aim 6 (secondary)**: hospital mortality, Day 6 and Day 14 treatment failure in children with WHO pneumonia and severe acute malnutrition
7. **Aim 7 (secondary)**: hospital mortality, Day 6 and Day 14 treatment failure in children with WHO pneumonia and severe hypoxemia without HIV-infection or exposure, and/or severe malnutrition
8. **Aim 8 (secondary)**: presence of hypercarbic respiratory failure versus circulatory failure at initiation
9. **Aim 9 (secondary):** Compare changes in hypercarbic respiratory and circulatory failure between bCPAP and low-flow oxygen.
10. **Aim 10 (secondary)**: utilize biomarker panel to predict mortality risk at the time of study enrollment
11. **Aim 11 (secondary)**: adverse events (AEs)
12. **Aim 12 (secondary)**: Determine the mean time spent by health care workers administering care to patients on bCPAP and low-flow oxygen.
13. **Aim 13 (secondary)**: Determine the acceptability and feasibly of bCPAP among healthcare providers and caregivers of pediatric patients receiving bCPAP through focus group discussions.
14. **Aim 14 (secondary)**: 30 day outcome post-hospital discharge

Sub-study (observational study):

1. Determine precise post-PCV13 mortality rates in children with WHO severe pneumonia and receiving standard care, stratified by HIV and malnutrition status.

Outcomes: **Primary study (randomized controlled trial)**:

1. Primary outcome:
   - Proportion of high risk children surviving to hospital discharge among those with WHO severe pneumonia
2. Secondary outcomes:
   - Hospital mortality, and Day 6 and 14 treatment failure in children with WHO pneumonia and co-morbidity of HIV-infection
   - Hospital mortality, and Day 6 and 14 treatment failure in children with WHO pneumonia and co-morbidity of HIV-exposure
   - Hospital mortality, and Day 6 and 14 treatment failure in children with WHO pneumonia and co-morbidity of severe acute malnutrition
   - Hospital mortality, and Day 6 and 14 treatment failure in children with WHO pneumonia and severe hypoxemia without a comorbidity
   - Proportion of children with SAEs
   - 30 day outcome post-hospital discharge
   - Proportion of children with hypercarbic respiratory or circulatory failure at enrollment and respective changes in each after intervention (bCPAP vs standard care)
   - Measurement of the pediatric sepsis biomarker risk model (PERSEVERE) at enrollment (consisting of biomarkers CCL3, HSPA1B, IL8, GZMB and MMP8)
   - Proportion of children with SAEs
   - Proportion of children failing treatment by age, gender, hemoglobin level, oxygen saturation, malaria co-infection
   - Mean time spent by health care workers administering care to patients on bCPAP and low-flow oxygen.
   - Determine the acceptability and feasibly of bCPAP among healthcare providers and caregivers of pediatric patients receiving bCPAP through focus group discussions.

Sub-study (observational study):

2. Hospital outcomes of children with WHO severe pneumonia, stratified by HIV and malnutrition status.

Timeline: Projected duration of accrual is about 30 months.

All children in the randomized controlled trial and observational sub-study will be followed until hospital outcome. Those children in the randomized controlled trial that survive until hospital discharge will be followed for an additional 30 days after hospital outcome. (phone follow-up at 30 days post-discharge for vital status)

I. BACKGROUND

Despite laudable reductions in global childhood mortality rates,[1] pneumonia remains the second most frequent killer of children less than five years old worldwide.[2] Nearly one million children succumbed to pneumonia in 2013, with greater than half of these deaths in Africa.[2] In Malawi, which has a high prevalence of malnutrition and HIV infection, pneumonia is a major cause of pediatric mortality.[3] In patients with World Health Organization (WHO)-defined severe pneumonia, malnutrition, HIV-infection, and hypoxemia are the primary drivers of poor outcomes.[4] In a recent analysis of 2001-2012 child pneumonia outcomes in Malawi the overall case fatality rate decreased from 15% to 4% except in children with severe malnutrition (Colbourn T, et al. personal communication). The pneumonia mortality rate in malnourished children and those with severe hypoxemia remained elevated at 15% and 14% despite antibiotics and increased access to supportive interventions like low-flow supplemental oxygen (Colbourn T, et al. personal communication). Our observational data through January 2016 suggests that severely hypoxemic children may be as common as HIV-affected or severely malnourished cases, and may also have higher mortality (14.0%) than *non* HIV-affected, *non*-severely malnourished cases without severe hypoxemia (5.7%). In Malawian children with HIV-infection, WHO very severe pneumonia and severe malnutrition were the strongest predictors of death.[5] Non-invasive ventilation, already routinely used in industrialised countries, may provide an advanced treatment solution for certain patient populations such as children with WHO severe pneumonia complicated by severe malnutrition and/or HIV-infection or –exposure, or severe hypoxemia.[6-8]

Bubble continuous positive airway pressure (bCPAP) is non-invasive and is widely used for preterm neonatal respiratory failure in industrialised countries.[9,10] Along with a flow generator, bCPAP uses a water column to deliver continuous positive pressure to a spontaneously breathing child.[10] bCPAP is relatively inexpensive and requires little technical expertise compared with mechanical ventilation,[10] but there is limited experience of bCPAP in resource-poor settings. Recently, small studies have explored its use in preterm neonates in Malawi.[11,12] However, few studies have described its use in older infants and children,[13,14] none of which included mortality as a primary endpoint and specially focused on the main drivers of poor pediatric pneumonia outcomes in southern Africa, HIV, malnutrition, and hypoxemia.

Our data using bCPAP in Malawian children with severe pneumonia suggest feasibility for implementation.[13,14] We have previously reported that using a bCPAP system derived from locally available, relatively inexpensive supplies has shown promise in the management of hospitalized HIV-infected children with pneumonia in Malawi.[13] Our observational case series further delineates the outcomes of 77 Malawian children hospitalized at a tertiary referral facility with severe pneumonia who were treated with bCPAP.[14] Nearly half were infants either infected or exposed to HIV or were severely malnourished.[14] Although the mortality of this series of patients was 50.0%, bCPAP was initiated in this cohort only when patients were found to be failing standard treatment.[14] We estimated that more than 75% of these children would have been eligible for mechanical ventilation.[14] In this proposed study we will be initiating bCPAP earlier in the hospitalization prior to treatment failure. Unlike previous studies conducted at referral hospitals, we will perform this study at the district hospital level where 80% of hospitalized child pneumonia cases are cared for in Malawi (Colbourn T, et al. personal communication). In addition, the 13-valent pneumococcal conjugate vaccine (PCV13) was recently introduced throughout Malawi in late 2011 and there is limited WHO severe pneumonia mortality data at the district hospital level that includes rigorous evaluation of HIV and malnutrition status (Colbourn T, et al. personal communication).

Although bCPAP is relatively inexpensive, scale-up in countries like Malawi with significant pneumonia burden and high HIV prevalence will require substantial resources to meet expected

needs. In order to appropriately allocate precious resources and provide practical clinical guidance for healthcare providers who may use bCPAP, it is paramount to fully understand the utility of bCPAP treatment in this setting. To our knowledge no bCPAP data using a control group with mortality as the primary outcome has been reporting in a similar generalized HIV epidemic African patient population 1-59 months of age. Data generated from this research will be additionally critical for formulating future studies that may include bCPAP refinements or exploration of other feasible modalities like high-flow nasal cannula or bi-level positive airway pressure (e.g., BiPAP). Therefore, the more rigorous methodology proposed here is warranted and supported by the Malawi Ministry of Health. If bCPAP proves an effective treatment modality for children hospitalized with WHO severe pneumonia, it is a simple technology that could be operationalized to help thousands of children with life-threatening pneumonia.

We propose to address this critical evidence gap by conducting a randomized controlled study determining bCPAP outcomes, compared to the currently recommended standard of care endorsed by the WHO and Malawi Ministry of Health,[15] in hospitalized high risk Malawian children with WHO-defined severe pneumonia complicated by malnutrition, HIV-infection or –exposure, or severe hypoxemia. We also plan to leverage this controlled study to collect high quality observational hospital data on children 1-59 months with WHO severe pneumonia and receiving standard care, including those ***without*** malnutrition, HIV-infection, HIV-exposure, or severe hypoxemia, in order to determine precise mortality rates, stratified by HIV and malnutrition status, after full introduction of the pneumococcal conjugate vaccine. In addition, we will better define the clinical presentation of “severe pneumonia,” by differentiating hypercarbic respiratory failure from circulatory failure, and examining the effect of bCPAP on each. Finally, we will generate pilot data exploring the utility of a specific biomarker panel, previously validated to accurately estimate mortality risk among children with severe infection in high-resource settings, in this study setting.

II. APPROACH

Quality randomized studies comparing bCPAP versus a standard-of-care control group that includes low-flow oxygen therapy and using a primary endpoint of mortality are not available in low-resource settings including high prevalence HIV countries like Malawi for children 1-59 months of age with severe pneumonia. Demonstrating a mortality benefit with bCPAP is needed to support further investment and scale up of bCPAP in the care of older Malawian children 1-59 months of age with World Health Organization (WHO) severe pneumonia complicated by HIV, malnutrition, or severe hypoxemia. In addition, although PCV13 was introduced throughout Malawi in late 2011 there remains limited pneumonia mortality data stratified by HIV and malnutrition status, and this study can be leveraged to collect this data observationally to resolve this evidence gap.

III. HYPOTHESIS, AIMS AND OUTCOMES

- **Hypotheses**

  We hypothesize that bCPAP, compared to standard care, will reduce the pneumonia mortality of Malawian children with high risk WHO-defined severe pneumonia complicated by a severe co-morbidity (HIV-infection, HIV-exposure, severe malnutrition), or severe hypoxemia without a severe co-morbidity. The observational sub-study is not hypothesis driven.

- **Aims**

Randomized Controlled Trial

The broad objective of this study is to provide scientific evidence assessing the effectiveness of treatment with bCPAP for WHO severe childhood pneumonia for high risk children 1-59 months of age in Malawi, Africa.

Primary Aim

- Determine the hospital mortality rate for bCPAP treatment, compared to standard of care, for children with high risk WHO severe pneumonia.

Secondary Aims

- Aim 2
  - Determine the hospital mortality rate, and day 6 and 14 treatment failure rate for bCPAP treatment, compared to standard of care, for **HIV-infected** children with WHO severe pneumonia.
- Aim 3
  - Determine the hospital mortality rate, and day 6 and 14 treatment failure rate for bCPAP treatment, compared to standard of care, for **HIV-exposed, uninfected** children with WHO severe pneumonia.
- Aim 4
  - Determine the hospital mortality rate, and day 6 and 14 treatment failure rate for bCPAP treatment, compared to standard of care, for **severely malnourished** children with WHO severe pneumonia.
- Aim 5
  - Determine the hospital mortality rate, and day 6 and 14 treatment failure rate for bCPAP treatment, compared to standard of care, for **severely hypoxemic** children with WHO severe pneumonia and without a co-morbidity.
- Aim 6
  - Proportion of children with SAEs

- Aim 7
  - 30 day outcome post-hospital discharge
- Aim 8
  - Determine the prevalence of hypercarbic respiratory failure compared with circulatory failure among patients with clinical severe pneumonia
- Aim 9
  - Compare changes in hypercarbic respiratory and circulatory failure between bCPAP and low-flow oxygen.
- Aim 10
  - Determine the accuracy with which the PERSEVERE biomarker panel estimates mortality risk among patients with clinical severe pneumonia

- Additional secondary aims
  - To investigate whether there may be differential treatment responses in children with severe anemia, in those who test positive for malaria, in those with wheeze at baseline, and whether is a differential treatment response by age.
  - Determine the mean time spent by health care workers administering care to patients on bCPAP and low-flow oxygen.
  - Determine the acceptability and feasibly of bCPAP among healthcare providers and caregivers of pediatric patients receiving bCPAP through focus group discussions.

Observational sub-study

- Primary Aim
  - Determine precise hospital mortality rates for children 1-59 months old with WHO severe pneumonia stratified by HIV and malnutrition status.

- **Study Outcomes**

  Randomized Controlled Trial
  - Primary Outcome
    Proportion of in-hospital death in high risk children with WHO severe pneumonia. Eligible children will be either:
    - HIV-infected (<12 months of age with a positive HIV DNA PCR or >12 months with a positive HIV antibody test)
    - HIV-exposed, uninfected (<24 months old and tested negative for HIV but mother is HIV-infected)
    - Severely malnourished (weight-for-height of <-3 SD from the median and/or a mid-upper-arm circumference <115mm and/or bilateral edema)
    - Severely hypoxemic (oxygen saturation <90% in room air) without HIV-infection, HIV-exposure, or severe malnutrition

  - Secondary Outcomes
    1. Determine the hospital mortality, and day 6 and 14 treatment failure rate for bCPAP treatment, compared to standard of care, for **HIV-infected** children with WHO severe pneumonia.
    2. Determine the hospital mortality, and day 6 and 14 treatment failure rate for bCPAP treatment, compared to standard of care, for **HIV-exposed** children with WHO severe pneumonia.
    3. Determine the hospital mortality, and day 6 and 14 treatment failure rate for bCPAP treatment, compared to standard of care, for **severe malnourished** children with WHO severe pneumonia.
    4. Proportion of children with SAE
    5. Proportion of children alive at day 30 follow-up.

    Day 6 treatment failure is defined as the following:

    For **control group patients** treatment failure will be defined as any of the following between days 3-5:
    a. Fever >38° Celcius and low flow oxygen supplementation (1-2 LPM for children 2-59 months of age or 0.5 LPM for children 1-2 months of age) *or*
    b. Fever >38° Celcius and persistent respiratory danger signs (oxygen saturation <90%, grunting, head nodding, severe chest indrawing, or very fast breathing (>70 breaths/minute if 1-11 months; >60 breaths/minute if 12-59 months of age) or stridor in a calm child or apnea)
    c. Fever >38° Celcius and persistent general danger signs (inability to drink, lethargy or unconscious, convulsions)
    d. New respiratory danger sign (new oxygen saturation <90%, new grunting, head nodding, severe chest indrawing, or very fast breathing (>70 breaths/minute if 1-11 months; >60 breaths/minute if 12-59 months of age), stridor in a calm child, nasal flaring, or apnea) *or*
    e. New general danger sign (inability to drink, lethargy or unconscious, convulsions) *or*
    f. death at any time in the hospital treatment course prior to pneumonia cure (defined as the resolution of respiratory danger signs and fever >38° celcius, or continued need for low flow oxygen or bCPAP)

- For **experimental group patients** treatment failure between days 3-5 will be defined as follows:
  a) Fever ≥38° Celcius and bCPAP treatment *or*
  b) Fever ≥38° Celcius and persistent respiratory danger signs (oxygen saturation <90%, grunting, head nodding, severe chest indrawing, or very fast breathing (≥70 breaths/minute if 1-11 months; ≥60 breaths/minute if 12-59 months of age), stridor in a calm child, nasal flaring, or apnea)
  b. Fever ≥38° Celcius and persistent general danger signs (inability to drink, lethargy or unconscious, convulsions)
  c. New respiratory danger sign (new oxygen saturation <90%, new grunting, head nodding, severe chest indrawing, or very fast breathing (≥70 breaths/minute if 1-11 months; ≥60 breaths/minute if 12-59 months of age), stridor in a calm child, nasal flaring, or apnea) *or*
  d. New general danger sign (inability to drink, lethargy or unconscious, convulsions) *or*
  e. death at any time in the hospital treatment course prior to pneumonia cure (defined as the resolution of respiratory danger signs and fever ≥38° celcius, or continued need for low flow oxygen or bCPAP)

  All patients will also be considered a treatment failure if on day 14 the patient is not cured, defined as:
    a) an axillary fever ≥38.0 Celsius or
    b) presence of any respiratory danger sign (oxygen saturation <90%, grunting, head nodding, severe chest indrawing, very fast breathing (≥70 breaths/minute if 1-11 months of age; ≥60 breaths/minute if 12-59 months of age) , stridor in a calm child, nasal flaring, apnea) or
    c) continued need for low flow oxygen or bCPAP treatment.
- Proportion of children with SAEs.
- Proportion of children with treatment failure among those with severe anemia, with a positive test for malaria, and with wheeze at baseline.
- Proportion of children failing treatment by age at baseline.
- Hospital outcome of children with WHO severe pneumonia, irrespective of HIV-infection, HIV-exposure, or severe malnutrition status
- The proportion of hypercarbic respiratory failure compared with circulatory failure among patients with clinical severe pneumonia
- Changes in hypercarbic respiratory and circulatory failure between bCPAP and low-flow oxygen.
- Accuracy with which the PERSEVERE biomarker panel estimates mortality risk among patients with clinical severe pneumonia

Observational sub-study
- Primary Outcome
  Proportion of in-hospital death in children 1-59 months old with WHO severe pneumonia, irrespective of HIV or severe malnutrition status.

IV. METHODOLOGY

IV.1 DESIGN

The **randomized controlled trial** involves an unblinded, randomized, superiority trial in high risk children 1-59 months of age (non-neonates) from Malawi, Africa comparing the effectiveness of bCPAP to standard care low-flow oxygen for WHO severe community-acquired pneumonia. We plan to evaluate bCPAP versus standard care with low-flow oxygen, the current WHO-recommended therapy, among 900 children 1-59 months of age presenting with WHO severe pneumonia to a district hospital in Salima, Malawi. We also plan to leverage this controlled study to conduct an observational sub-study of high quality hospital data on children 1-59 months with WHO severe pneumonia and receiving standard care, including those ***without*** malnutrition, HIV-infection, or HIV-exposure, in order to determine precise mortality rates, stratified by HIV and malnutrition status, after full introduction of the pneumococcal conjugate vaccine.

IV.2 FIELD SITE

Johns Hopkins School of Medicine, the Malawi Ministry of Health, UNC Project, Primary Children's Utah, and Cincinnati Children's Hospital will partner to conduct the research at Salima District Hospital (SDH) in Lilongwe. A 250-bed government facility, SDH is a district hospital for the Salima district of Malawi, serving a population of approximately 350,000 and admitting about 7,000 children annually. Approximately 3 to 4 children 5 years of age or younger with WHO severe pneumonia are hospitalized daily at SDH.

SDH has one physician, four non-physician clinicians called clinical officers, and ten nurses on staff. The hospital has an OPD, triage area, and pediatric ward. The hospital has a basic laboratory unit and a radiology unit that can conduct basic laboratory testing and analogue x-rays.

IV.3 STUDY POPULATION

- **Study Population Overview**

  We expect study participants to be representative of the ethnic demographics in the study area. We anticipate enrolling equal numbers of female and male children in the primary study (randomized controlled trial) for a total of 900 volunteers. An additional 3,790 children with WHO severe pneumonia but without HIV infection, HIV-exposure, and severe malnutrition will be enrolled into the observational sub-study. Thus, a total of 4,680 children including the primary randomized controlled trial and observational sub-study will be enrolled (taking into account study withdrawals).

- **Participant Eligibility**

  Primary study participants (randomized controlled trial) of children 1-59 months of age with WHO severe pneumonia and a severe co-morbidity (i.e., HIV-exposure or HIV-infection and/or severe malnutrition), or WHO severe pneumonia with severe hypoxemia but without a co-morbidity will be eligible to participate.

  Observational sub-study children will be 1-59 months of age with WHO severe pneumonia but ***without*** HIV-infection, HIV-exposure, or severe malnutrition. Children 1-59 months of age with WHO severe pneumonia who are randomized to the **control group** in the primary randomized controlled trial will also be included in the analysis for this sub-study.

  Volunteer families will be recruited and screened, those whose children are determined to be eligible, based on the inclusion / exclusion criteria, will be enrolled in the randomized controlled trial, randomized, and followed for 30 days after hospital discharge (approximately 35-40 total days depending upon the duration of the hospitalization). Observational sub-study patients 1-59

months of age with WHO severe pneumonia who are ***without*** HIV-exposure, HIV-infection, severe malnutrition, or severe hypoxemia and **will not be randomized** and instead will have **observational data collected only**. These observational patients **will not be eligible for the bCPAP intervention** but will be able to receive other standard care treatments such as low-flow oxygen and antibiotics. Final eligibility determination will depend on the results of the medical history, clinical examination, appropriate understanding of the study and completion of the consent process.

For the **Time and Motion sub-study** we aim to determine the mean time spent by health care workers administering care to patients on bCPAP and low-flow oxygen. We hypothesize that the mean time spent by health care workers administering care to patients on bCPAP will be greater than mean time spent delivering care to patients on low-flow oxygen. Patients enrolled into the randomized controlled trial will be eligible for this aim and enrolled as described above. We will enroll a maximum of **68 subjects** to achieve 34 “observation periods.” We will define one observation period as 8 total hours of observation that includes 4 hours of observation immediately after enrollment (day 1) and another 4 hours of observation the morning after enrollment (day 2). Two patients can contribute to one observation period. For example, one observation period can be achieved by observing one patient for 4 hours after enrollment and another patient for 4 hours during the morning of day 1, as long as both patients are in the same study arm. All clinical activities will be timed and recorded on a data collection form by a trained observer. Only time spent by clinical and support staff directly involved in patient care will be recorded.

For the **Focus Group Discussions**, we hypothesize that healthcare provides administering bCPAP and caregivers of pediatric patients receiving bCPAP will find bCPAP to be feasible and acceptable. Caregivers of patients enrolled either arm of the study will be asked if they would like to participate in a focus group discussion to share opinions about bCPAP. Caregivers will provide written informed consent separately from their consent for their child to enroll in the study. We aim to conduct 5 total focus group discussions. Three focus groups with caregivers, and two with healthcare providers. Each focus group will consist of 6-8 caregivers or healthcare providers and will be conducted at Salima District Hospital. **In total we will include up to 40 participants in the focus group discussions**. Caregivers and healthcare providers will be allocated into separate focus groups. We will likely also stratify focus groups among caregivers by gender, and focus groups among healthcare provider by training cadre, if feasible. A trained moderator will lead the discussion about perceptions of bCPAP and the acceptability of bCPAP as a treatment for pneumonia. Discussions with caregivers or healthcare providers will be conducted in Chichewa, audio recorded, and translated to English. Focus group discussions will be audio recorded, transcribed, and translated into English. Translations will be back-transcribed into Chichewa to ensure translation validity. Themes and sub-themes will be identified and analyzed.

Case definition of WHO severe pneumonia[15]:

- Cough or difficulty breathing plus at least one of the following:
    - Oxygen saturation <90% on pulse oximetry (in room air) and/or any respiratory danger sign (grunting, severe chest indrawing, head nodding, tracheal tugging, nasal flaring, stridor in a calm child, or apnea)
    - signs of pneumonia (respiratory rate children $\geq$50 breaths/minute (for children 2 to <12 months of age) or $\geq$40 breaths/minute (for children $\geq$12 months of age) or chest indrawing or crackles +/- wheeze on auscultation or oxygen saturation 90-95% in room air) **plus** a general danger sign (inability to drink, vomiting everything, lethargy or unconscious, convulsions)

Definition of HIV-infected[15]

- child <12 months of age with a positive HIV DNA PCR or
- child $\geq$12 months with a positive HIV antibody test.

Definition of HIV-exposed, uninfected[15]

- <24 month old child who tested negative for HIV by DNA PCR or serology (HIV antibody test) and has a HIV-infected mother.

Definition of severe malnutrition[15]

- weight-for-height of <-3 SD from the median and/or
- mid-upper-arm circumference <115mm and/or
- bilateral pedal edema

Definition of severe hypoxemia[15]

- non-invasive peripheral oxygen saturation measurement <90% in room air

- **Inclusion Criteria for primary study (randomized controlled trial)**
  - Male or female, 1 to 59 months of age.
  - Meets WHO severe pneumonia criteria and is either HIV-infected, HIV-exposed, severely malnourished, or severely hypoxemic
  - Ability and willingness of children's caregiver to provide informed consent.

- **Inclusion Criteria for observational sub-study**
  - Male or female, 1 to 59 months of age.
  - Meets WHO severe pneumonia criteria and is either HIV-infected, HIV-exposed, or severely malnourished and is randomized to the control arm of the primary study
  - Meets WHO severe pneumonia criteria but is **not** HIV-infected, HIV-exposed, severely malnourished, or severely hypoxemic (thus not eligible for the randomized controlled trial)
  - Ability and willingness of children's caregiver to provide informed consent.

- **Exclusion Criteria**
  - Any psychosocial condition or circumstance that hinders study participation
  - Prior participation in the bCPAP study during a previous pneumonia diagnosis.

IV.4 STUDY PERIOD

All children participating in the randomized controlled trial or observational sub-study will be followed until hospital discharge. In addition, if the child survives to hospital discharge **and** is participating in the randomized controlled trial then the child will be followed until 30 days after discharge. The projected duration of accrual is anticipated to be about 30 months. The funding for this study is for three years, through April 30, 2018. This period includes the time required to prepare the necessary documents for the study, train all study personnel, initiate the study site, conduct the study and all data collection procedures, clean and analyze the data, and prepare the results for publication and presentation.

IV.5 SAMPLE SIZE

We will **screen 9,000 children** for WHO severe pneumonia at hospital admission (about 3,000 clinical pneumonia patients annually, of which approximately 20% will have severe pneumonia). We will **enroll** up to **4,680 children** with WHO severe pneumonia, irrespective of HIV, malnutrition, or severe hypoxemia status. Refer to Section 5, Statistical Design and Analysis, for more details.

The **primary study** (randomized controlled trial) will accrue an overall sample size of **900 children** (475 per treatment group, see Figure 1).

Figure 1. Study groups

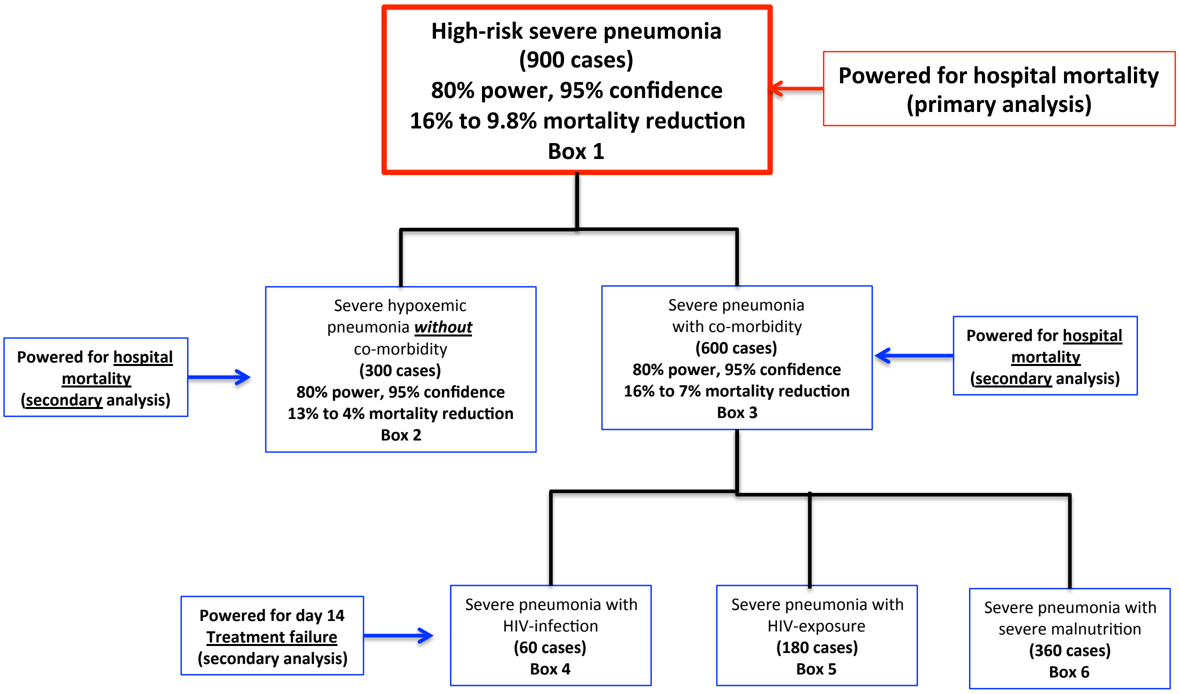

The "high-risk severe pneumonia" group (**Figure 1, Box 1**) is comprised of all groups and is powered for hospital pneumonia mortality. The "high-risk severe pneumonia" group will constitute our primary analysis group. The "severe pneumonia with co-morbidity" group (**see Figure 1, Box 3**) is comprised of three sub-groups, HIV-infection (**Box 4**), HIV-exposure (**Box 5**), and severe malnutrition (**Box 6**).. Each primary sub-group (severe hypoxemic pneumonia without co-morbidity (**Box 2**) and severe pneumonia with co-morbidity (**Box 3**)) is also powered for hospital pneumonia mortality. The individual co-morbidity groups (severe pneumonia with HIV-infection (**Box 4**), HIV-exposure (**Box 5**), or severe malnutrition (**Box 6**)) will be powered for day 14 treatment failure and will not be powered for hospital mortality. We aim to demonstrate a mortality reduction with bCPAP of about 60%, 15% control to 6% bCPAP, in each arm of the high-risk severe pneumonia group (**Figure, Box 1,** 450 children per treatment group**).** The overall composite mortality estimate for the "high risk severe pneumonia" group is determined from the relative contributions of each individual group as follows. Our sample size estimate for the analysis of the "severe hypoxemic without co-morbidity" sub-group (**Figure 1, Box 2)** will require 300 total cases (150 bCPAP, 150 standard care), assuming 13% mortality for standard care and 4% for bCPAP cases based on current literature (Chisti et al). Our sample size estimate for the analysis of the "severe hypoxemic with co-morbidity" sub-group (**Figure 1, Box 3)** will require 600 total cases (300 bCPAP, 300 standard care), assuming 16% mortality for standard care and 7% for bCPAP cases based on relative contributions by each sub-group. Specifically, the HIV-infection sub-group is estimated to contribute 10% of the overall group sample (60 cases) and have a control mortality of 30% and bCPAP mortality of 14%. The

individual HIV-exposure sub-group is estimated to contribute 30% (180 cases) of the overall sample and have a control mortality of 12% and bCPAP mortality of 5%. Lastly, the individual severe malnutrition sub-group is estimated to contribute 60% of the overall group sample (360 cases) and have a control mortality of 15% and bCPAP mortality of 7%. Although the severe pneumonia with co-morbidity subgroup and the severe hypoxemic without co-morbidity subgroup both have individual sample size estimates, we will permit enrollment above these sample estimates as this study's primary outcome is the combined group analysis. The overall sample size of the trial will not exceed 900 subjects. This allows the trial to most closely represent the true distribution of severe pediatric pneumonia cases at a Malawian district hospital and optimizes the reprsentativeness of the trial. All analyses will have 95% confidence and 80% power. Further details are provided in the Statistical Analysis Plan (SAP).

The **observational sub-study** will accrue an additional **3,500 children** 1-59 months of age that are screened, provide consent and found to have WHO severe pneumonia, but without HIV or severe malnutrition. Eligible children are those not included in the randomized controlled trial who are ***without*** HIV-infection, HIV-exposure, severe malnutrition, or severe hypoxemia (**3,500 patients**). In addition, children in the randomized controlled trial with HIV-infection, HIV-exposure, severe malnutrition, or severe hypoxemia who are randomized to the control arm (**750 patients**) will also be included in the analysis. Patients not enrolled in the primary study but enrolled in the observational substudy **only** will be followed until hospital outcome. The sample size for observational data collection is based upon an estimated overall severe pneumonia mortality rate of 4% with a precision of 0.65% and 95% confidence level. The aims related to venous blood gases will enroll 282 cases as detailed in the statistical analysis section. For the aim related to biomarker measurement, estimating a 17% mortality rate results in a sample size of 243 with 95% confidence and 0.05 margin of error. Therefore the sample size for the aim related to venous blood gases will be adequate for biomarker analysis.

IV.6 STUDY PROCEDURES

Figure 2. Study Flow Diagram

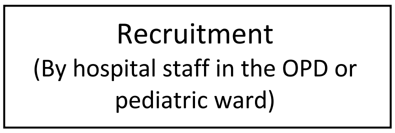

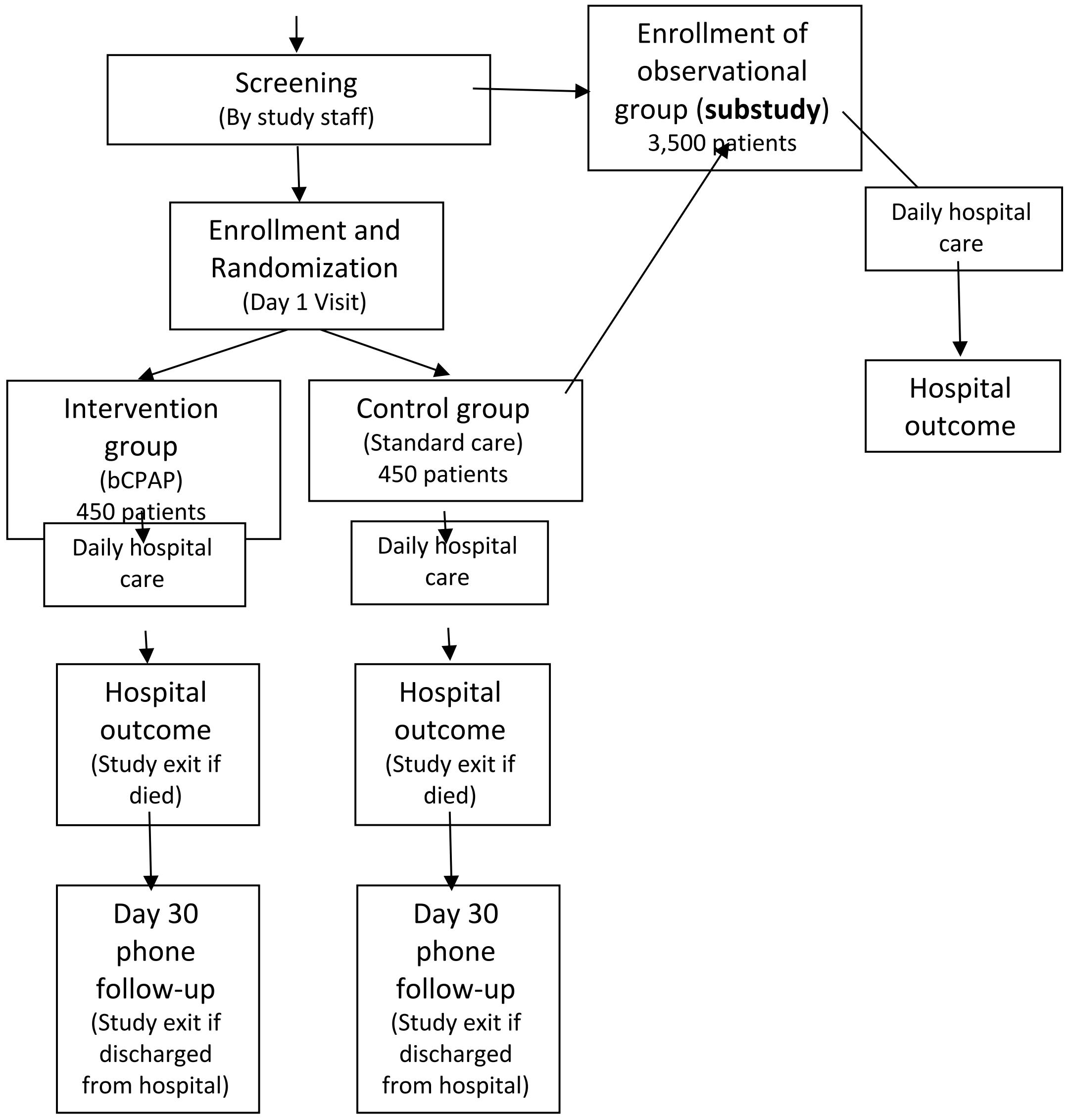

Refer to Appendix I for Study Procedures and Visits Table. Refer to Appendix II for Laboratory Specimens Collection, Timing and Distribution Table.

- **Recruitment**

  Recruitment for this study will be performed by SDH staff during admission to the pediatric ward. Children between 1 to 59 months of age presenting to the pediatric ward with cough or difficult breathing will be assessed by SDH staff. For any child with a cough and/or difficulty breathing, the clinician will inform the caregiver of the study. If the caregiver is interested in their child participating, he/she will be referred to the study. Routine care will not be delayed**.** Children 1-59 months of age that are screened, provide consent and have WHO severe pneumonia but are found to **not** have HIV-infection, HIV-exposure, or severe malnutrition will be provided the option of participating in the observational sub-study and being followed through hospital outcome.

  For the **Focus Group Discussions**, we will separately approach caregivers of patients enrolled either arm of the study and healthcare providers who administer CPAP to ask if they would like to participate in a focus group discussion to share opinions about bCPAP. Caregivers will provide written informed consent separately from their consent for their child to enroll in the study.

Healthcare providers will also provide separate written informed consent. Each focus group will consist of 6-8 caregivers or healthcare providers and will be conducted at Salima District Hospital.

- **Screening**

  Screening procedures are conducted by study staff to determine enrollment eligibility. All inclusion/exclusion criteria will be assessed and the following procedures will be performed:
    - Provide study information
    - Obtain written informed consent for screening **and** enrollment
    - Assign screening identification (ID) number
    - Assess eligibility criteria for the WHO severe pneumonia case definition, including respiratory rate, chest indrawing, pulse oximetry (while the patient is breathing in room air), presence of any danger signs (central cyanosis, grunting, head nodding, inability to drink, vomiting everything, lethargy or unconscious, convulsions)
    - If the child meets the WHO severe pneumonia case definition then additional screening will take place including:
        - a targeted physical examination including auscultation and an assessment for malnutrition (weight, MUAC, and examination for bipedal edema) will be done.
        - After obtaining consent in accordance with Malawi National HIV guidelines, a nurse or HIV counselor will collect blood for HIV antibody testing on all consenting participants if HIV status unknown *and* the child has met the WHO severe pneumonia case definition. Post-test counseling will be conducted in private by a nurse and/or HIV counselor.

  All screening procedures will be conducted by study staff. HIV testing will be performed by a study nurse or HIV counselor experienced in pediatric HIV counseling and testing in a private area. Caregivers will be informed of all screening results during the screening visit, regardless of the eligibility status of their child. Standard antibiotics and oxygen treatment (if respiratory danger signs) will not be delayed while this screening process is conducted.

  All screening procedures will be documented in the appropriate study forms, including logs and case report forms. Clinical assessments and findings will also be documented in the child's medical record.

- **Informed Consent**

  "Caregiver" refers to the child's legal acceptable representative (LAR) and informed consent must be obtained from the LAR. This study will have one informed consent form that includes **both screening and enrollment procedures**. Informed consent is the process of ensuring that caregivers of children fully understand what will and may happen to their children while participating in a research study. Study staff will ensure that participants fully comprehend the nature of the study through verbal confirmation prior to signing for informed consent. The informed consent process continues throughout the study. All consent materials will be approved by the Institutional Review Board (IRB). Refer to Section 6 for more details (Ethical Considerations and Consent).

- **Enrollment**

  Study staff will perform the enrollment procedures for eligible children after screening. For ineligible children, study staff will inform the caregiver(s) that child will receive standard care at SDH rather than study enrollment. The following enrollment procedures are performed:
    - Administer recommended antibiotics if not already administered by SDH staff

- Perform a physical exam to assess any baseline characteristics not already recorded in the medical record or assessed during screening, including digital auscultation and a 60 second video of the child's chest to assess breathing patterns and respiratory rates (the face will not be recorded)
- Patients eligible for the primary study (randomized controlled trial) will be **randomized**
  - **Intervention group (bCPAP)**
    - bCPAP will be administered by an oxygen concentrator and bCPAP machine with a nasal mask or prongs. A nasogastric tube will be placed for feeds on all patients at-risk for aspiration. A nasogastric tube will be placed for feeds on all patients at-risk for aspiration. Children with any general danger sign, 3 or more respiratory danger signs, or apnoea or grunting in isolation are not allowed to eat by mouth. Instead, these children will have all feeds held initially and once stable will receive a nasal gastric tube for feeding of expressed breast milk or formula every 2 hours at a standardized amount calculated to provide appropriate calories and fluid. Children with severe malnutrition receive nasal gastric feeds following the WHO protocol for severe malnutrition.
    - Patients randomized to bCPAP will be eligible for bCPAP treatment with the presence of any respiratory danger sign (oxygen saturation <90%, grunting, severe chest indrawing, very fast breathing ($\geq$70 breaths/minute if 1-11 months; $\geq$60 breaths/minute if 12-59 months, stridor in a calm child, nasal flaring, or apnea).
      - bCPAP will be initiated in children 2-59 months of age at 8cm H_20 or in children 1-2 months old at 7cm H_20 using the minimum oxygen flow necessary to achieve the pressure (estimated to be between 6-8 LPM from the oxygen concentrator per Fisher and Paykel bubble CPAP specifications). All bCPAP changes, including initiation, will be followed by 60 minutes of monitoring both the patient's oxygen saturation and breathing effort before additional changes are made (either increases or decreases).

  - **Control group (standard care)**
    - Patients randomized to the control group will receive low-flow oxygen supplementation with any respiratory danger sign. Low-flow oxygen will be administered by an oxygen concentrator with a nasal cannula per standard guidelines. Low-flow is 0.5 LPM for patients 1-2 months, and 1-2 LPM for patients 2-59 months. For 2-59 month olds oxygen will be initiated at 2 LPM. A nasogastric tube will be placed for feeds on all patients at-risk for aspiration. A nasogastric tube will be placed for feeds on all patients at-risk for aspiration. Children with any general danger sign, 3 or more respiratory danger signs, or apnoea or grunting in isolation are not allowed to eat by mouth. Instead, these children will have all feeds held initially and once stable will receive a nasal gastric tube for feeding of expressed breast milk or formula every 2 hours at a standardized amount calculated to provide appropriate calories and fluid. Children with severe malnutrition receive nasal gastric feeds following the WHO protocol for severe malnutrition.
  - If not already completed by SDH staff, perform full blood count (FBC) and malaria rapid diagnostic testing (mRDT). Those who are found to have malaria will receive appropriate antimalarial treatment using artemisinin-based combination therapy in addition to the randomly assigned treatment for pneumonia
  - A subset of randomized patients will have 1.5 mL of blood taken at enrollment for measurement of the pediatric sepsis risk biomarker panel, venous blood gas (VBG)

and lactate level. VBG and lactate will be measured using the iStat® handheld blood analyzer. The remaining blood will be refrigerated and transported to the research laboratory of UNC Project, Lilongwe, where it will be frozen at -80° C. When sample collection is complete, the frozen samples will be shipped on dry ice via WorldCourier® to Cincinnati Children's Hospital Medical Center for processing. Subsequent VBG/lactate levels will be measured at 1 hour and 6 hours after enrollment in a subset of patients. VBG and lactate levels will be considered research procedures only and will not be used during clinical care since these are not standard care laboratories in Malawi.

    - A subset of patients enrolled into the randomized controlled trial be observed by an independent observer to collect time and motion data related to their care. We will enroll a maximum of 68 subjects to achieve 34 "observation periods." We will define one observation period as 8 total hours of observation that includes 4 hours of observation immediately after enrollment (day 1) and another 4 hours of observation the morning after enrollment (day 2). Two patients can contribute to one observation period. For example, one observation period can be achieved by observing patient X for 4 hours after enrollment and patient Y for 4 hours during the morning of day 1, as long as both patients are in the same study arm. All clinical activities will be timed and recorded on a data collection form by a trained observer. Only time spent by clinical and support staff directly involved in patient care will be recorded.
    - For the **Focus Group Discussions**, caregivers of patients enrolled either arm of the study and healthcare providers who administer CPAP will be asked if they would like to participate in a focus group discussion to share opinions about bCPAP. Caregivers will provide written informed consent separately from their consent for their child to enroll in the study, as will healthcare providers. We aim to conduct 5 total focus group discussions. Three focus groups with caregivers, and two with healthcare providers. Each focus group will consist of 6-8 caregivers or healthcare providers and will be conducted at Salima District Hospital. Overall our sample size for the focus group discussions will be **40 participants**. Caregivers and healthcare providers will be allocated into separate focus groups. We will likely also stratify focus groups among caregivers by gender, and focus groups among healthcare provider by training cadre, if feasible.

  - Collect demographic and address information
  - Collect medical and vaccination history

  - Observational group

    - Patients eligible for the primary study and randomized to the control group will be enrolled as described above.
    - Patients eligible for the observational sub-study only (WHO severe pneumonia but without HIV-infection, HIV-exposure, severe malnutrition, or severe hypoxemia) will have the same enrollment procedures as the control arm in the randomized study, and not other respiratory danger signs. This distinction is made because these patients are relatively lower-risk than those with HIV-infection, HIV-exposure, severe malnutrition, or severe hypoxemia.

All enrollment procedures will be documented in the appropriate study forms. Clinical assessments and findings will also be documented in the child's medical record, as appropriate.

- **Randomization**

Randomization and enrollment occur together for eligible patients in the randomized controlled trial (Day 1). Randomization is defined as the process of assigning a child to a study arm; assignments will be computer-generated by individual patients and kept in unique sealed envelopes. The envelope seal will be broken at the time of randomization. Treatments will be allocated in a 1:1 ratio. Study investigators and staff will not be blinded to randomization allocation after randomization is completed.

- **Hospital Management**

Randomized controlled trial

<u>All randomized controlled trial participants</u>:

All patients will have their vital signs (temperature, heart rate, respiratory rate, oxygen saturation, and mental status) and respiratory effort assessed every 4-6 hours by study staff. A Rad5 Masimo pulse oximeter with an appropriately sized probe will be used to measure oxygen saturation and heart rate. All standard national Malawi guidelines will be followed for all enrolled patients including blood testing for malaria with rapid diagnostic testing (mRDT), full blood count (FBC), and routine opt-out provider initiated HIV testing and counseling as described in the screening and enrollment sections. All study participants, per Malawi national guidelines, will receive standard antibiotic treatment for at least five days.

Study children will be managed by study staff during the hospitalization. Diagnostic tests and medication for intercurrent illnesses will be ordered per ward protocols with results documented in study files. This includes antibiotic treatment regimen changes. Hospital clinicians will be available to consult with study staff about the clinical care of study participants as deemed appropriate by study staff.

<u>Treatment failure:</u>

All study participants, per Malawi national guidelines, will receive standard antibiotic treatment for at least five days.

For **control group patients** day 6 treatment failure will be defined as any of the following between days 3-5:

a. Fever ≥38° Celcius and low flow oxygen supplementation (1-2 LPM for children 2-59 months of age or 0.5 LPM for children 1-2 months of age) *or*
b. Fever ≥38° Celcius and persistent respiratory danger signs (oxygen saturation <90%, grunting, head nodding, severe chest indrawing, or very fast breathing (≥70 breaths/minute if 1-11 months; ≥60 breaths/minute if 12-59 months of age) or stridor in a calm child or apnea)
c. Fever ≥38° Celcius and persistent general danger signs (inability to drink, lethargy or unconscious, convulsions)
d. New respiratory danger sign (new oxygen saturation <90%, new grunting, head nodding, severe chest indrawing, or very fast breathing (≥70 breaths/minute if 1-11 months; ≥60 breaths/minute if 12-59 months of age), stridor in a calm child, nasal flaring, or apnea) *or*
e. New general danger sign (inability to drink, lethargy or unconscious, convulsions) *or*
f. death at any time in the hospital treatment course prior to pneumonia cure (defined as the resolution of respiratory danger signs and fever ≥38° celcius, or continued need for low flow oxygen or bCPAP)

- For **experimental group patients** day 6 treatment failure between days 3-5 will be defined as follows:

a) Fever >38° Celcius and bCPAP treatment *or*
b) Fever >38° Celcius and persistent respiratory danger signs (oxygen saturation <90%, grunting, head nodding, severe chest indrawing, or very fast breathing (>70 breaths/minute if 1-11 months; >60 breaths/minute if 12-59 months of age), stridor in a calm child, nasal flaring, or apnea)
c) Fever >38° Celcius and persistent general danger signs (inability to drink, lethargy or unconscious, convulsions)
d) New respiratory danger sign (new oxygen saturation <90%, new grunting, head nodding, severe chest indrawing, or very fast breathing (>70 breaths/minute if 1-11 months; >60 breaths/minute if 12-59 months of age), stridor in a calm child, nasal flaring, or apnea) *or*
e) New general danger sign (inability to drink, lethargy or unconscious, convulsions) *or*
f) death at any time in the hospital treatment course prior to pneumonia cure (defined as the resolution of respiratory danger signs and fever >38° celcius, or continued need for low flow oxygen or bCPAP)

All patients will also be considered a treatment failure if on day 14 the patient is not cured, defined as:

a) an axillary fever >38.0 Celsius or
b) presence of any respiratory danger sign (oxygen saturation <90%, grunting, head nodding, severe chest indrawing, very fast breathing (>70 breaths/minute if 1-11 months of age; >60 breaths/minute if 12-59 months of age) , stridor in a calm child, nasal flaring, apnea) or
c) continued need for low flow oxygen or bCPAP treatment.

In accordance with WHO and Malawi national guidelines, patients 1-11 months of age who are suspected to have HIV-infection (HIV antibody positive) or with documented HIV-infection will be treated for *Pneumocystis jirovecci* pneumonia with trimethoprim-sulfamethoxazole for 21 days and prednisolone for 5 days. Children >12 months of age with HIV-infection will receive treatment for *P.jirovecci* pneumonia only if clinically suspected at presentation (chest radiograph with interstitial pneumonia) *or* if the patient meets treatment failure criteria.

If the HIV antibody test is positive and the child is >12 months of age then the patient will be considered HIV-infected per Malawi guidelines. Children between 1-11 months of age will have a HIV DNA PCR dried blood spot (DBS) test. The DNA PCR test samples will be numbered and transported it to the Kamuzu Central Hospital laboratory in Lilongwe, Malawi for processing in accordance with standard procedures at SDH. All HIV counseling will be conducted in a private setting. Patients who are HIV-infected or HIV-exposed, uninfected will be referred to a HIV clinic in Salima at the time of hospital discharge.

A full blood count and mRDT (malaria rapid diagnostic test) will be collected on all participants at enrollment and malaria treatment will be conducted per standard guidelines. At enrollment (day 1), day 3, and day 6 (or day of hospital discharge) the patient will have their chest (with the exclusion of their face) video recorded with a hand-held device to determine respiratory rate over a one-minute period as well as respiratory effort. The video will be evaluated by a study physician as a quality check for the standardization of study staff. Lung auscultation with a digital stethoscope will also be performed at enrollment (day 1), day 3 and day 6 (or day of hospital discharge). The recordings will be evaluated by a study physician as a quality check of study staff. All randomized patients will have 1.5 mL of blood taken at enrollment for measurement of

the pediatric sepsis risk biomarker panel, venous blood gas (VBG) and lactate level. Subsequent VBG/lactate levels will be measured at 1 hour and 6 hours after enrollment. VBG and lactate levels will be considered research procedures only and will not be used during clinical care since these are not standard care laboratories in Malawi. Study staff will be masked to VBG and lactate results.

If the patient is discharged from the hospital and is a participant of the randomized control trial (primary study), then they will be contacted 30 days after hospital discharge by phone to determine their vital status (alive or dead).

<u>Low-flow oxygen supplementation (control group only of the randomized control trial)</u>: Patients randomized to the control group will receive low-flow oxygen supplementation if any respiratory danger sign is observed. Low-flow oxygen will be administered by an oxygen concentrator with a nasal cannula per standard guidelines. Low-flow is 0.5 LPM for patients 1-2 months, and 1-2 LPM for patients 2-59 months. For 2-59 month olds oxygen will be initiated at 2 LPM. A nasogastric tube will be placed for feeds on all patients at-risk for aspiration.

<u>Supplemental oxygen weaning:</u> Patients will be eligible for weaning if stable for at least 24 hours without any worsening disease severity (new respiratory danger signs). Every day, either in the morning or early afternoon when the hospital is at full staff, patients meeting weaning eligibility criteria will be weaned and rechecked after 60 minutes and if they remain stable in room air during the trial off period then the patient will be maintained off of oxygen. Patients will continue to have their respiratory danger signs monitored every 4-6 hours and oxygen re-started if respiratory danger signs recur during their hospitalization. For patients 2-59 months of age who are requiring more than 1 LPM oxygen, they will first be weaned down to 1 LPM and monitored for 60 minutes. If their respiratory danger signs remain stable then they will be trialed off of oxygen as described.

<u>bCPAP group (intervention group):</u> bCPAP will be administered by an oxygen concentrator and bCPAP machine with a nasal mask or prongs. A nasogastric tube will be placed for feeds on all patients at-risk for aspiration.

- Patients randomized to bCPAP will be eligible for bCPAP treatment with the presence of any respiratory danger sign (oxygen saturation <90%, grunting, severe chest indrawing, very fast breathing (≥70 breaths/minute if 1-11 months; ≥60 breaths/minute if 12-59 months, stridor in a calm child, nasal flaring, or apnea) or mild respiratory danger sign plus a general danger sign.
- bCPAP will be initiated at 7 or 8 cm H_20 (depending upon the child's age as described) using the minimum oxygen flow necessary to achieve that pressure (estimated to be between 6-8 LPM from the oxygen concentrator per Fisher and Paykel bubble CPAP specifications). All bCPAP changes, including initiation, will be followed by 60 minutes of monitoring both the patient's oxygen saturation and breathing effort before additional changes are made (either increases or decreases).

<u>bCPAP weaning:</u> Patients will be eligible for weaning if stable for at least 24 hours without any bCPAP escalation (increases in pressure or oxygen), respiratory danger signs (oxygen saturation <90%, head nodding, grunting, very severe chest indrawing, very fast breathing for age, nasal flaring, apnea, or stridor in a calm child). Weaning will be done in two steps. Step one is weaning from bCPAP to oxygen supplementation only. Step two is weaning off of supplemental oxygen to room air as described above.

Step one: Every day, either in the morning or early afternoon when at full staff, patients meeting weaning criteria will be weaned to 5cm H_20, followed by 60 minute observation periods or longer, using the minimum necessary flow to achieve these pressures (estimated to be between 6-8 LPM per Fisher and Paykel bubble CPAP specifications). If after 60 minutes, or longer, at 5cm H_20 the patient continues to have no respiratory danger signs then the patient will next be trialed off of bCPAP to supplemental oxygen only (1 LPM for patients 1-2 months of age and 1-2 LPM for patients 2-59 months of age). If for 60 minutes, or longer, there are no respiratory danger signs in room air during the bCPAP trial off period then the patient will be maintained off of bCPAP. Patients will continue to have their vital signs, respiratory effort, and oxygen saturation monitored every 4-6 hours with pulse oximetry and oxygen followed by bCPAP will be re-started if any respiratory danger sign recurs.

Step two: Patients are eligible for oxygen weaning if stable for another 24-48 hours after bCPAP without any worsening severity (new danger signs, worsening respiratory distress), restarting bCPAP, or a saturation <90%.

Observational sub-study patients

Observational sub-study patients that are not eligible for the randomized controlled trial will be enrolled according to the same procedures detailed above for the randomized controlled trial. After enrollment observational sub-study patients will receive their daily care primarily by government hospital staff, according to routine procedures and Malawi guidelines outlined previously. Study staff will prospectively track patient care on data collection forms. Patients will be followed until hospital outcome.

- **30 day phone follow-up**

  Target dates for phone follow-up are calculated from the date of hospital discharge for those patients cured and included in the primary study (randomized controlled trial). All 30 day phone follow-up calls must occur on the calendar day on which they are initially scheduled or within 48 hours afterwards. All phone calls will be placed to the LAR. If the LAR does not have a phone then no attempt will be made to contact the LAR in person. A study phone number will be provided to each LAR.

  The 30 day follow-up phone call procedure includes the following:
    - Update on patient's vital status (alive or dead)
    - Collect medical history since hospital discharge (additional illnesses that required medical care or hospitalizations)

  All follow-up phone calls will be documented.

- **Missed 30 day phone follow-up**

  Study staff will attempt to contact the patient's LAR by phone for an additional two consecutive days (day 31 and 32) if the LAR does not answer the phone on day 30 and then also on day 31. No further attempts will be made to contact the LAR beyond day 32 after hospital discharge, and the patient will exit the study. If the patient does not survive to hospital discharge then the LAR will not be contacted and the patient will exit the study.

- **Interim Contacts for randomized controlled trial participants**

Interim contacts (those between hospital discharge and regularly scheduled 30 day follow up phone call) may be performed at caregiver request or as deemed necessary by the site investigators. Interim visits will only occur at the hospital.

- **Withdrawal and Early Termination**

  Children and their caregivers may voluntarily withdraw from the study for any reason at any time. The site investigators may also withdraw children from the study for any reason at any time. Any participant withdrawal or early termination will be documented.

- **Study Termination Visit**

  The Day 30 follow-up phone call will serve as the study termination visit for the trial. Procedures for this visit, in addition to the standard 30 day follow-up phone call procedures described above, include the following:
  - Refer child to clinical care, as needed
  - Document contact in child's study records

- **Biohazards**

  All personnel will follow blood and secretion precautions recommended by the U.S. Centers for Disease Control.

IV.7 STUDY DEVICES

- **Nasal bCPAP (randomized controlled trial participants only)**

  This study will use an Airsep® oxygen concentrator and a Fisher & Paykel Bubble CPAP system (https://www.fphcare.com/products/bubble-cpap-system/) to deliver bCPAP. Therefore, when bCPAP is administered oxygen is also administered. The Airsep® machine is connected to the Fischer & Paykel Bubble CPAP system and the CPAP delivers pressure and oxygen to the patient with appropriately sized masks and tubing. The inspiratory limb of the ventilator tubing connects from the Fischer & Paykel Bubble CPAP system to the patient's nasal interface. The expiratory limb of the ventilator tubing connects from the patient's nasal interface to a reservoir of sterile saline embedded in the Fischer & Paykel Bubble CPAP system. The expiratory limb of the ventilator tubing is then inserted into a reservoir of sterile saline at a depth equivalent to the desired CPAP (e.g. 5 cm depth is equivalent to 5cm H_20 pressure). The Fischer & Paykel Bubble CPAP system can deliver up to 10cm H_20 pressure. CPAP nasal interface are used to interconnect the inspiratory and expiratory ventilator tubing limbs to the patient while creating a closed circuit capable of delivering pressure to the patient. Since an oxygen concentrator is being used as the flow driver of this bubble CPAP system patients receiving CPAP will therefore also be receiving 6-8 LPM of concentrated oxygen flow. Per manufacturer specifications the Airsep oxygen concentrator delivers 90-97% fractional inspired oxygen concentration at the 6-8 LPM flows required to generate 4-10 cm H_20 pressure. The Airsep® oxygen concentrator is approved

by the FDA and recommended by the WHO for use in low-resource settings. The Fischer & Paykel Bubble CPAP system is also approved by the FDA.

- **Supplemental oxygen**

  This study will use an Airsep® oxygen concentrator to deliver supplemental oxygen. The Airsep® oxygen concentrator is approved by the FDA and recommended by the WHO for use in low-resource settings.

- **Digital stethoscope**

  This study will use the ds32a Thinklabs digital stethoscope for the recording of lung sounds as a quality assurance and standardization check. This device is approved by the FDA.

- **Masimo Rad5 pulse oximeter**

  This study will use a Rad5 pulse oximeter for pulse rate and oxygen saturation measurements. This device is approved by the FDA.

- **Video recorder**

  A handheld video recorder will be used to video the child's breathing pattern and respiratory rate on selected hospitalization days as a quality assurance and standardization check. The video will not include the child's face.

- **iStat® handheld blood analyzer**

  This study will use the iStat® handheld blood analyzer to collect VBGs and lactate at enrollment, and 1 and 6 hours after enrollment.

Caregivers will be given the emergency contact number for the study personnel during the consenting process in order to report any adverse events. This same emergency contact number will also be available to SDH clinical staff.

- **Accountability**

  The study staff will regularly test the Airsep® oxygen concentrators to ensure that they are capable of delivering a minimum of 40% oxygen concentration. Study staff will also maintain complete records of which machines were utilized on patients and maintaining an accurate record of the randomization codes.

IV.8 DATA COLLECTION

Clinical research data will be maintained through secure electronic data management system and physical files with restricted access. Data related to study outcomes will be extracted from the electronic databases for statistical analysis. Two study databases will be created and maintained: the primary study database and a database with all participants' personally identifiable information. The first database containing study endpoint data will identify children by study identification numbers and will not contain identifying information. The study coordinator will also maintain a log that will contain the link between personal identifiers and the study participant IDs for the second database. The linklog and any other documentation (paper-based or electronic) that has both personal identifiers

and the participant ID will be stored in a secure manner (electronic data will be password protected and encrypted while physical source study documents will be maintained in a secure locked cabinet in the offices of UNC Project in Lilongwe, Malawi) separately from other study data and will be retained for at least five years after the last participant exits the study.

- **Case Report Forms**

  All study data will be collected by the clinical study staff using encrypted, password protected tablets for electronic data entry. Study data will be maintained on encrypted, password protected tablets during a hospitalization. Data will be transferred instantaneously from the encrypted, password protected tablet to the encrypted, password protected Johns Hopkins server. Electronic, password protected and encrypted data will be available for review by the senior study team on the Redcap server so that quality checks can be performed routinely. CRFs and laboratory reports will be reviewed by the site clinical team who are responsible for ensuring that they are accurate and complete. These data will be entered onto the encrypted, password protected tablets. The encrypted, password protected tablets will be kept in a secure location (study office in the hospital which is locked) to ensure confidentiality. Standard Good Clinical Practices (GCP) practices will be followed to ensure accurate, reliable and consistent data collection.

IV.9 DATA MANAGEMENT

Primary data management will be done by UNC Project. Data management includes data entry and validation, data coding and cleaning, database quality control, data recovery plans, and adverse event reporting.

- **Data Access**

  The participating site will maintain records in accordance with the International Conference on Harmonization Good Clinical Practice E6 (ICH-GCP) to protect participant confidentiality. Sponsor and regulatory agencies can examine clinical records for quality assurance reviews, audits and evaluation of the study safety and progress.

- **Data Storage**

  The site will maintain and store study records securely. All records will be maintained on site for at least five years after study completion.

- **External Study Monitoring**

  The Principal Investigator of the Study Sponsor is responsible for contacting and visiting the study site for the purpose of inspecting the facilities and record inspection. Monitoring visits will serve to verify compliance with human subjects and other research regulations and guidelines, assess adherence to the study protocol and study-specific procedures manual, confirm the quality and accuracy of information collected at the study site and entered into the study database, assess the resolution of any past or ongoing issues identified at previous monitoring visits.

IV.10 SAFETY

- **Safety Monitoring**

This protocol has extensive safety monitoring in place. The study site investigators will be responsible for close safety monitoring of all children participating in the study, and for alerting the protocol team if unexpected concerns arise. Each study subject participating in the randomized trial (primary study) will be evaluated by a study clinician or nurse at least twice daily while hospitalized and will be additionally monitored by study nursing staff or study vital sign assistants including every four hourly vital signs (normally vital signs are not done at all). Overnight care will be provided by study nurses, study vital sign assistants and SDH staff. An emergency number will be provided to all SDH staff and study participants so that an on-call study clinician or nurse can be reached at any time during study participation. As needed, participants may be evaluated at interim visits and/or referred for additional care. AEs will also be regularly reviewed by study personnel and compiled into reports. The study team may seek independent expert medical opinion as the need arises.

- **Data Safety and Monitoring Board (DSMB)**

An **independent DSMB** will be set up to regularly review cumulative safety and study conduct data after 30% and 60% of overall study enrollment which should occur after approximately 10 and 20 months of data collection. The DSMB will include at least two physicians and one biostatistician. The DSMB will consider early termination of the study if there is substantial evidence of a meaningful and significant mortality difference between the bCPAP and standard care groups in the randomized controlled trial (primary study). The DSMB will employ appropriate methods for group-sequential interim analyses.

Specifically, interim outcome analyses will be conducted for the two study groups using logistic regression with Hospital pneumonia outcome (pneumonia cure vs. died before pneumonia cure) as the outcome and group assignment as the treatment variable. An O'Brien-Fleming (1979) stopping rule will be used to reduce the problem of inflated type II error due to multiple comparisons. Based on Piantadosi (2005), p-values (and Z-score cutoffs) for two-sided tests used in the first interim, second interim, and final analyses will be .0006 (3.438), .0151 (2.431), and .0471 (1.985), respectively. If there is significant imbalance between groups on baseline covariates, results of logistic regression analyses controlling for these variables will also be reported. If missing data do not appear to be missing completely at random (MCAR), we will use multiple imputation methods to model the missinginess mechanism based on observed data and to evaluate whether the study findings vary when accounting for missingness.

The content, format and frequency of safety data reports will be agreed upon by the principal investigator and the DSMB. The DSMB reviews will be summarized with recommendations to the IRBs of record, as to whether or not there are safety concerns and that the study should continue without change, be modified, or terminated.

In the event that the protocol team has serious safety concerns that lead to a decision to stop accrual into the study, the principal investigator will request a review of the data by the DSMB before recommending that the study be stopped.

- **Adverse Events**

An adverse event (AE) is any unfavorable or unintended change in body structure, body function or laboratory result associated temporally with the use of a study device, whether or not considered to be related to the study device. LAR will contact the study site staff to report any AEs. All AEs will be managed by the study team per standard medical care at the hospital. All AE will be followed until resolution or stabilization.

- **Serious Adverse Event**

  Serious adverse events (SAEs) are defined as those that result in death, are life-threatening, merit hospitalization or prolongation of hospitalization, or persistent or significant disability/incapacity. Any readmission to the hospital will also be reported as an SAE.

- **Adverse Event Relationship to Study Device**

  The relationship of AEs to the study device will be assessed according to the below:
  - Definitely related: adverse event and administration of study device are related in time, and a direct association can be demonstrated with the study device.
  - Probably related: adverse event and administration of study device are reasonably related in time, and the adverse event is more likely explained by the study device than by other causes.
  - Possibly related: adverse event and administration of study device are reasonably related in time, and the adverse event can be explained equally well by causes other than the study device.
  - Probably not related: a potential relationship between administration of study device and adverse event could exist, but is unlikely, and the adverse event is most likely explained by causes other than the study device.
  - Not related: the adverse event is clearly explained by another cause unrelated to administration of the study device. Reportable events must have documentation to support the determination of "not related".

- **Grading Severity of Events**

  All adverse events will be graded by the widely used DAIDS AE Grading Table Version 1.0, December 2004; clarification August 2009. This grading table is now adopted by the U.S. Food and Drug Administration (U.S. FDA) for AE reporting. This table is available at: http://rcc.tech-res.com/Document/safetyandpharmacovigilance/Table_for_Grading_Severity_of_Adult_Pediatric_Adverse_Events.pdf

- **Safety Reporting**

  Attribution with regard to relationship to study device will only be reported for AE grades 3, 4, or 5 and for all SAEs. Any Grade 4 local or systemic reactogenicity symptom or AE, described by the site staff as possibly, probably, or definitely related to the study device, or any Grade 5 event requires immediate notification by the site to the PI. The PI and co-investigators will decide whether the event necessitates a pause in further enrollment. If the PI and co-investigators cannot convene to review the event within 72 hours, the medical officer will make the final decision.

  Reporting requirements for the IRB will be followed as appropriate.

- **Study Discontinuation**

  The trial may be discontinued at any time by the sponsor, funding agency, Malawi regulatory authorities, or institutional review board.

V.STATISTICAL DESIGN AND ANALYSIS

V.1 DATA ANALYSIS

- **Overview and General Design**

  In brief, we plan to conduct a facility-based, randomized, superiority trial of bCPAP (intervention) versus standard care (control) for WHO severe pneumonia as a **primary study**.

  We also plan to leverage this randomized controlled study to conduct an **observational sub-study** of high quality hospital data on children 1-59 months with WHO severe pneumonia and receiving standard care, including those ***without*** malnutrition, HIV-infection, HIV-exposure, or severe hypoxemia, in order to determine precise mortality rates, stratified by HIV and malnutrition status, after full introduction of the pneumococcal conjugate vaccine.

  The study will screen 9,000 children and accrue 4,680 total patients aged 1 to 59 months presenting with WHO severe pneumonia at Salima District Hospital (SDH) in Salima, Malawi. The primary randomized controlled study will accrue 900 children (450 children per study arm) and is powered for hospital pneumonia mortality (see Figure 1, Box 1). In addition, there are 2 sub-groups also powered for hospital pneumonia mortality; (i) 300 children with WHO severe hypoxemic pneumonia without co-morbidity (HIV-uninfected, HIV-unexposed, and not severely malnourished; 175 per group, Figure 1, Box 2); (ii) 600 children with WHO severe pneumonia with co-morbidity (HIV-infection, HIV-exposed, severe malnutrition) (Figure 1, Box 3). The WHO severe pneumonia with co-morbidity group will be comprised of 600 children in the following approximate distribution; 60 HIV-infected children (30 per group); (ii) 180 HIV-exposed, uninfected children (90 per group); (iii) 360 severely malnourished children (HIV-uninfected and HIV-unexposed; 180 per group). Treatment will be randomized individually in sealed envelopes to ensure a 1:1 ratio of intervention and control. Up to an additional 3,500 children aged 1 to 59 months presenting with WHO severe pneumonia but without HIV-infection, HIV-exposure, or severe malnutrition will be enrolled into the observational substudy (total substudy enrollment of 3,975 children (475 randomized controlled trial standard care arm + 3,500 observational standard care**)**. The aims related to venous blood gases and biomarker measurement will enroll 282 cases as detailed in the statistical analysis section.

- **Randomization Procedures**

  Randomization will be performed as 1:1 and will not be blinded.

- **Objectives, Endpoints, and Analytical Methodology for Final Analyses**

  Randomized Controlled Trial (primary study)

  For the randomized controlled trial (primary study) the primary null hypothesis will be that the primary outcome of survival to hospital discharge is no different in those who received bCPAP (superior in the alternative hypothesis) to those who received standard care for the treatment of WHO severe pneumonia in children aged 1–59 months complicated by HIV-infection, HIV-exposure, severe malnourishment (but not HIV-affected), or severe hypoxemia (but not HIV-infected, HIV-exposed, or severely malnourished). Eligible children will be randomly assigned to receive bCPAP in the intervention group and standard care with low-flow supplemental oxygen in the control group. The children will be evaluated every 4-6 hours after enrollment, or more frequently if needed, until hospital outcome to assess for response to treatment or development of treatment failure. If a child becomes ill again after hospital discharge after having returned to normal, that child will be encouraged to return between hospital discharge and day 14 for reassessment. Children who develop adverse reactions to the study device or withdraw from the study will be treated according to Malawian standard guidelines.

Observational sub-study

Patients eligible for the primary study and randomized to the standard care group will be enrolled as described above.

Patients eligible for the observational sub-study only (WHO severe pneumonia but **without** HIV-infection, HIV-exposure, severe malnutrition, or severe hypoxemia) will have the same enrollment procedures as the standard care group in the randomized study. This distinction is primarily made because these patients are assumed to be relatively lower-risk than those with HIV-infection, HIV-exposure, severe malnutrition, or severe hypoxemia.

Randomized Controlled Trial

- Primary Endpoints
  Proportion of in-hospital death in children with WHO severe pneumonia. Eligible children will be either:
    - HIV-infected (<12 months of age with a positive HIV DNA PCR or ≥12 months with a positive HIV antibody test)
    - HIV-exposed, uninfected (<24 months old and tested negative for HIV but mother is HIV-infected)
    - Severely malnourished (weight-for-height of <-3 SD from the median and/or a mid-upper-arm circumference <115mm and/or bilateral edema)
    - Severe hypoxemia (oxygen saturation <90% in room air, not HIV infected, HIV-exposed, or severely malnourished)

- Secondary Endpoints (randomized controlled trial)
    - Hospital mortality, and day 6 and 14 treatment failure rate for bCPAP treatment, compared to standard of care, for **HIV-infected** children with WHO severe pneumonia.
    - Hospital mortality, and day 6 and 14 treatment failure rate for bCPAP treatment, compared to standard of care, for **HIV-exposed** children with WHO severe pneumonia.
    - Hospital mortality, and day 6 and 14 treatment failure rate for bCPAP treatment, compared to standard of care, for **severe malnourished** children with WHO severe pneumonia.
    - Proportion of children with SAE
    - Proportion of children alive at day 30 follow-up.
    - Proportion of children with treatment failure among those with severe anemia, with a positive test for malaria, and with wheeze at baseline.
    - Proportion of children failing treatment by age at baseline.
    - Determine the prevalence of hypercarbic respiratory failure compared with circulatory failure among patients with clinical severe pneumonia
    - Compare changes in hypercarbic respiratory and circulatory failure between bCPAP and low-flow oxygen.
    - Determine the accuracy with which the PERSEVERE biomarker panel estimates mortality risk among patients with clinical severe pneumonia

- **Treatment failure**:

For **control group patients** day 6 treatment failure will be defined as any of the following between days 3-5:

a. Fever ≥38° Celcius and low flow oxygen supplementation (1-2 LPM for children 2-59 months of age or 0.5 LPM for children 1-2 months of age) *or*

b. Fever >38° Celcius and persistent respiratory danger signs (oxygen saturation <90%, grunting, head nodding, severe chest indrawing, or very fast breathing (>70 breaths/minute if 1-11 months; >60 breaths/minute if 12-59 months of age) or stridor in a calm child or apnea)
c. Fever >38° Celcius and persistent general danger signs (inability to drink, lethargy or unconscious, convulsions)
d. New respiratory danger sign (new oxygen saturation <90%, new grunting, head nodding, severe chest indrawing, or very fast breathing (>70 breaths/minute if 1-11 months; >60 breaths/minute if 12-59 months of age), stridor in a calm child, nasal flaring, or apnea) *or*
e. New general danger sign (inability to drink, lethargy or unconscious, convulsions) *or*
f. death at any time in the hospital treatment course prior to pneumonia cure (defined as the resolution of respiratory danger signs and fever >38° celcius, or continued need for low flow oxygen or bCPAP)

- For **experimental group patients** day 6 treatment failure between days 3-5 will be defined as follows:

a. Fever >38° Celcius and bCPAP treatment *or*
b. Fever >38° Celcius and persistent respiratory danger signs (oxygen saturation <90%, grunting, head nodding, severe chest indrawing, or very fast breathing (>70 breaths/minute if 1-11 months; >60 breaths/minute if 12-59 months of age), stridor in a calm child, nasal flaring, or apnea)
c. Fever >38° Celcius and persistent general danger signs (inability to drink, lethargy or unconscious, convulsions)
d. New respiratory danger sign (new oxygen saturation <90%, new grunting, head nodding, severe chest indrawing, or very fast breathing (>70 breaths/minute if 1-11 months; >60 breaths/minute if 12-59 months of age), stridor in a calm child, nasal flaring, or apnea) *or*
e. New general danger sign (inability to drink, lethargy or unconscious, convulsions) *or*
f. death at any time in the hospital treatment course prior to pneumonia cure (defined as the resolution of respiratory danger signs and fever >38° celcius, or continued need for low flow oxygen or bCPAP)

All patients will also be considered a treatment failure if on day 14 the patient is not cured, defined as:

a. an axillary fever >38.0 Celsius or
b. presence of any respiratory danger sign (oxygen saturation <90%, grunting, head nodding, severe chest indrawing, very fast breathing (>70 breaths/minute if 1-11 months of age; >60 breaths/minute if 12-59 months of age) , stridor in a calm child, nasal flaring, apnea) or
c. continued need for low flow oxygen or bCPAP treatment.

Observational sub-study

- Primary Endpoint
  Proportion of in-hospital death in children 1-59 months old with WHO severe pneumonia, irrespective of HIV or severe malnutrition status or hypoxemia status.

For the **primary study** (randomized controlled trial) recruitment and follow-up is expected to continue until the maximum sample size is achieved. We are assuming that a total of 900 children (450 per treatment group) will be enrolled, and given the prevalence of malnutrition and HIV and hypoxemia in children with severe pneumonia we expect this sample size can be met with

screening 9,000 children (about 3,000 children with clinical pneumonia annually). The primary randomized controlled study will accrue 900 children (450 children per study arm) and is powered for hospital pneumonia mortality (see Figure 1, Box 1). In addition, there are 2 sub-groups also powered for hospital pneumonia mortality in the approximate distributions; (i) 300 children with WHO severe hypoxemic pneumonia without co-morbidity (HIV-uninfected, HIV-unexposed, and not severely malnourished; 150 per group, Figure 1, Box 2); (ii) 600 children with WHO severe pneumonia with co-morbidity (HIV-infection, HIV-exposed, severe malnutrition) (Figure 1, Box 3). The WHO severe pneumonia with co-morbidity group will be comprised of 600 children in the following approximate distribution; 60 HIV-infected children (30 per group); (ii) 180 HIV-exposed, uninfected children (90 per group); (iii) 360 severely malnourished children (HIV-uninfected and HIV-unexposed; 180 per group).

Our sample size estimate for the analysis of the "severe hypoxemic without co-morbidity" sub-group (**Figure 1, Box 2)** will require 300 total cases (150 bCPAP, 150 standard care), assuming 13% mortality for standard care and 4% for bCPAP cases based on current literature (Chisti et al). Our sample size estimate for the analysis of the "severe hypoxemic with co-morbidity" sub-group (**Figure 1, Box 3)** will require 600 total cases (300 bCPAP, 300 standard care), assuming 16% mortality for standard care and 7% for bCPAP cases based on relative contributions by each sub-group. Specifically, the HIV-infection sub-group is estimated to contribute 10% of the overall group sample (60 cases) and have a control mortality of 30% and bCPAP mortality of 14%. The individual HIV-exposure sub-group is estimated to contribute 30% (180 cases) of the overall sample and have a control mortality of 12% and bCPAP mortality of 5%. Lastly, the individual severe malnutrition sub-group is estimated to contribute 60% of the overall group sample (360 cases) and have a control mortality of 15% and bCPAP mortality of 7%. All analyses will have 95% confidence and 80% power. These assumptions for all objectives take into consideration a 5% absconding rate, 80% power and a confidence level of $p < 0.05$.

Figure 1. Flow diagram of study groups and sample sizes

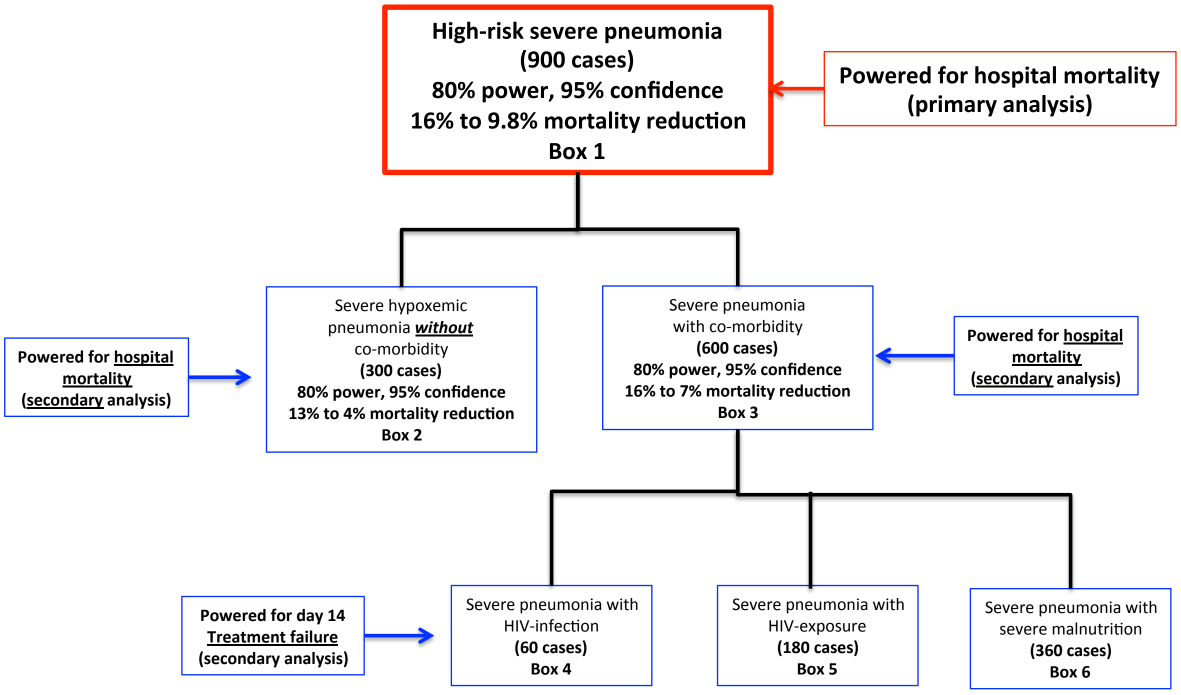

For the **observational sub-study** we aim to determine precise post-PCV13 mortality rates in children with WHO severe pneumonia and receiving standard care, stratified by HIV and malnutrition status. If we assume an overall mortality rate of 4% and aim to have a precision of 0.65% and 95% confidence level, we will need a sample size of 3,500 children.

Therefore, the overall enrollment taking into account the primary study (900) and substudy (3,500) is 4,680 patients. This includes patient defaulting and withdrawals from the study.

For the blood gas aims we will enroll a minimum of 284 subjects. Assuming a conservative pre/post correlation of r=0.3, to detect a 5 point difference under our ANCOVA models to achieve 80% power at a 0.05 significance level, we would need a total sample size of 142 (71/group). We estimate 50% of children will experience respiratory failure in our study. (Given the lack of published data on respiratory failure in severe pneumonia in developing countries, this estimate is based on clinical experience.) We therefore plan to enroll a minimum of 284 subjects.

We will determine the prevalence of hypercarbic respiratory failure, defined as a pre-therapy pCO_2 of 45 mmHg or greater, and circulatory failure, defined as a pre-therapy lactate of 2 mmol/L or greater, and the associated 95% confidence intervals (CIs). CIs will be based on the normal approximation to the binomial distribution. For Aim 9, we will use Analysis of Covariance (ANCOVA) models to compare the mean change in pCO_2 between the low-flow oxygen and bCPAP after controlling for pre-therapy pCO_2 levels. Adjustment for pre-therapy pCO_2 level accounts for variation due to random baseline imbalances and regression to the mean, thereby increasing statistical power. Our secondary outcome of circulatory failure will be similarly analyzed.

For the biomarker aim, we will measure the PERSEVERE biomarkers and compare the PERSEVERE score, as calculated by the originally derived decision tree, to determine whether PERSEVERE is prognostic for mortality. Correlation between the PERSEVERE score and mortality will be evaluated using simple logistic regression. We will then use classification and regression tree (CART) analysis to determine whether the addition of specific clinical variables (age, gender, comorbidity), in addition to biomarker measurements, improve the predictive ability of the model.

For the time motion analysis aim, we will enroll a maximum of 68 subjects participating in the randomized trial. If we assume a standard deviation of 10 minutes in either study arm, 34 observation periods (17 bCPAP, 17 low-flow oxygen) will enable the detection of a minimum difference of an average of 10 minutes in total care administered between the study arms at 80% power and at a 0.05 significance level. We will observe and time the interactions between health care workers and patients receiving both bCPAP and low-flow oxygen. We will use a time and motion analyses to determine the mean time spent on unique tasks. We will compare the time spent on care for bCPAP patients with low-flow oxygen patients. Monetary values will then be assigned to the time spent on each patient based on a corresponding percentage of the health worker’s monthly salary. Cost analysis from the sample population will then be extrapolated to predict the cost over one year, as this is the most common budgeting period for a health facility.

For the **Focus Group Discussions**, we will aim to conduct 5 total focus group discussions. Three focus groups with caregivers, and two with healthcare providers. Each focus group will consist of 6-8 caregivers or healthcare providers and will be conducted at Salima District Hospital. Overall our sample size for the focus group discussions will be **40 participants**. Caregivers and healthcare providers will be allocated into separate focus groups. We will likely also stratify focus groups among caregivers by gender, and focus groups among healthcare provider by training cadre, if feasible.

Tables. Required total sample sizes for mortality rates in the primary randomized controlled trial bCPAP and standard care groups and main sub-groups of children with WHO severe hypoxemic pneumonia without co-morbidity (Figure 1, Box 2) and WHO severe pneumonia without co-morbidity (Figure 1, Box 3; HIV-infected, HIV-exposed, and severely malnourished) to achieve 80% power with 95% confidence.

A. WHO severe pneumonia (all groups; Figure 1, Box 1).

B. WHO

		**Control group mortality rate**				
		**13%**	**14%**	**15%**	**16%**	**17%**
**bCPAP mortality rate**	**4%**	294	250	216	190	168
	**5%**	394	326	276	336	206
	**6%**	544	434	358	300	258
	**7%**	778	596	474	388	324
	**8%**	1174	846	646	512	418

severe hypoxemic pneumonia without co-morbidity (Figure 1, Box 2)

		**Control group mortality rate**			
		**10%**	**11%**	**12%**	**13%**
**bCPAP mortality rate**	**2%**	270	228	198	174
	**3%**	382	312	262	224
	**4%**	560	438	354	290
	**5%**	864	636	492	394

C. WHO severe pneumonia with co-morbidity (Figure 1, Box 3; HIV-infection, HIV-exposure, severe malnutrition)

E.

		**Control group mortality rate**				
		**14%**	**15%**	**16%**	**17%**	**18%**
**bCPAP mortality rate**	**5%**	326	276	336	206	182
	**6%**	434	358	300	258	224
	**7%**	596	474	388	324	276
	**8%**	846	646	512	418	348
	**9%**	1272	914	694	548	446

Observational sub-study

		**Observational group mortality rate**			
		**2%**	**3%**	**4%**	**5%**
**Precision**	**0.5%**	3,012	4,472	5,901	7,299
	**0.55%**	2,490	3,696	4,877	6,033

	**0.6%**	2,092	3,106	4,098	5,069
	**0.65%**	1,783	2,646	3,492	4,319
	**0.7%**	1,537	2,282	3,011	3,724

Sample size estimates were derived from a combination of sources including preliminary data from our study team collected in Malawi that reported a mortality of 50% in children meeting the WHO severe pneumonia case definition with bCPAP.[14] In this study bCPAP was initiated only when the child failed treatment. This study differs in that bCPAP may be started at enrollment as in this study for the intervention arm. Given the lack of data in this area of study we extrapolated our rough 50% mortality reduction estimates with bCPAP from this study. In addition, we utilized the first 10 months of study data to provide better estimates for patient accrual of the study. Using this preliminary data but maintaining our a priori treatment effect estimates to avoid any bias, we aim to detect a mortality reduction with bCPAP of about 60%, 15% control to 6% bCPAP, in each arm of the high-risk severe pneumonia group (**Figure, Box 1,** 450 children per treatment group**).** The overall composite mortality estimate for the "high risk severe pneumonia" group is determined from the relative contributions of each individual group from the year 1 data as described previously. Our sample size estimate for the analysis of the "severe hypoxemic without co-morbidity" sub-group (**Figure 1, Box 2)** will require approximately 300 total cases (150 bCPAP, 150 standard care), assuming 13% mortality for standard care and 4% for bCPAP cases based on current literature (Chisti et al). Our sample size estimate for the analysis of the "severe hypoxemic with co-morbidity" sub-group (**Figure 1, Box 3)** will require 600 total cases (300 bCPAP, 300 standard care), assuming 16% mortality for standard care and 7% for bCPAP cases based on relative contributions by each sub-group from year 1 data. Specifically, the HIV-infection sub-group is estimated to contribute 10% of the overall group sample (60 cases) and have a control mortality of 30% and bCPAP mortality of 14% based upon the literature. The individual HIV-exposure sub-group is estimated to contribute 30% (180 cases) of the overall sample and have a control mortality of 12% and bCPAP mortality of 5% based upon the literature. Lastly, the individual severe malnutrition sub-group is estimated to contribute 60% of the overall group sample (360 cases) and have a control mortality of 15% and bCPAP mortality of 7% based upon the literature. All analyses will have 95% confidence and 80% power.

Standard care case fatality rates were extrapolated from two sources, a publication under review for which the principal investigator is a senior author. This expected publication is a 12 year retrospective review of Malawi child pneumonia outcomes at the hospital level in which the mortality rate for severely malnourished children was approximately 15%, and overall hospital pneumonia mortality rate of about 4% (T. Colbourn, personal communication). We also used data extrapolated from a randomized study from 2011 in HIV-infected and HIV-exposed infants that provided severe pneumonia outcomes.[18] The hypoxemia arm sample size is achievable during the 24 month data collection timeline based upon our observational study data and the following calculations: 6,000 annual Salima hospital pediatric admissions (per Salima hospital data) x 20% prevalence of severe pneumonia without HIV or severe malnutrition (observational study data) x 11% severe hypoxemia prevalence (observational study data) = 132 cases/annually. The blood gas aims used a trial examining non-invasive positive pressure ventilation of children in a

resource-rich setting to determine sample size. (This is due to lack of similar data in Malawi.) The trial reported a pre-therapy mean (standard deviation) pCO_2 of 45 (11) and post-therapy mean pCO_2 of 39 (8).

V.2 STUDY DISSEMINATION

Results from the primary research study (randomized controlled trial) will be presented through at least one published peer-reviewed manuscript with detailed description of the background, methods, results, and conclusion. We expect to published at least one additional manuscript with the observational sub-study data. Study findings will also be presented to the Malawi MOH Senior Management and hospital staff at the site. Co-investigators plan on attending at least one international conference to disseminate the findings of the study.

VI.ETHICS AND CONSENT

- **Principles for Clinical Research**

The study will comply with the International Conference on Harmonization Good Clinical Practice E6 (ICH-GCP), all applicable regulatory requirements and Institutional Review Boards/Independent Ethics Committee reviews. All study staff will be trained and certified in the protection of human subjects.

- **Institutional Review Boards (IRBs) and Independent Ethics Committees (IECs)**

The IRBs of record for this clinical trial are the Johns Hopkins School of Medicine IRB and the Malawi National Health Sciences Research Committee (NHSRC). Reliance agreements will be sought from the IRBs for University of North Carolina-Chapel Hill IRB, Cincinnati Children's Hospital Medical Center, and Utah Primary Children's Hospital. A copy of the protocol, proposed informed consent forms, and other written participant information will be submitted to both Johns Hopkins and NHSRC for written approval. The investigators must submit and obtain approval from the IRB for all subsequent protocol amendments and changes to the informed consent document. The investigators will notify the IRB of serious adverse events according to the IRB requirements. The Principal Investigator and co-investigators are responsible for assuring that this protocol and the associated informed consent documents and study-related documents are approved by Johns Hopkins and NHSRC prior to implementation of the protocol. Any amendments to the protocol, informed consents, or other study-related documents must be approved by the IRB prior to implementation. The study will be conducted in full compliance with the protocol. Any deviations from or violations of the protocol will be documented and submitted to the IRB by investigators as required.

- **Informed Consent**

The site investigators will comply with regulatory requirements and adhere to GCP and to the ethical principles of the Declaration of Helsinki. This clinical trial will have an informed consent form (ICF) for **screening and enrollment** in compliance with relevant regulations. ICFs will be approved by IRBs before use. The consent forms will include the study purpose, the investigational device, procedure description and the risks and benefits of the study. This process will provide the information needed for LARs to determine whether to participate in the study. Questions are permitted and free information exchange is encouraged. If the LAR is illiterate, an independent witness will verify all the information read aloud is within the ICF. Both the caregiver and witness will sign the ICF in this scenario, and the caregiver will have the option of a providing

a thumb print rather than a signature. Study staff obtaining consent will sign and date the ICF. A signed and dated copy of the consent form will be given to the participant and this will be documented.

- **Participant Risk**

  - Randomization arms
    This is a randomized trial investigating the effectiveness of bCPAP for high risk WHO severe pneumonia. Death is the most important risk to subjects receiving bCPAP. However, bCPAP has been demonstrated to reduce mortality in neonates in low-resource settings such that the extension of this mortality effect to older age groups is reasonable. Alternatively it is possible that bCPAP is superior to standard care and that those children receiving standard care will have a higher risk of treatment failure and adverse outcomes including death. However, participation in this study affords the participants access to more attentive clinical care than is normal in this setting. For example, participants in either study arm will receive frequent clinician review, nursing review, and vital signs collection. Typically patients are reviewed clinically once per day and nursing support is characterized by medication administration only (not nasal suctioin or other respiratory supportive care). In some children the mask or prongs from bCPAP can be uncomfortable or in more rare cases can injure the child's nose if it does not fit correctly or is worn too long. Multiple mask and prong sizes will be available such that an appropriately sized mask or prong can be used to minimize this risk. In addition, children in this study will wear the bCPAP only during their hospitalization of approximately 2-5 days. This overall short duration reduces the risk of any long-term nasal injury. Every 4-6 hours the patient reassessed and this reassessment will include monitoring for any bCPAP-related nasal injury.

  - Coercion
    Caregivers may feel coerced to enroll in the study in order to receive care for their child within a research setting, which may be perceived as of a higher quality than the standard of care.

  - Specimen Collection
    The study involves blood specimen sampling at screening, and for a subset of patients also at enrollment and 1 and 6 hours after enrollment. When the blood is drawn, the child may feel mild discomfort and redness, pain, swelling, bruising, or an infection may occur where the blood is drawn. This is the same risk as in any routine blood draw.

  - HIV Testing
    The patient's caregiver may become embarrassed, worried, or anxious talking about HIV or waiting for their child's test results. It is possible that others may learn of the patient's participation here and think that they have HIV. This could lead to unfair treatment in the community.

  - **Protection against Risks**

  - Randomization arms

Safety monitoring for this study includes frequent clinical examination and monitoring every 4-6 hours, outcome assessment and a clinician or nurse on call via an emergency hotline, treatment and tracking of all AEs, and an external DSMB for regular review of cumulative safety and study conduct data.

  - Coercion

In order to minimize the risk of coercion, study staff will not be recruiting participants directly. Instead, SDH clinicians will inform caregivers about the study and refer only those who are interested. During the informed

consent process, study staff will emphasize that the child will receive medical care whether enrolled in the study or not.

- Specimen Collection

In order to minimizing the risks associated with phlebotomy, all study staff who will be collecting specimens from participants will be trained in the appropriate procedures and supervised accordingly.

- HIV Testing
  A trained HIV counselor or nurse will help the caregiver deal with any feelings or questions that they may have. The study team will make every effort to protect the caregiver's and child's privacy and confidentiality while hospitalized. The HIV counseling visit will take place in private.

- **Participant Benefits**

Direct benefits to participants of the randomized controlled trial include increased clinical supervision and care during the study period. Frequent clinical evaluations and monitoring are not included as standard of care, so trial participants will benefit from monitoring during their hospitalization. This supervision will make it more likely that a case of treatment failure is identified and appropriately managed. Participants will have access to a 24/7 phone number which is also not a part of standard of care.

If this trial demonstrates superiority of bCPAP, the results have great potential to inform and support national and international guidelines for treatment for childhood pneumonia. For example, bCPAP could be scaled up as a viable, evidence supported treatment modality for children 1-59 months old with WHO severe pneumonia in low-resource settings.

- **Participant Confidentiality**

The site investigators will maintain the child's confidentiality. Personal identifiers will not be included in any study reports. All study records will be kept confidential. Study procedures will be conducted to protect participant privacy and confidentiality to the fullest extent possible.

- **Participant Reimbursement**

Travel reimbursement will be provided to caregivers of randomized controlled trial participants to compensate them for the cost of transport after hospital outcome. Reimbursement will be appropriate to the cost of travel, payable at the end of the hospitalization. The reimbursement amount may be modified during the course of the study to reflect potential changes in travel costs. The study consent form will list the minimum amount to be paid in the local currency. Randomized controlled trial participants will not be responsible for paying for study-related care.

- **Storage of Specimens**

Specimens collected during the course of this research will not be stored. Any leftover samples not consumed during study-related diagnostic tests will be destroyed.

VII. POTENTIAL CHALLENGES

Anticipated implementation challenges to the successful outcome of the study include ensuring quality and consistency of implementation at the trial site. We plan to provide standardized training, supervision, and oversight to ensure quality and harmonized trial procedures.

Subcontracts: Collaborators are fundamental to the success of the proposal. The Lilongwe Medical Research Trust (UNC Project) was carefully selected based on qualification factors and the ability to help this project achieve its objectives. The subcontract with Lilongwe Medical Research Trust (UNC Project) will allow employment of dedicated project personnel including a study physician, clinical officer, nurse coordinator, nurse, HIV counselor, 3 vital sign assistants, and a data manager.

VIII.REFERENCES

1. Wang H, Liddell CA, Coates MM, et al. Global, regional, and national levels of neonatal, infant, and under-5 mortality during 1990–2013: a systematic analysis for the Global Burden of Disease Study 2013. The Lancet 2014;384:957-79.
2. Liu L, Oza S, Hogan D, et al. Global, regional, and national causes of child mortality in 2000–13, with projections to inform post-2015 priorities: an updated systematic analysis. The Lancet 2014.
3. Graham SM, Mankhambo L, Phiri A, et al. Impact of human immunodeficiency virus infection on the etiology and outcome of severe pneumonia in Malawian children. The Pediatric infectious disease journal 2011;30:33-8.
4. Graham SM, Duke T. Challenges to improving case management of childhood pneumonia at health facilities in resource-limited settings. Bull World Health Organ 2008;86:349-55.
5. Preidis GA, McCollum ED, Mwansambo C, Kazembe PN, Schutze GE, Kline MW. Pneumonia and Malnutrition are Highly Predictive of Mortality among African Children Hospitalized with Human Immunodeficiency Virus Infection or Exposure in the Era of Antiretroviral Therapy. The Journal of Pediatrics 2011;159:484-9.
6. Notterman DA, Greenwald Bm Fau - Di Maio-Hunter A, Di Maio-Hunter A Fau - Wilkinson JD, Wilkinson Jd Fau - Krasinski K, Krasinski K Fau - Borkowsky W, Borkowsky W. Outcome after assisted ventilation in children with acquired immunodeficiency syndrome. Crit Care Med 1990;18:18-20.
7. Essouri S, Durand P, Chevret L, et al. Optimal level of nasal continuous positive airway pressure in severe viral bronchiolitis. Intensive Care Med 2011;37:2002-7.
8. Teague WG. Noninvasive ventilation in the pediatric intensive care unit for children with acute respiratory failure. Pediatric Pulmonology 2003;35:418-26.
9. Nowadzky T, Pantoja A, Britton JR. Bubble Continuous Positive Airway Pressure, A Potentially Better Practice, Reduces the Use of Mechanical Ventilation Among Very Low Birth Weight Infants With Respiratory Distress Syndrome. Pediatrics 2009;123:1534-40.
10. Polin RA. Bubble CPAP: A Clash of Science, Culture, and Religion. The Journal of Pediatrics 2009;154:633-4.
11. van den Heuvel M, Blencowe H Fau - Mittermayer K, Mittermayer K Fau - Rylance S, et al. Introduction of bubble CPAP in a teaching hospital in Malawi.
12. Kawaza K, Machen HE, Brown J, et al. Efficacy of a low-cost bubble CPAP system in treatment of respiratory distress in a neonatal ward in Malawi. PloS one 2014;9:e86327.
13. McCollum ED, Smith A, Golitko CL. Bubble continuous positive airway pressure in a human immunodeficiency virus-infected infant. The international journal of tuberculosis and lung disease : the official journal of the International Union against Tuberculosis and Lung Disease 2011;15:562-4.
14. Walk J, Dinga P, Banda C, et al. Non-invasive ventilation with bubble CPAP is feasible and improves respiratory physiology in hospitalised Malawian children with acute respiratory failure. Paediatrics and international child health. 2014.
15. World Health Organization. Guidelines For The Management Of Common Childhood Illnesses: Second Edition. Geneva, 2013.
16. Programme UND. Country Profiles, Malawi. 2014.
17. UNAIDS. Country Progress Reports, Malawi. 2014.
18. McCollum ED, Preidis GA, Maliwichi M, et al. Clinical versus rapid molecular HIV diagnosis in hospitalized African infants: a randomized controlled trial simulating point-of-care infant testing. Journal of acquired immune deficiency syndromes 2014;66:e23-30.

APPENDICES

APPENDIX I: SCHEDULE OF STUDY VISITS

	Screening (Day 1)	Enrollment (Day 1)	Hospital course (4-6 hourly monitoring)	Hospital outcome	30 day phone follow-up
Informed Consent	✓	✓			
Participant ID	✓	✓	✓	✓	✓
Eligibility Assessment	✓	✓			
Demographics		✓			
Locator Information (phone number)		✓	✓	✓	✓
Randomization		✓			
Travel reimbursement				✓	
Medical History	✓	✓	✓	✓	✓
Targeted Physical Exam	✓	✓	✓	✓	✓

APPENDIX II: SAMPLE COLLECTION AND LABORATORY EVALUATIONS

Specimen for Diagnostic	Screening	Enrollment (Day 1)	1 and 6 hours after enrollment	Hospital outcome	30 day phone follow-up	Laboratory
HIV antibody test	✓					SDH
HIV DNA PCR (<12 months old and mother HIV-infected ***or*** child HIV antibody positive)		✓				KCH
Full blood count (FBC)		✓				SDH
Malaria test (mRDT)		✓				SDH
Blood gas/lactate (282 patients only)		✓	✓			SDH
Biomarkers		✓				Cincinnati (USA)

APPENDIX III: STUDY REQUIREMENTS AND TRAINING

Additional study requirements not already described in the protocol are summarized below.

- **Personnel**

  The study team on-the-ground will consist of employees in the following capacities:

  Study physician (UNC Project): The UNC Project study physician is to be determined. The salary assumption was calculated from the UNC Project salary scale for 2014 at 20% effort. The study physician is responsible for in-person weekly supervision of the field site to ensure ongoing study activities and coordination of weekly teleconferences between the PI, nurse coordinator, and clinical officer. The study physician is also responsible for the monthly study report.

  Study nurse coordinator (UNC Project): The study nurse coordinator is an employee of UNC Project and is to be determined. The salary assumption is based upon full 100% effort from the 2014 UNC Project salary scale. The nurse coordinator is responsible for day-to-day study coordination and supervision including patient screening, enrollment, and protocol adherence by study staff.

  Study clinical officer (UNC Project): The UNC Project study clinical officer is to be determined. The salary assumption is also based upon 100% effort from the 2014 UNC Project salary scale. The clinical officer's responsibilities include daily patient enrollment and care according to the study protocols.

  Study nurse (UNC Project): The study nurse is also a UNC Project employee and is to be determined. The salary assumption is per 100% effort from the 2014 UNC Project salary scale. The nurse's responsibilities will be for daily patient enrollment and care per the study protocols.

  HIV counselor (UNC Project): The HIV counselor is an employee of UNC Project and is to be determined. The salary assumption is for full 100% effort from the 2014 UNC Project salary scale. The HIV counselor's responsibilities will be for HIV counseling and testing of all study subjects per the trial protocols.

  Vital sign assistants (3) (UNC Project): The vital sign assistants are employees of UNC project and are to be determined. The salary assumption is for full 100% effort per the 2014 UNC Project salary scale. The vital sign assistant's responsibilities include assisting the study nurses and clinical officer in the care of patients including but not limited to routine collection of vital signs, patient respiratory support, overnight patient supportive care, and maintenance of respiratory equipment.

  Data manager (UNC Project): The data manager is an employee of UNC Project and is to be determined. The salary assumption is for 20% effort per the 2014 UNC Project salary scale. The data manager will be responsible for data quality assurance including database development with Redcap, double data entry, producing monthly data quality indicators.

  Government staff at the study site hospital will also be engaged with this study. SDH service providers will be responsible for recruiting of study participants.

- **Training**

All study staff will be trained in the Protection of Human Subjects prior to any interactions with study participants. Additionally, before the study starts, all study staff will attend a 2-day study-specific training to review all study procedures, including the study protocol, SOPs, data collection tools, informed consent process, reporting requirements, and safety monitoring. Refresher trainings on the identification of pneumonia will be scheduled at least once per year and will include updates from the study monitor reports. Trainings will be conducted by a qualified clinician, as appropriate for the training material.

Government staff at SDH will be sensitized to this study prior to the study start. Refresher trainings will be held periodically, at least once every year.

- **Supplies**

  Supplies for this study include the following:
  - bCPAP equipment
  - Oxygen concentrators
  - Digital stethoscope
  - Video recorder
  - Laptop for study staff
  - Printer
  - Respiratory rate counters
  - Portable pulse oximeter
  - Scale
  - Height board
  - Partitions/privacy screens for the pediatric ward
  - Communication equipment such as cellphone accessories, airtime, and internet sticks
  - Standard office supplies, including binders, paper, pens

- **Transportation**

  Study participants will be expected to provide their own transportation home but will receive a travel reimbursement.

- **Space**

  The study area for screening, enrollment, and ongoing care will be located in the pediatric ward of SDH. The hospital has provided the study with a private area in the pediatric ward and other study-related activities. Additional office space for data management and the study physician will be provided at the UNC Project office in Lilongwe.

SOP: Oxygen wean

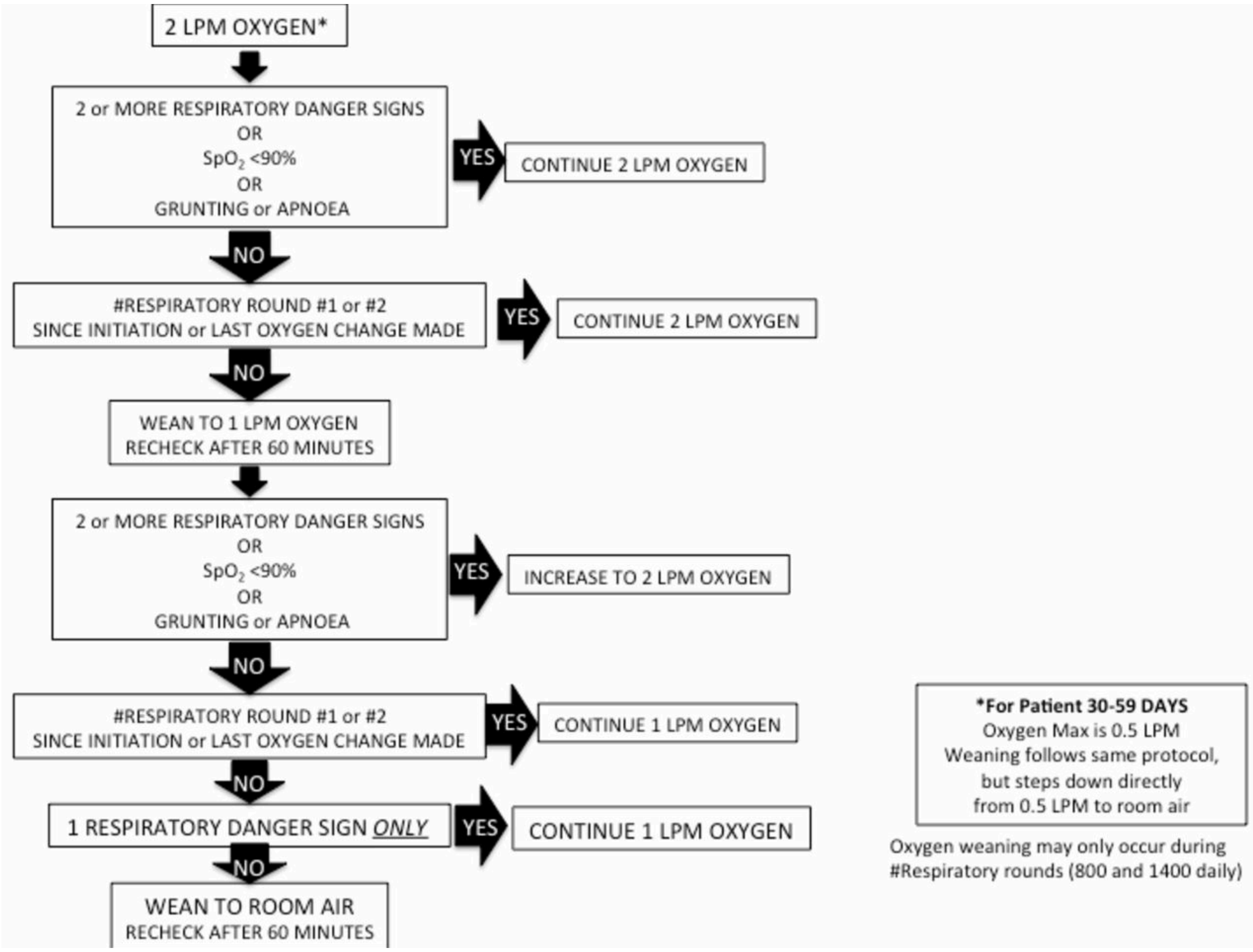

SOP: bCPAP wean

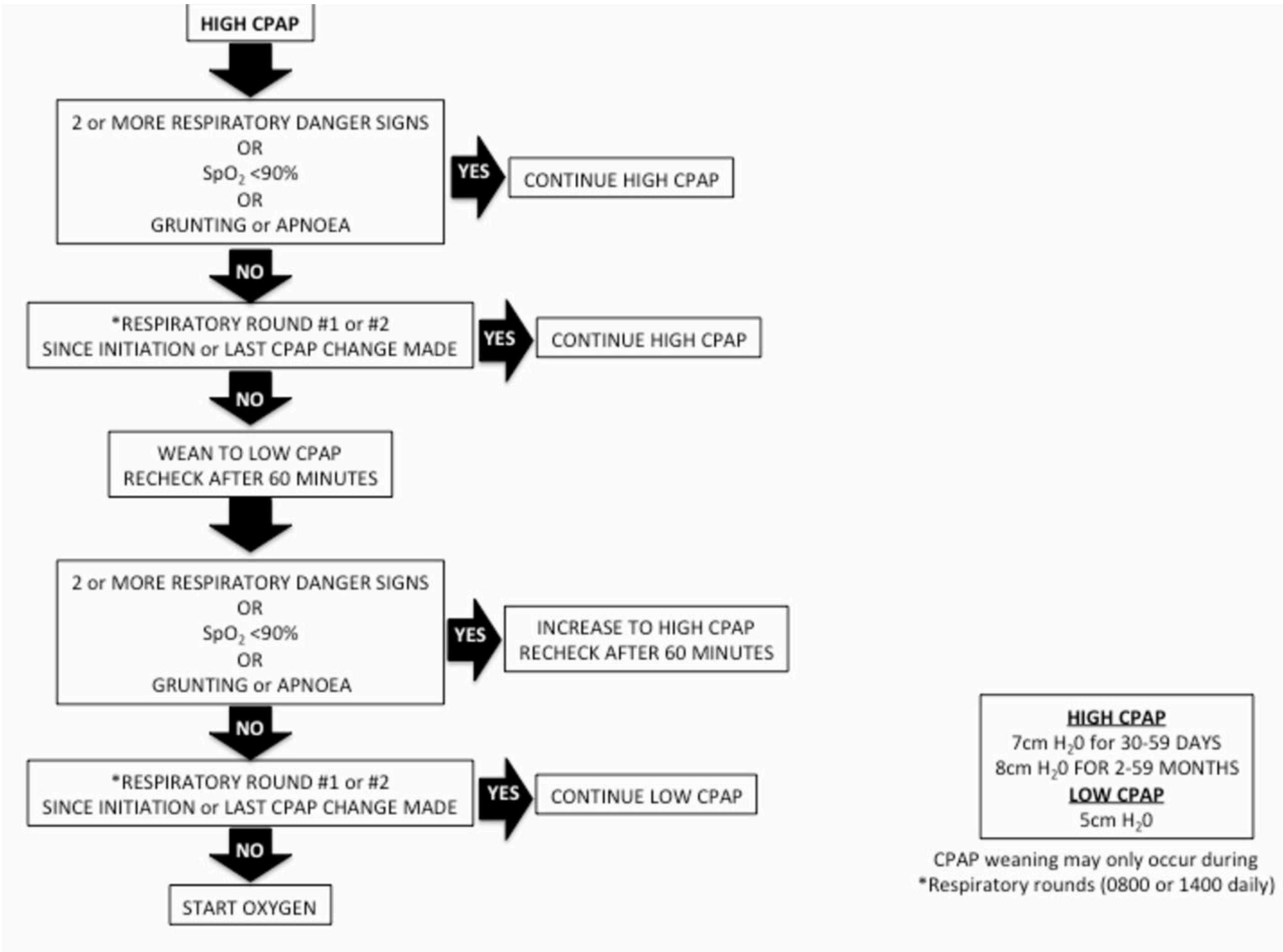

SOP: Feeding/fluids for Severe malnutrition

November 2015

Malnutrition Feeding Day 1 for children with WHO severe malnutrition

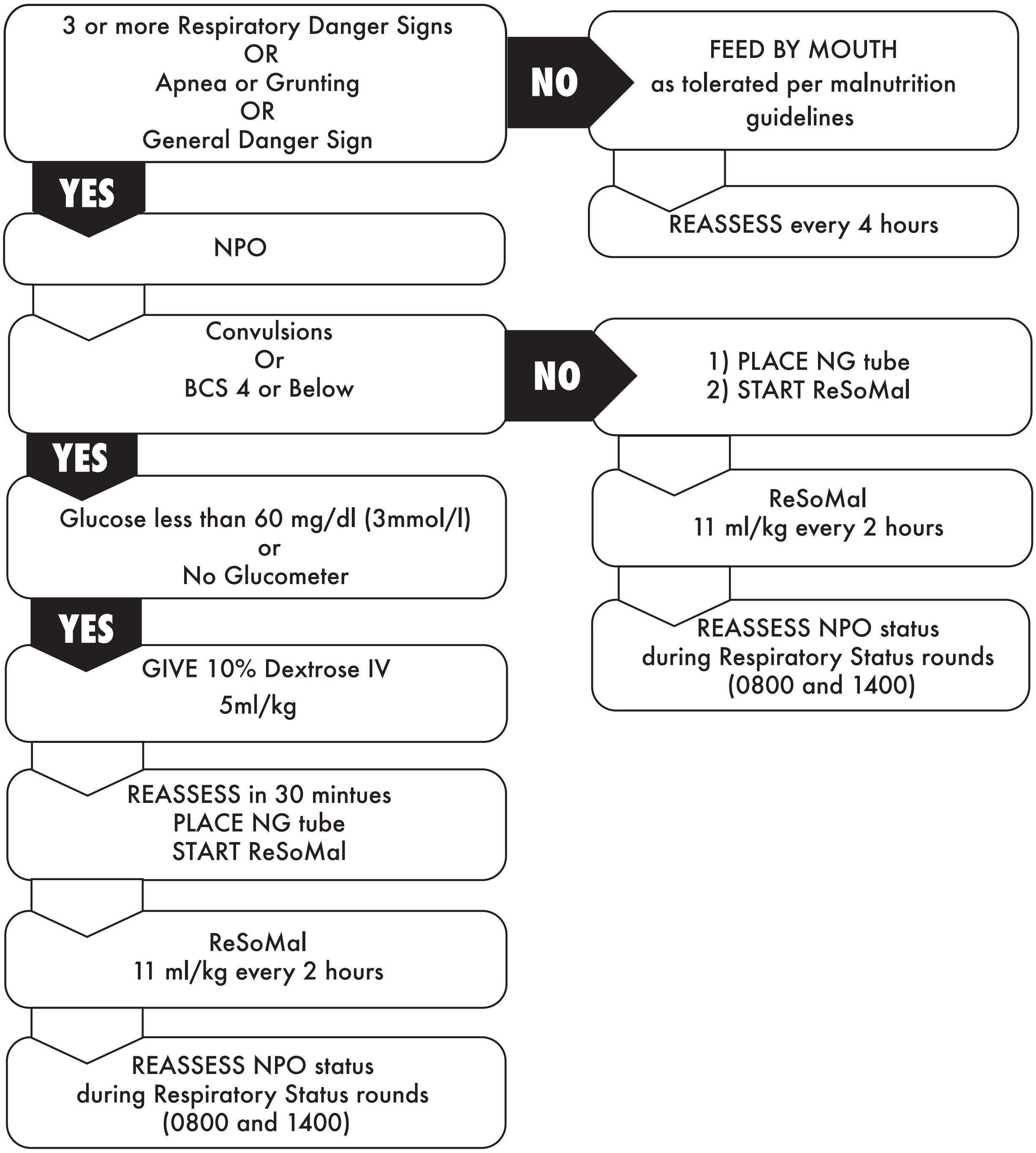

SOP: Feeding/fluids for No severe malnutrition

November 2015

NO Malnutrition
Feeding Day 1

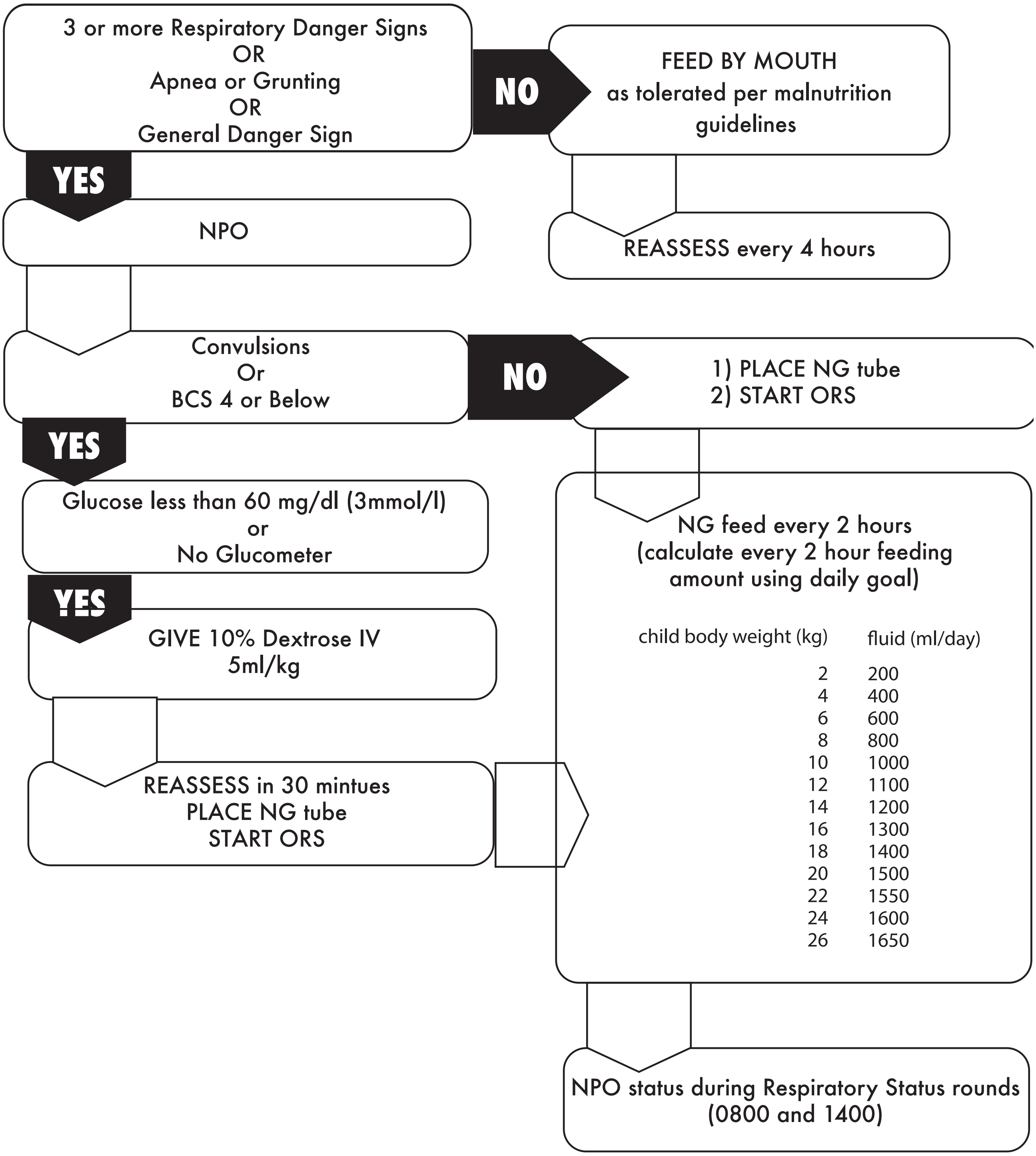

SOP: Respiratory supply decontamination procedures for Salima District Hospital, Salima, Malawi

Process of cleaning instruments

- Wear personal protective attire
- Utilize soft brush detergent and running water
- Scrub instruments and other items under water completely removing blood and other foreign matter
- Disassemble instruments and other items with multiple parts and clean in the grooves teeth and joints with a brush
  -Rinse the instruments thoroughly with clean water
  -Allow instruments to air-dry or dry with a clean towel
- Perform hand hygiene after removing gloves

HOW TO DECONTAMINATE

- IMMEDIATELY AFETR A PROCEDURE AND REMOVE OF GLOVES
- PLACE ITEMS IN 0.5% CHLORINE SOLUTION
- ALLOW TO SOAK FOR 10-15 MINUTES
- REMEMBER TO DIP YOUR GLOVED HANDS IN 0.5% CHLORINE SOLUTION BEFORE REMOVING YOUR GLOVES,REMOVE GLOVES BY INVERTING .
- DEPOSITE THEM INTO A HAZARDOUS WASTE CONTAINER FOR GLOVES THAT WILL BE REPORCESSED (UTILITY GLOVES)
- AFTER 10 MINUTES , SOAKING IN JIK REMOVE INSTRUMENTS , DO NOT SOAK INSTRUMENTS MORE THAN 10 MINUTES
- IMMEDIATELY PLACE THEM IN SOAPY WATER FOR CLEANING.

SALIMA DISTRICT HOSPITAL IP

PREPARATION OF 0.5% CHLORINE SOLUTION FOR DECONTAMINATION

LIQUID CHLORINE

- **If using JIK (3.5%)**
  - Mix 1 part of JIK (bleach) with 6 parts of water.
- **If using JIK (5%)**
  - Mix 1 part of JIK (bleach) with 9 parts of water.

POWDER CHLORINE

- **If using Calcium Hypochlorite (35%)**
  - Weigh 14g of bleach powder for 1L of water.
- **If using Calcium Hypochlorite (70%)**
  - Weigh 7g of bleach powder for 1L of water

<u>NOTE:</u>

- New chlorine solution should be prepared at the beginning of each new day.
- Instruments and other items should be soaked in 0.5% chlorine solution for 10 min b4 cleaning them.
- After 10 min remove instruments and other items from the solution and rinse them with clean water.
